# Trace metal contamination from water and soil to vegetables and associated human health risks

**DOI:** 10.1039/d6ra05726h

**Published:** 2026-09-08

**Authors:** Bashdar Abuzed Sadee

**Affiliations:** a Department of Food Science and Technology, College of Agricultural Engineering Sciences, Salahaddin University-Erbil, KRG Iraq bashdar.sadee@su.edu.krd bashdarabuzed@gmail.com; b Department of Nutrition and Dietetics, Cihan University-Erbil Erbil Iraq

## Abstract

Vegetables represent potential pathways for increased heavy metal exposure in humans. Inductively coupled plasma mass spectrometry (ICP-MS) was applied to estimate 19 trace metals in various parts of beetroot, onion, rice, spinach, parsley, rhubarb, dill, basil, and radish plants as well as in the irrigation water and corresponding cultivated soils. Parsley, rhubarb, and basil showed the same distribution pattern for most elements as follows: leaf > stem > root. Likewise, onion, spinach, and radish demonstrated similar trends for most metals as follows: root > stem > leaf. The fraction of metals present in the plant and the soil-to-plant transfer factor (TF) indicated that the absorption and movement of metals were significantly influenced by both the specific element and the plant compartment, with the majority of metals exhibiting low transfer rates, although certain metals demonstrated greater accumulation in particular organs, as the TF for most metals was less than 1. The HQ values of the consumable different organs of vegetables/plans of this study revealed distinct variations in the compartmentalization of metals across the examined vegetables/plants. As and Cr were the predominant contributors to non-carcinogenic risks across all consumable parts exceeding the established USEPA threshold for the majority of compartments of vegetables/plants, with the highest HQ values recorded in the edible parts of rice grain (54 for adults and 62 for children) for As and onion leaf (9.4 for adults and 10.7 for children) for Cr. The hazard index (HI) values for heavy metals in different parts of the selected vegetables and plants, except for the stems and leaves of rhubarb, were higher than the USEPA-established threshold of 1, indicating potential health risks for adults and children. The carcinogenic risk (CR) was very diverse among plant species and tissues. The CR values for As in all analysed vegetables, Cr (except the edible parts of beetroot) and Ni (skin, core, and leaf of beetroot and rice grain) exceeded the probable carcinogenic threshold (>1.0 × 10^−4^). The findings regarding Pb and Cd indicate that most levels are within tolerable or negligible risk limits for both adults and children. This implies a potential carcinogenic risk, though it is accompanied by some uncertainty.

## Abstract 2

Vegetables represent potential pathways for increased heavy metal exposure in humans. Image created with the assistance of Google Gemini.

## Graphical abstract

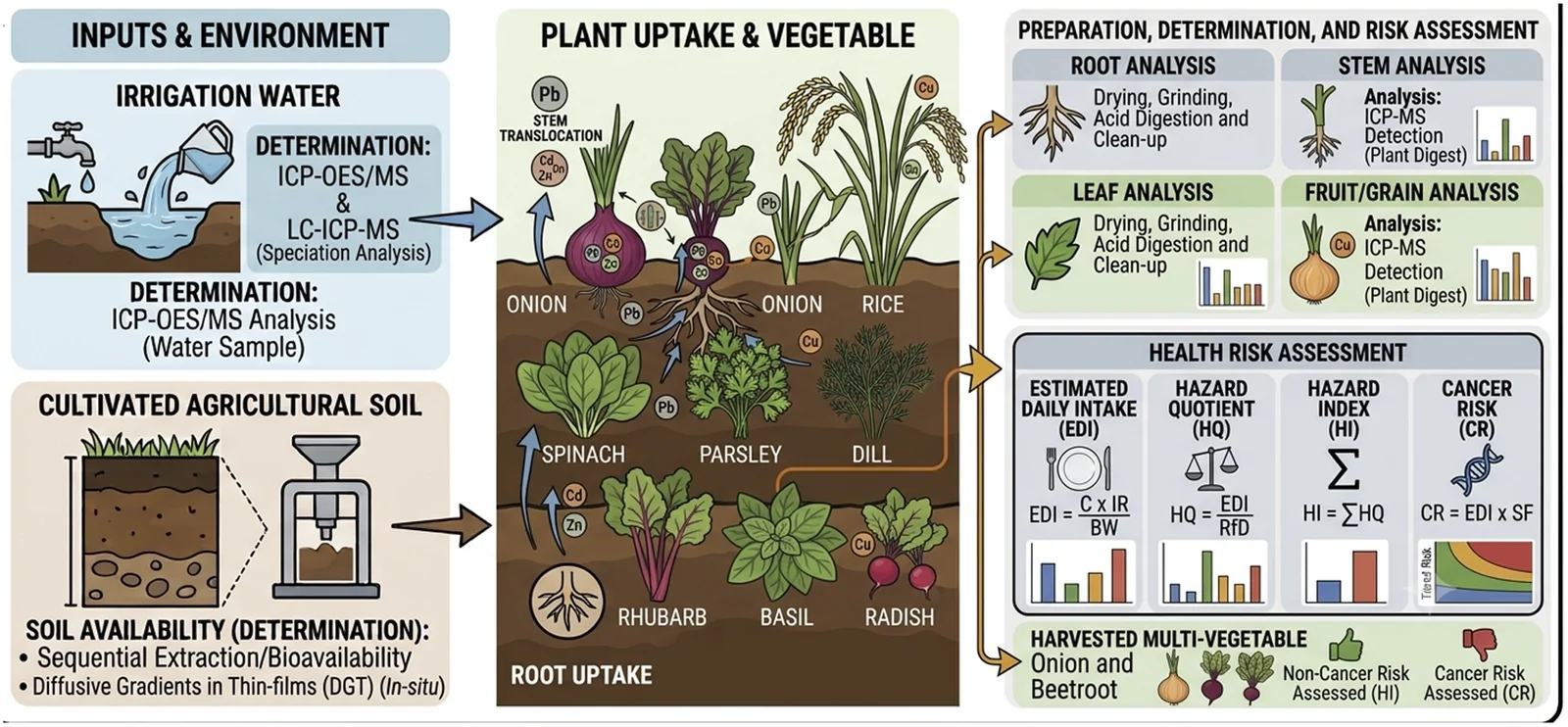

## 1 Introduction

Vegetables constitute a significant portion of the daily diet for most people worldwide. While the consumption of leafy greens has increased among general public, these vegetables are often contaminated with heavy metals such as As, Se, Cd, Cr, Co, Cu, Ni, and Pb. It is well recognized that heavy metal contamination has emerged as a global concern due to their considerable ability to contaminate water, vegetables, fish, and aquatic and terrestrial plants and thereby enter the food chain, especially through plant-based foods.^1,2^

Heavy metals are naturally occurring elements in the Earth's crust and may be released through natural processes; however, anthropogenic activities are largely responsible for their pollution. Key contributors include mining operations, improper disposal of industrial effluents, and various industrial and agricultural practices.^2^ Overall, wastewater irrigation, solid waste dumping, sludge application, agricultural practices, and human activities associated with industrial, mining, and domestic sources are the major contributors to the introduction of heavy metals into irrigated soils.^3^

Heavy metals are generally classified as essential (*e.g.*, Fe, Mn, Cu, Se, and Zn), potentially essential (*e.g.*, Ni, V, and Co), and toxic (*e.g.*, As, Cd, Pb, and Hg).^4,5^ Several factors affect the transfer of heavy metals from soil to plants, such as the type of sources, seasonal variations, application rates, soil pH, temperature, moisture, redox potential, soil texture, organic matter content and the chemical form of the metals, nutrient levels, plant species and total concentration of metals in soil.^6,7^ Heavy metals can enter the food chain either directly by consumption of contaminated food or indirectly through plants absorbing these metals, which then accumulate in food products that are subsequently eaten.^8^

It is well known that trace elements in the environment are hazardous to human health, and many studies have shown their negative impact on both ecosystems and food quality.^9^ The uptake and accumulation of heavy metals in vegetables can alter plant phytochemistry, plant physiology and morphology, affecting growth, water and nutrient balance, and overall nutritional functions, causing changes in active compounds and secondary metabolites. Symptoms of toxicity in plants include chlorosis, stunted growth, root browning, and eventual death.^10,11^

In humans, consuming plants contaminated with heavy metals can cause serious health-related issues. As is a significant contributor to environmental pollution due to its high toxicity and carcinogenic properties, and it is linked to cardiovascular and nervous system disorders, skin cancer, and organ enlargement;^12,13^ Cd intake may cause respiratory, cardiac, and renal problems; Cr exposure can result in renal necrosis, dermatitis, lung cancer, and nasal damage, though it also plays roles in biochemical processes like insulin function.^14^ Exposure to arsenic poses a health risk to millions of people worldwide; Co is used in the treatment of anemia in pregnant women, as it stimulates red blood cell production and helps prevent conditions such as severe anemia, extreme fatigue, shortness of breath, and hypothyroidism caused by Co deficiency. However, excessive exposure to Co may lead to adverse effects, including angina, asthma, cardiomyopathy, polycythemia, and dermatitis.^15^ The transfer of heavy metals from water to soil, then into plants, and ultimately to humans, represents a critical pathway of environmental contamination and food chain exposure, posing significant risks to human health through dietary intake. Understanding and monitoring this transfer is essential for ensuring metabolic pathway, food safety and environmental sustainability.

Several studies have explored the transport and accumulation of heavy metals in plants. However, most of these articles have studied single edible plant compartments or a limited number of metals, with limited integration of soil, irrigation water, and environmental plants.^16,17^ Despite this, relatively few articles have simultaneously measured trace metal concentrations across soil, irrigation water, and different plant compartments, hampering the capability to fully comprehend metal transfer mechanisms and associated exposure risks.

Few research studies are available on heavy metal contamination in vegetables. There is a limited availability of precise data on the integrated investigation of total metal concentrations, possible mobility and associated dietary health concerns in vegetables grown and consumed in Erbil city. Therefore, the present work combines multi-element (19 metals) analysis with BCR sequential extraction to evaluate the potential mobility and bioavailability of metals in plants, food intake and associated health risks. The integration of this technology provides a better understanding of the metal contamination in vegetables and its possible implications on the food safety and human health.

The vegetables for this study were selected based on their high consumption in Kurdistan region of Iraq. These vegetables contribute markedly to daily vegetable intake. It provides an essential database for the local authority to take action and regulate the environmental monitoring of the selected vegetables and their associated soils and water by comparing the findings of these results against standard regulations set by international organizations such as the WHO/FAO Codex Alimentarius maximum levels, the United States Environmental Protection Agency (USEPA), and the European Commission.

The aims of the study were to determine the concentrations of Al, Ag, As, Ba, Ti, V, Cr, Mn, Fe, Co, Ni, Cu, Zn, Se, Mo, Cd, Sb, Tl and Pb in irrigation water, cultivated soil and different parts of selected beetroot, onion, rice, spinach, parsley, dill, rhubarb, basil, and radish vegetable from Erbil City-Iraq. This study helps to understand the metabolism pathways of these heavy metals in selected vegetables and plants from water, soil and their organs. It also extends to assess the human health risks employing established standard indices approaches proposed by the FAO/WHO and USEPA using the estimated daily intake (EDI), HQ, HI and CR calculations associated with heavy metal consumption from these vegetables by adults.

## 2 Materials and methods

### 2.1 Chemicals

All chemicals employed in this investigation were of analytical grade and were used without further purification. Unless otherwise mentioned, Milli-Q water (18 MΩ cm) was employed for the preparation of all solutions. A multi-element stock solution (100 µg mL^−1^ in 5% HNO_3_) obtained from CPI International, USA, was used to prepare Al, Ag, As, Ba, Ti, V, Cr, Mn, Fe, Co, Ni, Cu, Zn, Se, Mo, Cd, Sb, Tl and Pb standard solutions. Indium (In), iridium (Ir), and ammonium dihydrogen orthophosphate were supplied by VWR International (MERCK, UK). HNO_3_ (70%, w/w) and H_2_O_2_ (37%) were supplied by Merck (Poole, UK). l-Ascorbic acid, HCl (37%), and H_3_PO_4_ (87%) were purchased from Fisher Scientific (Loughborough, UK).

GBW10015-spinach, BCR701, and loam soil ERM-CC141 were used as certified reference material (CRM) and purchased from the Institute of Geophysical and Geochemical Exploration (China), Sigma-Aldrich (UK), and European Commission (Belgium), respectively.

### 2.2 Instrumentation

The total concentrations of Al, Ag, As, Ba, Ti, V, Cr, Mn, Mo, Fe, Co, Ni, Cu, Zn, Se, Cd, Sb, Tl and Pb in different parts of dried beetroot, onion, rice, spinach, parsley, dill, rhubarb, basil, and radish, cultivated soil, and irrigation water were measured using an Agilent 7500 ICP-MS instrument (USA), under the operating conditions described in SI Table 1. A mass spectrometer was configured to register counts per second by employing the peak transition mode to analyze the specific isotopes: ^27^Al, ^107^Ag, ^75^As, ^137^Ba, ^111^Cd, ^59^Co, ^52^Cr, ^65^Cu, ^55^Mn, ^95^Mo, ^60^Ni, ^208^Pb, ^56^Fe, ^121^Sb, ^78^Se, ^47^Ti, ^205^Tl, ^51^V, and ^66^Zn.

### 2.3 Sample collection

Beetroot, onion, rice, spinach, parsley, dill, rhubarb, basil, and radish consumed in Erbil City were collected as samples. In addition, rice is a staple grain in the Kurdish diet, especially among the Erbil residents. Three individual samples were collected for each of the nine varieties of plants/vegetables (beetroot, onion, rice, spinach, parsley, dill, rhubarb, basil, and radish). Soil samples, irrigation water, and organs of plants and vegetables (including root, stem, leaf, bulb, and grain) were collected from two different agricultural locations in Erbil City, Iraq, in April 2025. The majority of rice ingested by Kurdish individuals is cultivated in a renowned agricultural region celebrated for its indigenous rice varieties frequently utilised in Kurdish cuisine and distinctive rhubarb species peculiar to the locale. This district is referred to as Akree. It is renowned for the rice paddy fields in northwest Erbil. The further two agricultural sites are in proximity to Erbil City and consistently provide these vegetables to the local market. Beetroot, onion, rice, spinach, parsley, dill, basil, and radish samples, their cultivated soils, and irrigation water samples were collected from two different farms in south Erbil city. Rice samples with their cultivated soil and water were collected in plant and vegetable samples were stored in sealed containers, rinsed with Milli-Q water, and transported in a cooled plastic box. They were frozen at −40 °C for 12 h, a freeze drier was used to dry all plants and soil samples as well at the same temperature for 48 h. The dried plants and soils were cautiously ground with an agate mortar and pestle, sieved through a 180 µm nylon mesh, and stored in brown glass bottles within a desiccator to preserve them from light and moisture until the day of analysis.

### 2.4 Water sample

HDPE tubes were employed to store the irrigated water samples collected from an on-site farm. In order to avoid any oxidation and change in heavy metal composition, the pH of water samples was maintained at 2 using HNO_3_. ICP-MS was employed to measure total metals in this study.

### 2.5 Total heavy metal content in soil using aqua regia digestion

An aqua regia protocol adapted from LGC was employed to digest the soil samples from the BCR fractionation for measuring total heavy metals. Acid extraction was only applied to soil samples with a 180 µm sample size. First, 0.5 g of the soil sample was transferred into a clean digestion tube. To digest the soil samples, 8 mL of HCl (35%) and 2 mL of HNO_3_ (70%) were introduced, and the soil samples were kept at room temperature for minimum one hour to facilitate the oxidation of readily degradable materials. A Tecator digestion block (Digestion system 12, 1009 digester) was used to digest the soil samples at 110 °C for two hours. The digested soil samples were cooled, and the extracts were transferred to pre-cleaned volumetric flasks (100 mL capacity), followed by the addition of deionized water to the mark. For every digestion batch, triplicates of the cultivated soil sample, loam soil (ERM-CC141) CRM, and procedural blank were analysed using the same procedure without the sample.

### 2.6 Total metal determination in plant samples

Before use, digestion tubes were thoroughly rinsed by soaking in 5% Decon 90 (Merck), prepared with Milli-Q water, for 24 h and then in 3% (v/v) HNO_3_ solution, also prepared with Milli-Q water, for another 24 h. Plant samples were acid-digested using a microwave designed by Mars Xpress lab station (CEM, USA). A triplicate of 0.25 g of freeze-dried different parts of plant samples were introduced into individual Teflon reaction tubes (100 mL capacity) and covered with Teflon caps. For complete digestion, 5 mL of concentrated HNO_3_ (70%) and 2 mL H_2_O_2_ (30%) were introduced into the tubes and covered with caps made from Teflon. The power of microwave was set at 1600 watt and the digestion process was completed within 43 minutes, involving two stages. In the first batch, within 15 minutes, the temperature was increased to 160 °C and maintained for 5 minutes. In the second step, within 8 minutes, the temperature was increased to 200 °C and maintained for 15 minutes. Once the digestion was completed, the tubes with their contents were left to cool to ambient temperature. Then, the digested samples were completely transferred to volumetric flasks (25 mL capacity) and diluted to the mark with dilute HNO_3_ (2% (v/v) prepared using Milli-Q water). Then, In and Ir were added as internal standards to achieve a final content of 10 µg L^−1^.^18–20^ A procedural blank sample was also established, employing the same procedure omitting the analytes of interest. For the validation of the applied procedure for the total metal determination in different parts of beetroot, onion, rice spinach, parsley dill, rhubarb, basil, and radish, GBW10015-spinach and NIES-high Cd level were also concurrently analysed for the samples using this method.

### 2.7 Sequential extraction method for total metal determination in soil

A modified three-step Bureau Communautaire de Référence (BCR) sequential extraction procedure was used to extract the extractable trace metal levels in the soils, which were used to cultivate beetroot, onion and rice samples.^21^ The steps of sequential extraction are detailed as follows:

#### 2.7.1 Stage one (acid-leachable fraction–carbonate-bound)

Triplicates of 1.0 g of freeze-dried soil of each sample were placed in separate polypropylene centrifuge tubes. Then, 40 mL of acetic acid (0.11 mol L^−1^) was introduced into each vessel. The samples were agitated continuously for 16 hours at room temperature (22 ± 5 °C). A centrifuge was used to separate the extract from the solid phase within 20 minutes at a rate of 3000 rpm. The extracts were quantitatively transferred into another fresh polypropylene centrifuge tube and placed in a refrigerator at 4 °C prior to instrumental analysis. Deionized water (10 mL) was applied to wash the residual fraction. The samples were agitated for 15 minutes. The supernatants were discarded carefully after separation from the residual solid fraction using a centrifuge (3000 rpm for 20 minutes).

#### 2.7.2 Stage two (reducible fraction–bound to Fe and Mn oxides)

First, 40 mL of hydroxylamine hydrochloride (0.5 mol L^−1^) was added to the residual fraction obtained in step 1. The pH of the solution was modified to 1.5 using HNO_3_ (2 mol L^−1^). The tubes were agitated overnight (16 hours at 22 ± 5 °C under a continuous rotational speed of 30 ± 10 rpm). The same procedure as step 1 was used to separate the extracts from the residual fraction, and the extract was kept in a refrigerator until analysis. Prior to step 3, the residual solid obtained at this stage was washed as explained in step 1.

#### 2.7.3 Stage three (oxidisable fraction–bound to organic matter and sulfides)

First, 10 mL of aliquots of H_2_O_2_ (8.8 mol L^−1^, pH = 2) were added in small portions to the centrifuge tube containing residual fraction remaining in stage 2. The tubes were sealed and kept at ambient temperature for 1 hour with occasional agitating. The vessels were heated (85 ± 2 °C) for 1 hour in a water bath and agitated in the first 30 minutes. The uncapped tubes were heated to reduce the volume of the solution to up to 2–3 mL. To each of the tubes, 10 mL of aliquots of H_2_O_2_ (8.8 mol L^−1^, pH = 2) were added, and the capped tubes were again heated (85 ± 2 °C) for 1 hour in a water bath in order to reduce the volume almost to dryness. The tubes with their contents were kept at room temperature for cooling, and then 50 mL of ammonium acetate (1.0 mol L^−1^, pH regulated to 2 with concentrated HNO_3_) were added. The tubes were agitated overnight (16 hours) at room temperature. The extract was subsequently separated from the residual solid using a centrifuge and kept at 4 °C until the day of analysis.

### 2.8 Limit of detection

Eqn (1) was used to calculate the limit of detection (LOD) of the instrument, which is 3.3 times standard deviation (SD) of six blank (*b*) absorbance replicates divided by the slope (*m*) of the calibration curve. The LOD values for ICP-MS (in mg kg^−1^) are as follows: Al (0.019), Ag (0.015), As (0.008), Ba (0.018), Cd (0.007), Co (0.015), Cr (0.031), Cu (0.012), Fe (0.015), Mn (0.019), Mo (0.012), Ni (0.016), Pb (0.002), Sb (0.01), Se (0.007), Ti (0.06), Tl (0.01), V (0.017) and Zn (0.006), all of which are suitable for detecting trace minerals in water, soil and different parts of beetroot, onion, rice, spinach, parsley, dill, rhubarb, basil, and radish samples under study.

1 LOD = 3.3 × SD*b*/*m*

### 2.9 Transfer factor (TF)

To determine the actual amount of metal absorption by each vegetable, the TF was computed using eqn (2).^22^ The TF represents the ratio of the concentration of a particular pollutant in the plant to the concentration of the same pollutant in the soil. It is important to note that the calculation of the TF requires consideration of each plant type with its corresponding soil sample.2
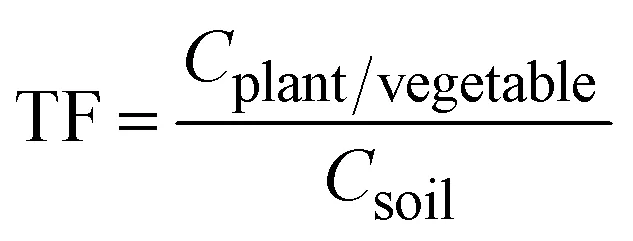
*C*_plant/vegetable_ is the metal concentration in the plant/vegetable in mg kg^−1^ and *C*_soil_ is the metal concentration in the cultivated soil associated with the vegetable/plant.

### 2.10 Health risk assessment

In this study, EDI, HQ, HI, and CR were used to estimate the health risks associated with heavy metals in adults from eating edible parts of beetroot, onion, rice, spinach, dill, parsley rhubarb, basil, and radish.

#### 2.10.1 EDI

Eqn (3) was used to determine the quantity of EDI for various parts of beetroot, onion, and rice:^23^3
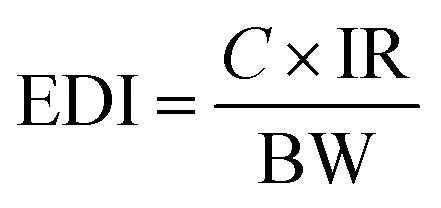
where *C* is the mean concentration of heavy metals (mg kg^−1^) in the vegetable samples subjected to analysis in this experiment. According to the regional research, the vegetable ingestion rate (IR) was 0.325 kg per person per day, while the USEPA survey indicated that it was 0.150 g per person per day for youngsters.^24^ BW refers to the body weight, anticipated to be 32 kg for toddlers and 79.1 kg for adults.^25,26^

#### 2.10.2 HQ

The HQ is an index used in non-carcinogenic risk assessments to estimate the potential for adverse health effects due to long-term exposure to a certain pollutant or chemical through the intake of edible sections of vegetables and rice. If the HQ value is 1 or less, the exposure is not expected to result in adverse impacts. However, if the HQ value is greater than one, it suggests a possible risk of non-carcinogenic health problems and should be investigated or treated further. The HQ value was calculated by dividing the EDI by the reference dosage (RfD), as given in eqn (4):^27^

4 HQ = EDI/RfD

The RfD values for Al, Ag, Ba, Cd, Co, Cr, Cu, Mn, Ni, Pb, Fe, Se, Si, V, and Zn, sequentially, are presented in SI Table 2.

#### 2.10.3 HI

The HI is defined as the sum of multiple HQs for different chemicals or exposure routes. It is used to assess the combined health effects of various chemicals or contaminants that affect the same organ or system. When the HI value exceeds one, there is an increased likelihood of potential health risks arising from the cumulative exposure.

The HI was calculated using the mean daily vegetable intake for adults to estimate the potential adverse health effects of cumulative exposure of the vegetable and rice grain to different heavy metals in this study. Eqn (5) was applied to determine the HI by combining the HQ of each separate metal in different parts of beetroot or onion or grain of rice:^23^5
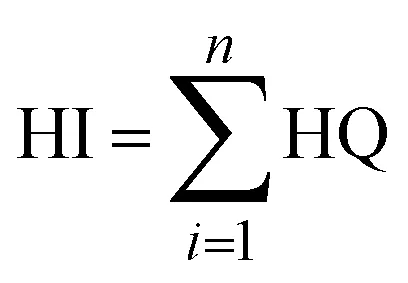


#### 2.10.4 CR

Cancer risk (CR) can be described as a theoretical estimate of the likelihood that an individual will develop cancer during their lifetime due to exposure to carcinogenic substances like heavy metals. Eqn (6) was applied to estimate the CR linked to the consumption of each heavy metal in the selected edible parts of vegetables and rice grain:^28^

6 CR = (EF × ED × FIR × *C* × SF)/BM × AT

where EF denotes the exposure frequency (365 days per year), ED is the exposure duration (24 years for adults),^24^ FIR is the average daily vegetable eaten in kg per day and *C* is the content of metals in the food (mg kg^−1^). AT refers to the average time for carcinogenic effects, calculated as 365 days per year multiplied by the ED, which is 25 550 days for adults. The oral carcinogenic slope factors (SF) for As, Cd, Cr, Ni, and Pb were obtained from the Integrated Risk Information System (IRIS), and are listed in Table 2. The CR values ranging from 1.0 × 10^−6^ to 1.0 × 10^−4^ are generally considered acceptable, a CR value below 1.0 × 10^−6^ is deemed negligible, while the CR values exceeding 1.0 × 10^−4^ indicate a potentially significant cancer risk.^29,30^

### 2.11 Statistical analysis

Data analysis was conducted using the SPSS software with a one-way ANOVA.^31^ Results are presented as means and standard errors using descriptive statistics. Duncan's test was applied to determine significant differences among the different parts of the analyzed soil, vegetables and rice at a 0.05 significance level.^31^ Four certified reference materials GBW10015, NIES-unpolished rice flour high level of Cd, loam soil ERM-CC141 and BCR 701, were evaluated using one-sample *t*-test.

## 3 Results and discussion

### 3.1 Quality control of analytical method

A standard solution was used throughout analysis to calibrate the ICPMS and maintain the accuracy of the analytical method for heavy metal determination in irrigation water, cultivated soils, beetroot, onion, and rice samples. The influence of the sample matrix or variations in the instrument's performance (*e.g.*, sample viscosity and signal drift) was corrected using internal standards In and Ir at 10 µg L^−1^. Four different certified reference materials, namely, GBW10015, NIES-unpolished rice flour with high levels of Cd, loam soil ERM-CC141, and BCR 701, were analysed in order to maintain the validation of the applied analytical method, confirming the reliable detection of Al, Ag, As, Ba, Cd, Co, Cr, Cu, Mn, Mo, Ni, Pb, Fe, Sb, Se, Ti, Tl, V, and Zn. The results of the CRM recoveries, RSD values, calibration coefficients (*R*^2^), procedural blank results, and instrument performance using In and Ir recovery are presented in SI Table 3. The obtained experimental results of Al, Ag, As, Ba, Cd, Co, Cr, Cu, Mn, Mo, Ni, Pb, Fe, Sb, Se, Ti, Tl, V, and Zn for GBW10015, NIES, and loam soil were in good agreement with their certified values for analysed CRMs, with digestion efficiencies documented ranging from 91 to 108%, 89 to 16%, and 91 to 108%, respectively.

### 3.2 Heavy metal concentration in irrigation water

The contents of heavy metals in the irrigation water samples were low compared with those in various parts of vegetables and soil samples. In addition, the rhubarb plant was grown naturally; therefore, it was not possible to collect the water used to grow this plant. Al (except in water used to irrigate rice and onion), Ag, Cd (except in water used for the irrigation of basil), Tl, Ti (in water used to irrigate rice, onion, and basil), and Zn (only in beetroot and radish) were below the LOD in all water samples. Overall, metals such as Al, Ba, Cr, Cu, Fe, Mn, Ti, V, and Zn exist in irrigated water samples used for vegetables and plants in this study at high levels. However, other metals, including As, Co, Mo, Ni, Pb, Sb, and Se, in water samples were present at relatively low levels (Table 1). The values of these metals in irrigation water used for farming vegetable and plants in this study were below the maximum concentration of trace elements in irrigation water recommended by the WHO, as presented in Table 1.

**Table 1:** Concentrations (mean ± SD) of Al, Ag, As, Ba, Cd, Co, Cr, Cu, Fe, Mn, Mo, Ni, Pb, Sb, Se, Ti, Tl, V, and Zn (µg L^−1^) in water samples used to irrigate beetroot, onion, rice, spinach, parsley, dill, basil, and radish^a^

Elements	Beetroot	Rice	Onion	Spinach	Dill	Parsley	Basil	Radish	*P* Value	WHO guidelines for irrigation water^32^
Al	73.4 ± 2 ^a^	<LOD	<LOD	45 ± 3 ^b^	17 ± 0.9 ^c^	47 ± 2 ^b^	1.6 ± 0.4 ^d^	73 ± 2 ^a^	<0.01	500
Ag	<LOD	<LOD	<LOD	<LOD	<LOD	<LOD	<LOD	<LOD	—	NE
As	2.3 ± 0.1 ^a^	0.543 ± 0.05 ^cd^	0.69 ± 0.04 ^bc^	0.53 ± 0.1 ^cd^	0.61 ± 0.01 ^bcd^	0.52 ± 0.1 ^d^	0.7 ± 0.1 ^b^	2.3 ± 0.1 ^a^	<0.01	100
Ba	43 ± 1 ^e^	78.73 ± 2 ^d^	108 ± 5 ^b^	87 ± 2 ^c^	107 ± 2 ^b^	89 ± 2 ^c^	145 ± 5 ^a^	43 ± 1 ^e^	<0.01	NE
Cd	<LOD	<LOD	<LOD	<LOD	<LOD	<LOD	0.05 ± 0.01 ^a^	<LOD	—	10
Co	0.3 ± 0.01 ^b^	<LOD	0.01 ± 0.003^d^	0.44 ± 0.02 ^a^	0.23 ± 0.02 ^c^	0.45 ± 0.003 ^a^	0.01 ± 0.001 ^d^	0.3 ± 0.01 ^b^	<0.01	50
Cr	2.6 ± 0.1 ^e^	1.402 ± 0.1 ^f^	12 ± 0.8 ^a^	3.2 ± 0.1 ^d^	10 ± 0.3 ^b^	3.3 ± 0.2 ^d^	9.1 ± 0.2 ^c^	2.6 ± 0.1 ^e^	<0.01	100
Cu	1.6 ± 0.04 ^b^	0.097 ± 0.01 ^e^	2.3 ± 0.2 ^a^	0.59 ± 0.1 ^d^	1.0 ± 0.1 ^c^	0.67 ± 0.1 ^d^	2.2 ± 0.1 ^a^	1.6 ± 0.04 ^b^	<0.01	200
Fe	58 ± 2 ^c^	5.443 ± 0.5 ^e^	4.8 ± 0.5 ^e^	157 ± 6 ^b^	47 ± 1 ^d^	165 ± 6 ^a^	7.2 ± 0.3 ^e^	58 ± 2 ^c^	<0.01	5000
Mn	0.55 ± 0.04 ^d^	0.103 ± 0.01 ^d^	0.11 ± 0.01 ^d^	52 ± 1 ^b^	40 ± 0.7 ^c^	54 ± 2.0 ^a^	0.8 ± 0.08 ^d^	0.52 ± 0.1 ^d^	<0.01	200
Mo	2.6 ± 0.1 ^a^	1.28 ± 0.04 ^c^	1.0 ± 0.04 ^d^	1.4 ± 0.1 ^b^	1.3 ± 0.01 ^c^	1.5 ± 0.1 ^b^	1.3 ± 0.01 ^c^	2.6 ± 0.1 ^a^	<0.01	10
Ni	2.0 ± 0.03 ^c^	0.102 ± 0.01 ^e^	0.08 ± 0.003 ^e^	2.7 ± 0.05 ^b^	1.6 ± 0.04 ^d^	3 ± 0.2 ^a^	1.6 ± 0.05 ^d^	2.0 ± 0.03 ^c^	<0.01	200
Pb	<LOD	0.094 ± 0.01 ^e^	0.2 ± 0.01 ^d^	0.37 ± 0.01 ^a^	0.27 ± 0.01 ^b^	0.37 ± 0.002 ^a^	0.25 ± 0.01 ^c^	<LOD	<0.01	5000
Sb	0.17 ± 0.01 ^d^	0.007 ± 0.004 ^g^	0.08 ± 0.01 ^e^	0.31 ± 0.03 ^a^	0.23 ± 0.01 ^c^	0.29 ± 0.02 ^b^	0.03 ± 0.004 ^f^	0.17 ± 0.01 ^d^	<0.01	NE
Se	0.89 ± 0.1 ^b^	0.255 ± 9 ^c^	0.22 ± 0.05 ^c^	0.53 ± 0.2 ^bc^	0.52 ± 0.2 ^bc^	0.79 ± 0.2 ^ab^	0.4 ± 0.01 ^c^	0.89 ± 0.1 ^a^	<0.01	20
Ti	2.1 ± 0.1 ^a^	<LOD	<LOD	0.83 ± 0.7 ^bc^	0.09 ± 0.1 ^c^	1.5 ± 0.1 ^ab^	<LOD	2.1 ± 0.7 ^a^	<0.01	NE
Tl	<LOD	<LOD	<LOD	<LOD	<LOD	<LOD	<LOD	<LOD	—	NE
V	17 ± 0.3 ^a^	4.599 ± 0.2 ^c^	8.7 ± 0.5 ^b^	4.5 ± 0.3 ^c^	4.3 ± 0.1 ^c^	4.6 ± 0.2 ^c^	8.5 ± 0.2 ^b^	17 ± 0.2 ^a^	<0.01	100
Zn	<LOD	7.228 ± 0.6 ^d^	126 ± 7 ^a^	39 ± 2 ^c^	38 ± 1 ^c^	41 ± 0.7 ^c^	68 ± 2 ^b^	<LOD	<0.01	2000

a NE: not established. The values reported as < LOD were excluded from statistical analysis. *P* value < 0.05 is considered significant, *P* value < 0.01 is considered highly significant, and the same superscript letters indicate no significant difference. The values with different letters (a, b, c, and d) denote statistically significant differences between groups based on Duncan's multiple range test (*p* < 0.05).

### 3.3 Total heavy metal determination in soil

A pseudo-aqua regia procedure was utilized to measure total metals in soils used to cultivate vegetables and plants in this study; the results are depicted in Table 2. There are distinct differences between the contents of trace metals in the examined soils used for the cultivation of vegetables in this study. High contents of Al (6213–12890 mg kg^−1^), As (1.6–5.8 mg kg^−1^), Ba (58–127 mg kg^−1^), Co (10.8–13.8 mg kg^−1^), Cr (62–164 mg kg^−1^), Cu (20–42.9 mg kg^−1^), Fe (12 840–17150 mg kg^−1^), Mn (223–427 mg kg^−1^), Ni (81–214 mg kg^−1^), Pb (7.8–21 mg kg^−1^), Ti (458–806 mg kg^−1^), V (40–51.4 mg kg^−1^), and Zn (43–97 mg kg^−1^) were detected in analysed soils used for the cultivation of different vegetables. However, relatively low concentrations of Ag (0.04–0.38 mg kg^−1^), Cd (0.12–0.48 mg kg^−1^), Mo (0.34–0.69 mg kg^−1^), Sb (0.126–0.25 mg kg^−1^), Se (0.156–0.355 mg kg^−1^), and Tl (0.064–4.4 mg kg^−1^) were found in the analysed soil samples in this study.

**Table 2:** Concentrations (mean ± SD) of Al, As, B, Ba, V, Ti, Cr, Mn, Mo, Fe, Co, Ni, Cu, Zn, Se, Sb, and Pb (mg kg^−1^) in soil samples used to cultivate beetroot, onion, rice, spinach, parsley, dill, rhubarb, basil, and radish^a^

Metal	BCR701	Beetroot	Rice	Onion	Spinach	Dill	Parsley	Rhubarb	Basil	Radish	*P* Value
Al	6798 ± 68.9	9561 ± 68 ^f^	10 970 ± 51 ^b^	9598 ± 48 ^f^	10 260 ± 46 ^e^	9279 ± 56 ^g^	10 620 ± 123 ^c^	12 890 ± 37 ^a^	6213 ± 19 ^h^	10 490 ± 106 ^d^	<0.01
Ag	5.547 ± 0.05	0.05 ± 0.003 ^f^	0.087 ± 0.001 ^d^	0.040 ± 0.003 ^g^	0.29 ± 0.01 ^b^	0.38 ± 0.01 ^a^	0.21 ± 0.003 ^c^	0.08 ± 0.001 ^e^	0.052 ± 0.001 ^f^	0.074 ± 0.003 ^e^	<0.01
As	21.7 ± 0.17	5.6 ± 0.1 ^b^	3.52 ± 0.01 ^g^	1.6 ± 0.04 ^h^	5.2 ± 0.1 ^d^	5 ± 0.1 ^e^	5.4 ± 0.1 ^c^	5.6 ± 0.05 ^b^	3.9 ± 0.1 ^f^	5.8 ± 0.1 ^a^	<0.01
Ba	98 ± 0.5	87 ± 0.3 ^e^	127 ± 0.4 ^a^	104 ± 0.7 ^c^	83 ± 0.5 ^f^	73 ± 0.6 ^g^	89 ± 0.8 ^d^	89 ± 0.6 ^d^	58 ± 0.6 ^h^	117 ± 1 ^b^	<0.01
Cd	12.89 ± 0.08	0.40 ± 0.01 ^b^	0.42 ± 0.02 ^b^	0.48 ± 0.01 ^a^	0.33 ± 0.02 ^c^	0.26 ± 0.004 ^d^	0.24 ± 0.01 ^d^	0.24 ± 0.01 ^d^	0.12 ± 0.01 ^e^	0.25 ± 0.01 ^d^	<0.01
Co	7.68 ± 0.07	11.2 ± 0.1 ^g^	13.8 ± 0.2 ^a^	11.5 ± 0.2 ^f^	11.9 ± 0.02 ^d^	10.8 ± 0.04 ^h^	12.4 ± 0.1 ^c^	13.2 ± 0.05 ^b^	11.8 ± 0.01 ^e^	12.3 ± 0.1 ^c^	<0.01
Cr	226.6 ± 1.165	105.7 ± 0.5 ^b^	164 ± 0.8 ^a^	106.3 ± 0.4 ^b^	68 ± 0.3 ^g^	62 ± 0.3 ^h^	71.2 ± 0.6 ^f^	94 ± 0.3 ^d^	102 ± 0.2 ^c^	72.2 ± 1 ^e^	<0.01
Cu	253 ± 0.936	42.9 ± 0.04 ^a^	41 ± 0.3 ^b^	38 ± 0.2 ^c^	25.7 ± 0.1 ^e^	33 ± 0.2 ^d^	25.4 ± 0.2 ^e^	24.5 ± 0.2 ^f^	21 ± 0.03 ^g^	20 ± 0.2 ^h^	<0.01
Fe	12 060 ± 59	14 290 ± 90 ^f^	16 230 ± 116 ^b^	14 470 ± 97 ^e^	14 660 ± 16 ^d^	13 320 ± 60 ^g^	15 330 ± 164 ^c^	17 150 ± 53 ^a^	12 840 ± 90 ^h^	15 200 ± 151 ^c^	<0.01
Mn	215.7 ± 0.9	316 ± 2.4 ^e^	223 ± 2 ^h^	309.6 ± 2 ^f^	341 ± 2 ^c^	308.7 ± 2 ^f^	369 ± 4 ^b^	427 ± 2 ^a^	263 ± 2 ^g^	328 ± 4 ^d^	<0.01
Mo	1.779 ± 0.04	0.509 ± 0.01 ^d^	0.66 ± 0.01 ^b^	0.527 ± 0.01 ^d^	0.34 ± 0.01 ^g^	0.365 ± 0.01 ^f^	0.35 ± 0.02 ^fg^	0.69 ± 0.03 ^a^	0.57 ± 0.01 ^c^	0.43 ± 0.01 ^e^	<0.01
Ni	89.3 ± 0.6	139 ± 0.7 ^c^	214 ± 1 ^a^	141 ± 1 ^b^	89 ± 0.5 ^f^	81 ± 0.4 ^g^	92.9 ± 0.7 ^e^	93 ± 0.3 ^e^	113 ± 0.2 ^d^	88 ± 0.9 ^f^	<0.01
Pb	130.3 ± 11.2	13.3 ± 0.05 ^d^	18.3 ± 0.1 ^b^	21 ± 0.3 ^a^	13.4 ± 0.1 ^d^	18.1 ± 0.05 ^c^	13.2 ± 0.1 ^d^	11.4 ± 0.1 ^e^	7.8 ± 0.03 ^g^	8.7 ± 0.1 ^f^	<0.01
Sb	1.07 ± 0.02	0.18 ± 0.003 ^d^	0.126 ± 0.01 ^h^	0.134 ± 0.002 ^g^	0.15 ± 0.01 ^f^	0.25 ± 0.001 ^a^	0.136 ± 0.003 ^g^	0.21 ± 0.01 ^c^	0.22 ± 0.004 ^b^	0.16 ± 0.001 ^e^	<0.01
Se	0.51 ± 0.01	0.21 ± 0.02 ^c^	0.355 ± 0.03 ^a^	0.17 ± 0.01 ^de^	0.18 ± 0.02 ^cde^	0.19 ± 0.01 ^cd^	0.20 ± 0.01 ^c^	0.25 ± 0.01 ^b^	0.156 ± 0.01 ^e^	0.158 ± 0.01 ^e^	<0.01
Ti	663.5 ± 4.6	623 ± 2.7 ^d^	458 ± 2 ^h^	755 ± 4 ^b^	541 ± 3 ^f^	531 ± 3 ^g^	539 ± 5 ^f^	669 ± 2 ^c^	806 ± 2 ^a^	557 ± 6 ^e^	<0.01
Tl	0.231 ± 0.002	0.102 ± 0.002^de^	0.176 ± 0.002 ^bc^	0.13 ± 0.002 ^cd^	0.11 ± 0.003 ^de^	0.09 ± 0.001 ^de^	0.117 ± 0.001 ^cde^	4.4 ± 0.1 ^a^	0.064 ± 0.002 ^e^	0.19 ± 0.01 ^b^	<0.01
V	27.4 ± 0.3	49.4 ± 0.2 ^c^	49.8 ± 0.4 ^c^	51.4 ± 0.2 ^a^	43.8 ± 0.003 ^e^	40 ± 0.2 ^f^	45.4 ± 0.4 ^d^	50.7 ± 0.1 ^b^	43.5 ± 0.3 ^e^	51.3 ± 0.8 ^ab^	<0.01
Zn	446.9 ± 6.3	68 ± 0.2 ^c^	73 ± 0.7 ^b^	97 ± 0.8 ^a^	59 ± 0.2 ^f^	67 ± 0.2 ^d^	61 ± 0.7 ^e^	50 ± 0.1 ^g^	43 ± 0.1^i^	45 ± 0.35^h^	<0.01

a NE: not established. The values reported as < LOD were excluded from statistical analysis. *P* value < 0.05 is considered significant, *P* value < 0.01 is considered highly significant, and the same superscript letters indicate no significant difference. The values with different letters (a, b, c, d) denote statistically significant differences between groups based on Duncan's multiple range test (*p* < 0.05).

Although the concentration of Cu in the cultivated soil in this study was lower than that reported in the literature, the contents of Cr, Fe, Ni, Zn, Cd, and Pb were higher than the reported values. In addition, the content of Mn in the soil in this study is consistent with that reported in other studies.^33^ The As content in the soil in this study was consistent with the reported values, which ranged from 2.98 to 3.76 mg kg^−1^, while the Co content was lower than that reported in other studies, where it ranged from 19.56 to 43.09 mg kg^−1^.

### 3.4 Heavy metal concentrations in different parts of beetroot, onion, rice, spinach, dill, parsley rhubarb, basil, and radish

#### 3.4.1 Beetroot

The average of standard deviations and contents of Al, Ag, As, Ba, Cd, Co, Cr, Cu, Mn, Mo, Ni, Pb, Fe, Sb, Se, Ti, Tl, V, and Zn in the different parts of beetroot samples determined on the basis of a dry mass scale are depicted in Table 3. Among the 19 metals studied in beetroot, their distribution patterns within the plant varied; only Al, Fe, and V shared a similar distribution pattern, as did Ti and Pb, while the others were distributed differently across the roots, skin, stems, and leaves. The concentrations of “plant-available” fraction were substantially different among the analysed metals in this study (Table 4). Mn demonstrated the highest levels of available fraction (68 mg kg^−1^), equivalent to 22% of the total aqua regia content of the soil used to cultivate beetroot. However, Al, Ag, Pb, Sb, and Tl were below the LOD. Ba exhibited a relatively high plant-available fraction (23 mg kg^−1^), comprising approximately 23% of the pseudo-total aqua regia metal content of the soil used to cultivate beetroot, followed by Ni (1.32 mg kg; corresponding to 0.9%). The As plant-available fraction comprised 3% (0.17 mg kg^−1^) of the total aqua regia metal content of the soil. In comparison, Cd, Co, Cr, and Cu represented 18% (0.07 mg kg^−1^), 1.8% (0.2 mg kg^−1^), 0.19% (0.2 mg kg^−1^) and 0.19% (0.08 mg kg^−1^) of the total aqua regia content, respectively. The distribution of metals across organs did not follow the same pattern. For Se, Mo, Cd, and Tl, the roots accumulated the highest contents of these metals. However, the skin of beetroot accumulated the highest contents of Al, As, V, Cr, Fe, Co, and Cu. This suggests that the skin acts as a barrier for the other organs. Therefore, this compartment is not recommended for human consumption. Although the stem had the highest content of Ni, the leaves accumulated the highest contents of Ba, Ti, Mn, and Pb. It is interesting to note that, although the plant-available contents of Al, Ti, and Fe were below the LOD and accounted for only 0.01% of their total aqua regia contents, the beetroot plant accumulated a substantial quantity of these metals. This finding indicates the capability of this plant to pre-concentrate these metals across organs.

**Table 3:** Concentrations of Al, Ag, As, Ba, Cd, Co, Cr, Cu, Mn, Mo, Ni, Pb, Fe, Sb, Se, Ti, Tl, V, and Zn (mg kg^−1^) on dry mass scale in beetroot, onion, rice, spinach, dill, parsley, rhubarb, basil, and radish plants determined using ICP-MS^a^

Sample	Al	Ag	As	Ba	V	Ti	Cr	Mn	Fe	Co	Ni	Cu	Zn	Se	Mo	Cd	Sb	Tl	Pb
Beetroot-root	655 ± 7 ^c^	0.331 ± 0.01 ^b^	0.221 ± 0.01 ^c^	4.29 ± 0.1 ^d^	1.94 ± 0.02 ^c^	22.4 ± 1^d^	0.249 ± 0.006 ^c^	7.78 ± 0.1^d^	81 ± 1 ^c^	0.069 ± 0.001 ^d^	0.019 ± 0.0003 ^b^	0.898 ± 0.02 ^c^	3.5 ± 0.043 ^b^	0.104 ± 0.017 ^a^	0.156 ± 0.01 ^a^	0.011 ± 0.0001 ^b^	<LOD	0.014 ± 0.001	0.22 ± 0.003 ^c^
Beetroot-skin	1205 ± 17 ^a^	0.12 ± 0.003 ^d^	0.347 ± 0.02 ^a^	4.26 ± 0.1 ^d^	3.88 ± 0.1 ^a^	35.03 ± 0.3 ^b^	0.399 ± 0.002 ^a^	12 ± 0.2 ^b^	154 ± 1.4^a^	0.117 ± 0.001 ^a^	0.042 ± 0.001 ^b^	1.675 ± 0.03^a^	3.34 ± 0.06 ^c^	0.034 ± 0.01 ^b^	0.021 ± 0.001 ^c^	0.010 ± 0.0002 ^c^	<LOD	<LOD	0.27 ± 0.001 ^b^
Beetroot-core	436 ± 5 ^e^	0.341 ± 0.002 ^a^	0.182 ± 0.02 ^d^	7.05 ± 0.1 ^b^	1.15 ± 0.02 ^e^	24.7 ± 1.2 ^c^	0.195 ± 0.006 ^d^	10.8 ± 0.1^c^	54 ± 1 ^e^	0.080 ± 0.002 ^c^	0.022 ± 0.0002 ^b^	1.588 ± 0.04 ^b^	4.85 ± 0.05 ^a^	0.011 ± 0.001 ^c^	0.020 ± 0.001 ^c^	0.011 ± 0.0005 ^b^	<LOD	<LOD	0.227 ± 0.004 ^c^
Beetroot-stem	601 ± 6 ^d^	0.162 ± 0.002 ^c^	0.3 ± 0.02 ^b^	4.59 ± 0.1 ^c^	1.56 ± 0.01 ^d^	19.3 ± 0.8 ^e^	0.25 ± 0.002 ^c^	7.99 ± 0.1^d^	77 ± 0.6 ^d^	0.067 ± 0.001 ^e^	3.112 ± 0.1 ^a^	0.056 ± 0.001 ^d^	2.24 ± 0.1^d^	0.007 ± 0.002 ^c^	0.015 ± 0.000 ^c^	<LOD	<LOD	<LOD	0.19 ± 0.004 ^d^
Beetroot-leaf	1176 ± 16 ^b^	0.048 ± 0.002 ^e^	0.203 ± 0.01 ^cd^	7.77 ± 0.1^a^	2.67 ± 0.12^b^	47.6 ± 1 ^a^	0.345 ± 0.011 ^b^	41.9 ± 1.3^a^	137 ± 3^b^	0.089 ± 0.001 ^b^	0.025 ± 0.0003 ^b^	1.533 ± 0.05 ^b^	3.28 ± 0.106 ^c^	0.015 ± 0.001 ^c^	0.041 ± 0.001 ^b^	0.013 ± 0.0002 ^a^	<LOD	<LOD	0.395 ± 0.004 ^a^
*P* Value	<0.01	<0.01	<0.01	<0.01	<0.01	<0.01	<0.01	<0.01	<0.01	<0.01	<0.01	<0.01	<0.01	<0.01	<0.01	<0.01	—	—	<0.01
Onion -root	130 ± 1^d^	<LOD	2.06 ± 0.01 ^a^	1.88 ± 0.1 ^d^	3.34 ± 0.1 ^c^	48.4 ± 1 ^b^	9.58 ± 0.2 ^c^	39.1 ± 1 ^c^	779 ± 13 ^b^	1.59 ± 0.03 ^b^	9.35 ± 0.2 ^c^	6.8 ± 0.2 ^c^	27.9 ± 0.6 ^a^	1.56 ± 0.1 ^a^	1.18 ± 0.02 ^b^	0.13 ± 0.003 ^a^	0.03 ± 0.0003 ^b^	<LOD	0.61 ± 0.01 ^b^
Onion-skin	210 ± 2 ^b^	0.036 ± 0.004 ^a^	0.151 ± 0.01 ^c^	14.7 ± 0.1 ^b^	0.51 ± 0.01 ^d^	30.7 ± 1 ^c^	1.88 ± 0.04 ^d^	28.5 ± 0.2^d^	149 ± 2 ^d^	0.199 ± 0.01 ^d^	2.62 ± 0.002 ^d^	5.9 ± 0.1 ^d^	21.2 ± 0.2^d^	0.19 ± 0.01 ^d^	0.475 ± 0.01 ^c^	0.049 ± 0.003 ^ab^	0.05 ± 0.008 ^a^	0.02 ± 0.002	0.33 ± 0.005 ^d^
Onion-bulb	199 ± 3 ^c^	0.023 ± 0.002 ^b^	0.148 ± 0.01 ^c^	14.8 ± 0.1 ^b^	0.52 ± 0.08 ^d^	28.2 ± 1 ^d^	1.74 ± 0.02 ^d^	28.2 ± 0.2^d^	143 ± 2 ^d^	0.180 ± 0.004 ^d^	2.53 ± 0.01 ^d^	6 ± 0.1 ^d^	21.7 ± 0.1^d^	0.273 ± 0.01 ^c^	0.39 ± 0.002 ^d^	0.042 ± 0.003 ^b^	<LOD	<LOD	0.319 ± 0.004 ^d^
Onion-stem	381 ± 4 ^a^	0.027 ± 0.001 ^b^	0.693 ± 0.01 ^b^	8.77 ± 1 ^c^	6.63 ± 0.02 ^a^	16.8 ± 0.1 ^e^	25.5 ± 0.1 ^b^	73.7 ± 0.3^b^	2605 ± 4 ^b^	2.69 ± 0.02 ^a^	63.9 ± 0.9 ^a^	12.2 ± 0.1 ^a^	26 ± 0.2 ^b^	0.95 ± 0.1 ^b^	2.36 ± 0.1 ^a^	0.056 ± 0.003 ^b^	0.047 ± 0.002 ^a^	<LOD	0.443 ± 0.01 ^c^
Onion -leaf	378 ± 7 ^a^	<LOD	0.694 ± 0.02 ^b^	30.67 ± 1^a^	6.33 ± 0.1 ^b^	142 ± 2 ^a^	85.7 ± 1 ^a^	139 ± 1.5^a^	3502 ± 39 ^a^	1.45 ± 0.01 ^c^	23.1 ± 0.1 ^b^	7.21 ± 0.1 ^b^	22 ± 0.3 ^c^	0.052 ± 0.001 ^e^	1.16 ± 0.01 ^b^	0.056 ± 0.01 ^b^	<LOD	<LOD	1.432 ± 0.01 ^a^
*P* Value	<0.01	0.02	<0.01	<0.01	<0.01	<0.01	<0.01	<0.01	<0.01	<0.01	<0.01	<0.01	<0.01	<0.01	<0.01	<0.01	<0.01	—	<0.01
Rice-root	1520 ± 23 ^a^	0.29 ± 0.001 ^a^	8.4 ± 0.1 ^a^	3.2 ± 0.1 ^b^	8.32 ± 0.15 ^a^	69 ± 4 ^a^	3.7 ± 0.04 ^c^	39 ± 0.5 ^c^	988 ± 9 ^a^	0.29 ± 0.002 ^b^	0.11 ± 0.003 ^b^	0.84 ± 0.01 ^b^	1.6 ± 0.01^b^	0.04 ± 0.002 ^a^	0.12 ± 0.003 ^c^	0.008 ± 0.0003	<LOD	<LOD	2.1 ± 0.03 ^a^
Rice-stem	771 ± 13 ^b^	0.03 ± 0.002 ^c^	0.35 ± 0.02 ^d^	2.4 ± 0.1 ^c^	2.4 ± 0.07 ^b^	37 ± 2 ^b^	4.4 ± 0.1 ^b^	149 ± 4 ^b^	165 ± 4 ^c^	0.11 ± 0.002 ^c^	0.09 ± 0.002 ^b^	0.34 ± 0.01 ^c^	0.9 ± 0.03^c^	0.017 ± 0.001 ^b^	0.09 ± 0.002 ^d^	<LOD	<LOD	<LOD	0.74 ± 0.01 ^b^
Rice-leaf	107 ± 1 ^c^	0.06 ± 0.002 ^b^	3 ± 0.1 ^b^	15 ± 0.2 ^a^	0.35 ± 0.02 ^c^	5.9 ± 0.2 ^c^	20 ± 0.4 ^a^	584 ± 8 ^a^	461 ± 19 ^b^	0.36 ± 0.01 ^a^	3 ± 0.3 ^a^	2.6 ± 0.03 ^a^	18 ± 0.6 ^a^	0.018 ± 0.004 ^b^	0.31 ± 0.004 ^a^	0.009 ± 0.001	<LOD	<LOD	0.4 ± 0.001 ^c^
Rice-grain	99 ± 3 ^c^	<LOD	1 ± 0.02 ^c^	0.2 ± 0.01 ^d^	0.40 ± 0.02 ^c^	4.9 ± 0.2 ^c^	3 ± 0.03 ^d^	31 ± 1 ^c^	43 ± 2 ^d^	0.03 ± 0.002 ^d^	0.04 ± 0.002 ^b^	0.3 ± 0.01 ^d^	1.7 ± 0.04^b^	0.013 ± 0.002 ^b^	0.27 ± 0.01 ^b^	<LOD	<LOD	<LOD	0.26 ± 0.006 ^d^
*P* Value	<0.01	<0.01	<0.01	<0.01	<0.01	<0.01	<0.01	<0.01	<0.01	<0.01	<0.01	<0.01	<0.01	<0.01	<0.01	—	—	—	<0.01
Spinach-root	298 ± 2 ^c^	0.05 ± 0.004 ^a^	0.26 ± 0.02 ^a^	19 ± 0.2 ^c^	1.0 ± 0.01 ^c^	18 ± 0.3 ^b^	5.5 ± 0.1 ^b^	22 ± 0.2 ^c^	470 ± 4 ^c^	0.38 ± 0.03 ^c^	2.8 ± 0.1 ^c^	7.0 ± 0.1 ^c^	34 ± 0.3 ^c^	0.13 ± 0.01 ^b^	0.29 ± 0.002 ^b^	0.16 ± 0.001 ^b^	<LOD	<LOD	0.23 ± 0.001 ^c^
Spinach-stem	333 ± 3 ^b^	0.05 ± 0.001 ^a^	0.26 ± 0.002 ^a^	20 ± 0.09^b^	1.2 ± 0.01 ^b^	15 ± 0.07 ^c^	5.8 ± 0.03 ^a^	25 ± 0.04^b^	532 ± 2 ^b^	0.43 ± 0.002 ^b^	3.1 ± 0.02 ^a^	7.9 ± 0.02 ^b^	38 ± 0.2 ^b^	0.26 ± 0.03 ^a^	0.30 ± 0.003 ^b^	0.17 ± 0.01^b^	0.01 ± 0.001	<LOD	0.26 ± 0.005 ^b^
Spinach-leaf	1053 ± 4 ^a^	0.02 ± 0.002 ^b^	0.18 ± 0.01 ^b^	27 ± 0.1 ^a^	1.5 ± 0.01 ^a^	30 ± 1 ^a^	2.8 ± 0.01 ^c^	65 ± 0.5 ^a^	1014 ± 7 ^a^	0.44 ± 0.003 ^a^	2.9 ± 0.02 ^b^	8.5 ± 0.03 ^a^	47 ± 0.4 ^a^	0.22 ± 0.02 ^a^	0.34 ± 0.01 ^a^	0.24 ± 0.01 ^a^	0.02 ± 0.001	<LOD	0.28 ± 0.004 ^a^
*P* Value	<0.01	<0.01	<0.01	<0.01	<0.01	<0.01	<0.01	<0.01	<0.01	<0.01	<0.01	<0.01	<0.01	<0.01**	<0.01	<0.01	—	—	<0.01
Dill-root	424 ± 23 ^a^	0.02 ± 0.001	0.37 ± 0.01 ^a^	20 ± 0.1 ^b^	1.3 ± 0.02 ^a^	23 ± 2 ^a^	3.7 ± 0.04 ^b^	24 ± 0.1 ^c^	671 ± 4 ^b^	0.32 ± 0.001 ^a^	5.2 ± 0.01 ^a^	4.6 ± 0.03 ^a^	51 ± 0.4 ^a^	0.12 ± 0.01 ^b^	0.78 ± 0.01 ^c^	0.24 ± 0.004 ^a^	0.02 ± 0.001 ^a^	<LOD	0.28 ± 0.001 ^b^
Dill-stem	342 ± 2 ^b^	<LOD	0.28 ± 0.01^b^	23 ± 0.06^a^	0.96 ± 0.02 ^c^	23 ± 0.9 ^a^	4.5 ± 0.01 ^a^	29 ± 0.2 ^b^	564 ± 3 ^c^	0.25 ± 0.003 ^b^	4.3 ± 0.04 ^b^	3.5 ± 0.02 ^b^	39 ± 0.4 ^c^	0.15 ± 0.01 ^a^	1.2 ± 0.01 ^b^	0.23 ± 0.01 ^ab^	0.01 ± 0.001 ^c^	<LOD	0.20 ± 0.003 ^c^
Dill-leaf	398 ± 11 ^a^	<LOD	0.21 ± 0.01 ^c^	15 ± 0.2 ^c^	1.1 ± 0.02 ^b^	24 ± 3 ^a^	1.9 ± 0.02 ^c^	61 ± 0.7 ^a^	733 ± 7 ^a^	0.31 ± 0.01 ^a^	4.2 ± 0.04 ^c^	4.6 ± 0.02 ^a^	46 ± 0.1 ^b^	0.10 ± 0.008 ^b^	1.6 ± 0.04 ^a^	0.22 ± 0.004 ^b^	0.014 ± 0.001 ^b^	0.029 ± 0.001	0.38 ± 0.002 ^a^
*P* Value	<0.01**	—	<0.01	<0.01	<0.01	0.532	<0.01	<0.01	<0.01	<0.01	<0.01	<0.01	<0.01	<0.01	<0.01	0.012	<0.01	—	<0.01
Parsley -root	1477 ± 22 ^a^	<LOD	0.32 ± 0.003 ^a^	36 ± 0.2 ^a^	3.8 ± 0.02 ^a^	38 ± 2 ^b^	11 ± 0.1 ^a^	69 ± 0.8 ^c^	1284 ± 17 ^a^	0.65 ± 0.01 ^a^	6.8 ± 0.1 ^a^	14 ± 0.1 ^a^	34 ± 0.3^a^	0.093 ± 0.01^a^	0.20 ± 0.001^c^	0.30 ± 0.004^a^	0.022 ± 0.001^a^	<LOD	0.50 ± 0.003 ^b^
Parsley -stem	1113 ± 11 ^b^	<LOD	0.21 ± 0.1 ^b^	27 ± 0.1 ^c^	2.0 ± 0.02 ^b^	57 ± 2 ^a^	5.8 ± 0.04 ^b^	85 ± 1 ^b^	998 ± 15 ^b^	0.63 ± 0.01 ^a^	5.3 ± 0.1 ^b^	9.1 ± 0.1 ^b^	31 ± 0.4^c^	0.08 ± 0.04^a^	0.37 ± 0.01^b^	0.20 ± 0.01^b^	0.02 ± 0.002^a^	<LOD	1.8 ± 0.03 ^a^
Parsley -leaf	380 ± 13 ^c^	<LOD	0.11 ± 0.01 ^c^	28 ± 0.1 ^b^	0.99 ± 0.004 ^c^	24 ± 1 ^c^	1.8 ± 0.02 ^c^	108 ± 0.1^a^	620 ± 2 ^c^	0.27 ± 0.02 ^b^	4.5 ± 0.1 ^c^	9.2 ± 0.04 ^b^	32 ± 0.2^b^	0.089 ± 0.01^a^	1.0 ± 0.01^a^	0.11 ± 0.002^c^	0.012 ± 0.001^b^	<LOD	0.28 ± 0.001 ^c^
*P* Value	<0.01	—	<0.01	<0.01	<0.01	<0.01	<0.01	<0.01	<0.01	<0.01	<0.01	<0.01	<0.01	0.726	<0.01	<0.01	<0.01	—	<0.01
Rhubarb-root	76 ± 0.4 ^a^	<LOD	0.029 ± 0.001 ^a^	9.5 ± 0.08 ^a^	0.15 ± 0.01 ^a^	3.3 ± 0.1 ^a^	0.42 ± 0.004 ^a^	15 ± 0.2 ^ab^	68 ± 0.4 ^b^	0.065 ± 0.001 ^a^	1.3 ± 0.02 ^b^	1.9 ± 0.03 ^b^	3.8 ± 0.04^c^	0.029 ± 0.001 ^a^	0.017 ± 0.001 ^b^	0.011 ± 0.001 ^b^	<LOD	<LOD	0.17 ± 0.002 ^b^
Rhubarb-skin	71 ± 1 ^b^	<LOD	0.009 ± 0.002 ^c^	9.6 ± 0.1 ^a^	0.14 ± 0.004 ^a^	3.0 ± 0.1 ^bc^	0.397 ± 0.01 ^c^	14 ± 0.1 ^b^	67 ± 0.5 ^bc^	0.065 ± 0.002 ^a^	1.3 ± 0.004 ^b^	1.9 ± 0.03 ^b^	3.8 ± 0.03^c^	0.029 ± 0.001 ^a^	0.017 ± 0.001 ^b^	0.012 ± 0.001 ^b^	<LOD	<LOD	0.134 ± 0.002 ^c^
Rhubarb-stem	74 ± 1 ^a^	<LOD	0.014 ± 0.001 ^b^	9.5 ± 0.04 ^a^	0.143 ± 0.003 ^a^	3.1 ± 0.1 ^ab^	0.409 ± 0.002 ^b^	15 ± 0.1 ^a^	69 ± 0.7 ^a^	0.067 ± 0.004 ^a^	1.4 ± 0.01 ^a^	2.0 ± 0.02 ^a^	4.2 ± 0.1 ^b^	0.014 ± 0.001 ^b^	0.018 ± 0.001 ^ab^	0.013 ± 0.001 ^a^	<LOD	<LOD	0.135 ± 0.002 ^c^
Rhubarb-leaf	69 ± 0.6 ^c^	<LOD	0.014 ± 0.001 ^b^	8.7 ± 0.1^b^	0.135 ± 0.01 ^a^	2.9 ± 0.1 ^c^	0.377 ± 0.003 ^d^	13 ± 0.2 ^c^	66 ± 0.4 ^c^	0.061 ± 0.001 ^b^	1.2 ± 0.01 ^c^	1.8 ± 0.02 ^c^	6.7 ± 0.004 ^a^	0.011 ± 0.002 ^c^	0.020 ± 0.002 ^a^	0.013 ± 0.001 ^a^	<LOD	<LOD	0.209 ± 0.001 ^a^
*P* Value	—	—	<0.01	<0.01	0.186	<0.01	<0.01	<0.01	0.01	0.046	<0.01	<0.01	<0.01	<0.01	0.063	0.03	—	—	<0.01
Basil-root	1695 ± 24 ^a^	<LOD	0.858 ± 0.03 ^a^	81 ± 0.5 ^a^	9.7 ± 0.2 ^a^	91 ± 2 ^a^	18 ± 0.3 ^a^	80 ± 1 ^a^	2656 ± 45 ^a^	1.7 ± 0.02 ^a^	12 ± 0.2 ^a^	14 ± 0.1 ^a^	42 ± 0.2 ^a^	0.102 ± 0.004 ^a^	0.654 ± 0.01 ^a^	0.533 ± 0.02 ^a^	0.173 ± 0.02 ^a^	<LOD	0.841 ± 0.01 ^a^
Basil-stem	610 ± 7 ^b^	<LOD	0.193 ± 0.01 ^c^	65 ± 0.4 ^b^	2.5 ± 0.05 ^b^	32 ± 2 ^b^	5.6 ± 0.05 ^b^	37 ± 0.6 ^c^	1068 ± 19 ^c^	0.51 ± 0.01 ^b^	4.2 ± 0.04 ^c^	7.7 ± 0.1 ^c^	25 ± 0.3 ^c^	0.022 ± 0.003 ^c^	0.236 ± 0.003 ^c^	0.011 ± 0.001 ^b^	0.045 ± 0.002 ^b^	<LOD	0.388 ± 0.01 ^c^
Basil-leaf	624 ± 6 ^b^	<LOD	0.237 ± 0.001 ^b^	41 ± 0.5 ^c^	1.8 ± 0.02 ^c^	34 ± 2 ^b^	4.5 ± 0.1 ^c^	51 ± 0.7 ^b^	1148 ± 27 ^b^	0.43 ± 0.01 ^c^	4.5 ± 0.05 ^b^	8.0 ± 0.1 ^b^	29 ± 0.4 ^b^	0.048 ± 0.01 ^b^	0.452 ± 0.01 ^b^	0.008 ± 0.001 ^b^	0.044 ± 0.003 ^b^	<LOD	0.461 ± 0.01 ^b^
*P* Value	<0.01	—	<0.01	<0.01	<0.01	<0.01	<0.01	<0.01	<0.01	<0.01	<0.01	<0.01	<0.01	<0.01	<0.01	<0.01	<0.01	—	<0.01
Radish-root	393 ± 6 ^d^	0.071 ± 0.003 ^b^	0.678 ± 0.02 ^b^	35 ± 0.7 ^b^	1.9 ± 0.03 ^b^	15 ± 0.01 ^cd^	2.8 ± 0.01 ^c^	26 ± 0.4 ^d^	562 ± 8 ^d^	0.793 ± 0.01 ^b^	4.6 ± 0.05 ^c^	3.6 ± 0.1 ^b^	20 ± 0.5 ^a^	0.181 ± 0.03 ^a^	2.2 ± 0.01 ^b^	0.151 ± 0.003 ^b^	0.086 ± 0.01 ^a^	0.035 ± 0.003 ^b^	0.143 ± 0.003 ^d^
Radish-skin	510 ± 5 ^c^	0.016 ± 0.001 ^c^	0.404 ± 0.01 ^c^	36 ± 0.2 ^b^	1.8 ± 0.04 ^c^	16 ± 0.7 ^c^	2.8 ± 0.03 ^c^	32 ± 0.5 ^c^	737 ± 13 ^c^	0.868 ± 0.03 ^a^	6.4 ± 0.1 ^a^	3.0 ± 0.1 ^d^	15 ± 0.3 ^d^	0.142 ± 0.01 ^b^	0.937 ± 0.02 ^c^	0.118 ± 0.01 ^c^	0.068 ± 0.003 ^b^	0.013 ± 0.001 ^c^	0.139 ± 0.01 ^d^
Radish-core	384 ± 3 ^d^	0.147 ± 0.01 ^a^	0.746 ± 0.04 ^a^	35 ± 0.1 ^b^	2.0 ± 0.02 ^a^	15 ± 0.9 ^d^	2.8 ± 0.01 ^c^	26 ± 0.3 ^d^	553 ± 8 ^d^	0.841 ± 0.02 ^a^	4.5 ± 0.05 ^c^	3.4 ± 0.02 ^c^	19 ± 0.3 ^b^	0.192 ± 0.01 ^a^	2.7 ± 0.2 ^a^	0.2 ± 0.01 ^a^	0.048 ± 0.003 ^c^	0.081 ± 0.01^a^	0.179 ± 0.01 ^b^
Radish-stem	672 ± 2 ^b^	<LOD	0.312 ± 0.01 ^d^	52 ± 0.5 ^a^	1.8 ± 0.03 ^d^	28 ± 0.6 ^a^	6.1 ± 0.1 ^a^	51 ± 1 ^a^	972 ± 18 ^a^	0.687 ± 0.01 ^c^	5.6 ± 0.1 ^b^	2.8 ± 0.05 ^d^	16 ± 0.1 ^c^	0.152 ± 0.004 ^b^	0.855 ± 0.01 ^c^	0.123 ± 0.01 ^c^	0.021 ± 0.001 ^d^	<LOD	0.166 ± 0.003 ^c^
Radish-leaf	949 ± 19 ^a^	<LOD	0.162 ± 0.01 ^e^	16 ± 0.5 ^c^	1.9 ± 0.03 ^c^	25 ± 1 ^b^	5.2 ± 0.2 ^b^	37 ± 0.5 ^b^	842 ± 15 ^b^	0.460 ± 0.02 ^d^	4.3 ± 0.1 ^d^	4.5 ± 0.1 ^a^	14 ± 0.5 ^e^	0.02 ± 0.0004 ^c^	0.583 ± 0.004 ^d^	0.081 ± 0.001 ^d^	0.01 ± 0.001 ^e^	<LOD	0.764 ± 0.01 ^a^
*P* Value	<0.01	<0.01	<0.01	<0.01	<0.01	<0.01	<0.01	<0.01	<0.01	<0.01	<0.01	<0.01	<0.01	<0.01	<0.01	<0.01	<0.01	<0.01	<0.01
WHO/FAO (ML)	NE	NE	NE	NE	NE	NE	NE	NE	NE	NE	0.9^a^	40	60	NE	NE	0.3	NE	NE	0.3
Reference											56	57	58			59			59

a ML: maximum limit. The values reported as < LOD were excluded from statistical analysis. *P* value < 0.05 is considered significant, *P* value < 0.01 is considered highly significant, and the same superscript letters indicate no significant difference. The values with different letters (a, b, c, and d) denote statistically significant differences between groups based on Duncan's multiple range test (*p* < 0.05). ^a^European Commission; BMDL0.5: Benchmark Dose Lower Confidence Limit for 0.5.

**Table 4:** Results of the analysis of BCR 701 and soils under study using BCR sequential extraction; mean ± standard deviation (*N* = 3); percentage of metal relative to the pseudo-total aqua regia metal content^a^

Metals		Measured value	Certified value	Recovery	*P* Value	Beetroot soil	Rice soil	Onion soil	Spinach	Dill	Parsley	Rhubarb	Basil	Radish	*P* Value
Al	Step 1	110.2 ± 1.5 ^a^ (1.62%)	NE	—		<LOD	2.66 ± 0.23 ^c^ 0.02%	1.006 ± 0.18 ^e^ 0.01%	2.26 ± 0.3 ^c^ 0.02%	<LOD	<LOD	6.12 ± 0.3 ^b^ 0.05%	19 ± 0.8 ^a^ 0.31%	1.2 ± 0.07 ^d^ 0.01%	<0.01
Ag		0.041 ± 0.009 (0.74%)	NE	—		<LOD	<LOD	<LOD	<LOD	<LOD	<LOD	<LOD	<LOD	<LOD	—
As		—	NE	—		0.17 ± 0.004 ^c^ 3.0%	0.047 ± 0.001 ^e^ 1.3%	0.26 ± 0.01 ^b^ 16.3%	0.24 ± 0.008 ^b^ 4.6%	0.27 ± 0.02 ^b^ 5.4%	0.24 ± 0.01 ^b^ 4.4%	0.104 ± 0.01 ^d^ 1.9%	0.13 ± 0.007 ^d^ 3.3%	0.33 ± 0.02 ^a^ 5.7%	<0.01
Ba		7.78 ± 0.07 (7.94%)	NE	—		23 ± 1 ^d^ 26%	26 ± 0.8 ^c^ 20%	23 ± 1 ^d^ 22%	35 ± 1 ^b^ 42%	22 ± 1 ^d^ 30%	22 ± 0.6 ^d^ 25%	11.6 ± 0.5 ^e^ 13%	8 ± 0.1 ^f^ 14%	37 ± 1 ^a^ 32%	0.02
Cd		6.91 ± 0.31 (53.6%)	7.3 ± 0.4	95	0.154	0.07 ± 0.004 ^c^ 18%	0.05 ± 0.005 ^d^ 12%	0.08 ± 0.003 ^b^ 17%	0.05 ± 0.003 ^d^ 15%	0.1 ± 0.003 ^a^ 38%	0.08 ± 0.006 ^bc^ 33%	0.06 ± 0.005 ^d^ 25%	0.03 ± 0.002 ^e^ 25%	0.1 ± 0.01 ^a^ 40%	<0.01
Co		1.280 ± 0.017 (16.66%)	NE	—		0.2 ± 0.003 ^c^ 1.8%	0.43 ± 0.01 ^b^ 3.1%	0.19 ± 0.008 ^c^ 1.7%	0.08 ± 0.001 ^g^ 0.67%	0.17 ± 0.01 ^d^ 1.6%	0.13 ± 0.003 ^e^ 1.1%	0.1 ± 0.006 ^f^ 0.76%	1.2 ± 0.03 ^a^ 10.2%	0.21 ± 0.007 ^c^ 1.7%	<0.01
Cr		2.34 ± 0.06 (1.03%)	2.26 ± 0.16	104	0.154	0.2 ± 0.01 ^b^ 0.19%	0.071 ± 0.003 ^f^ 0.04%	0.23 ± 0.007 ^a^ 0.22%	0.11 ± 0.005 ^d^ 0.16%	0.09 ± 0.01 ^e^ 0.15	0.087 ± 0.002 ^e^ 0.12%	0.1 ± 0.006 ^e^ 0.11%	0.15 ± 0004 ^c^ 0.15%	0.19 ± 0.01 ^b^ 0.26%	<0.01
Cu		52.15 ± 1.00 (20.61%)	49.3 ± 1.7	106	0.039	0.08 ± 0.008 ^d^ 0.19%	<LOD^e^	0.12 ± 0.008 ^c^ 0.32%	0.05 ± 0.004 ^d^ 0.19%	0.2 ± 0.002 ^a^ 0.61%	0.14 ± 0.001 ^c^ 0.55%	0.07 ± 0.006 ^d^ 0.29%	<LOD^de^	0.17 ± 0.01 ^b^ 0.85%	0.107
Fe		33.9 ± 0.7 (0.28%)	NE	—		0.97 ± 0.09 ^de^ 0.01%	0.8 ± 0.04 ^e^ 0.01%	1.2 ± 0.1 ^cd^ 0.01%	2 ± 0.1 ^b^ 0.01%	1.3 ± 0.1 ^c^ 0.01%	1.2 ± 0.1 ^c^ 0.01%	0.37 ± 0.01 ^f^ 0.002%	2 ± 005 ^b^ 0.02%	2.4 ± 0.1 ^a^ 0.02%	<0.01
Mn		78.7 ± 0.6 (36.53%)	NE	—		68 ± 3 ^cd^ 22%	62 ± 2 ^e^ 28%	57 ± 2 ^f^ 18%	40 ± 1 ^g^ 12%	77 ± 3 ^a^ 25%	72 ± 2 ^b^ 20%	65 ± 3 ^de^ 15%	62 ± 1 ^e^ 24%	63 ± 3 ^e^ 19%	0.02
Mo		0.08 ± 0.02 (4.49%)	NE	—		0.01 ± 0.001 ^e^ 2%	<LOD	0.022 ± 0.001 ^b^ 4.2%	<LOD	0.018 ± 0.001 ^c^ 4.9%	0.017 ± 0001^c^ 4.9%	<LOD	<LOD	0.026 ± 0.001 ^a^ 6.1%	<0.01
Ni		15.6 ± 0.14 (17.47%)	15.4 ± 0.9	101	0.131	1.32 ± 0.04 ^f^ 0.9%	2.24 ± 0.09 ^b^ 1.0%	1.23 ± 0.05 ^f^ 0.9%	1 ± 0.03 ^g^ 1.1%	1.6 ± 0.08 ^d^ 2.0%	1.5 ± 0.05 ^e^ 1.6%	0.9 ± 0.04 ^h^ 1.0%	2.4 ± 0.03 ^a^ 2.1%	1.8 ± 0.08 ^c^ 2.0%	<0.01
Pb		2.95 ± 0.04 (2.26%)	3.18 ± 0.21	93	0.008	<LOD	0.01 ± 0.0008 ^c^ 0.05%	<LOD	<LOD	0.01 ± 0.004 ^b^ 0.1%	<LOD	<LOD	0.021 ± 0.001 ^a^ 0.27%	<LOD	<0.01
Sb		0.094 ± 0.006 (8.79%)	NE	—		<LOD	<LOD	0.012 ± 0.001 ^c^ 7.1%	<LOD	0.016 ± 0.009 ^a^ 8.4%	0.014 ± 0.001 ^b^ 7%	<LOD	<LOD	<LOD	0.04
Se		0.068 ± 0.007 (13.33%)	NE	—		0.031 ± 0.003 ^b^ 15%	0.035 ± 0.01 ^ab^ 10%	0.019 ± 0.003 ^d^ 11%	0.025 ± 0.003 ^c^ 14%	0.033 ± 0002 ^b^ 17%	0.026 ± 0001 ^c^ 13%	0.024 ± 0.001 ^c^ 10%	<LOD	0.039 ± 0.001 ^a^ 25%	<0.01
Ti		0.199 ± 0.017 (0.03%)	NE	—		0.06 ± 0.005 ^d^ 0.01%	0.02 ± 0.001 ^e^ 0.01%	0.1 ± 0.02 ^c^ 0.01%	0.18 ± 00.02 ^a^ 0.03%	0.16 ± 0.01 ^b^ 0.03%	0.14 ± 0.01 ^b^ 0.03%	0.03 ± 00.007 ^e^ 0.004%	0.09 ± 00.004 ^cd^ 0.01%	0.11 ± 0.001 ^c^ 0.02%	0.024
Tl		0.027 ± 0.003 (11.69%)	NE	—		<LOD	<LOD	<LOD	<LOD	<LOD	<LOD	<LOD	<LOD	<LOD	—
V		0.301 ± 0.013 (1.09%)	NE	—		0.357 ± 0.006 ^f^ 0.7%	0.13 ± 0.007 ^g^ 0.3%	0.46 ± 0.01 ^d^ 0.9%	0.69 ± 0.02 ^b^ 1.6%	0.5 ± 0.01 ^c^ 1.3%	0.43 ± 0.01 ^e^ 0.9%	0.11 ± 0.005 ^g^ 0.2%	0.05 ± 0.001 ^h^ 0.1%	0.78 ± 0.03 ^a^ 1.5%	<0.01
Zn		198 ± 1.54 (44.3%)	205 ± 6	97	0.013	<LOD	<LOD	0.518 ± 0.03 ^b^ 0.53%	<LOD	0.53 ± 0.03 ^b^ 0.79%	0.17 ± 0.01 ^c^ 0.28%	<LOD	1.7 ± 0.05 ^a^ 4.0%	<LOD	<0.01
Al	Step 2	2818 ± 45.8 (41.45%)	NE	—		1163 ± 8 ^g^ 12%	1874 ± 22 ^a^ 17%	1184 ± 1 ^f^ 12%	1342 ± 17 ^d^ 13%	1362 ± 7 ^d^ 15%	1402 ± 10 ^c^ 13%	1476 ± 12 ^b^ 11%	1239 ± 13 ^e^ 20%	1057 ± 4 ^h^ 10%	<0.01
Ag		3.45 ± 0.07 (62.19%)	NE	—		<LOD	0.018 ± 0.001 ^c^ 21%	<LOD	0.13 ± 0.004 ^b^ 45%	0.25 ± 0.06 ^a^ 66%	0.13 ± 0.001 ^b^ 62%	0.129 ± 0.004 ^b^ 161%	<LOD	<LOD	<0.01
As		15.6 ± 0.3 (71.88%)	NE	—		0.153 ± 0.009 ^e^ 2.7%	0.326 ± 0.01^b^ 9.3%	0.186 ± 0.025 ^de^ 11.6%	0.225 ± 0.01 ^c^ 4.3%	0.251 ± 0.01 ^c^ 5.0%	0.19 ± 0.01 ^d^ 3.5%	0.228 ± 0.008 ^c^ 4.1%	0.485 ± 0.03 ^a^ 12.4%	0.159 ± 0.01 ^de^ 2.7%	<0.01
Ba		40.8 ± 0.56 (41.63%)	NE	—		24.7 ± 0.17 ^f^ 28%	22.68 ± 0.8 ^g^ 18%	29 ± 0.1 ^c^ 28%	27 ± 0.3 ^e^ 33%	28 ± 0.06 ^d^ 38%	30 ± 0.1 ^b^ 34%	31 ± 0.03 ^a^ 35%	15 ± 0.07 ^h^ 26%	25 ± 0.1 ^f^ 21%	<0.01
Cd		3.59 ± 0.077 (27.85%)	3.77 ± 0.28	95	0.053	0.084 ± 0.003 ^b^ 21%	0.084 ± 0.003 ^b^ 20%	0.103 ± 0.003 ^a^ 21%	0.061 ± 0.002 ^c^ 18%	0.08 ± 0.005 ^b^ 31%	0.081 ± 0.006 ^b^ 34%	0.08 ± 0.006 ^b^ 33%	0.017 ± 0.002 ^e^ 14%	0.034 ± 0.004 ^d^ 14%	<0.01
Co		2.38 ± 0.02 (30.99%)	NE	—		3.50 ± 0.06 ^f^ 31%	2.44 ± 0.04 ^h^ 18%	3.91 ± 0.03 ^e^ 34%	4.5 ± 0.01 ^c^ 38%	4.3 ± 0.0 ^d^ 40%	4.7 ± 0.02 ^b^ 38%	4.8 ± 0.04 ^a^ 36%	3.1 ± 0.03 ^g^ 26%	3.5 ± 0.05 ^f^ 28%	<0.01
Cr		39.33 ± 0.67 (17.36%)	45.7 ± 2	86	0.04	2.54 ± 0.029 ^g^ 2.4%	4.476 ± 0.1 ^b^ 2.7%	2.69 ± 0.023 ^f^ 2.5%	3.2 ± 0.05 ^d^ 4.7%	3.4 ± 0.01 ^c^ 5.5%	3 ± 0.04 ^e^ 4.2%	3.3 ± 0.02 ^c^ 3.5%	5.8 ± 0.02 ^a^ 5.7%	2.4 ± 0.04^h^ 3.3%	<0.01
Cu		124.5 ± 2.82 (9.20%)	124 ± 3	100	0.789	3.19 ± 0.08 ^f^ 7.4%	4.48 ± 0.106 ^c^ 10.9%	4.26 ± 0.04 ^d^ 11.2%	4.7 ± 0.6 ^b^ 18.3%	6.9 ± 003 ^a^ 20.9%	4.6 ± 0.01 ^bc^ 18.1%	4.5 ± 0.03 ^c^ 18.4%	4.1 ± 0.08 ^e^ 19.5%	1.9 ± 0.03 ^g^ 9.5%	<0.01
Fe		7324 ± 93 (60.72%)	NE	—		740 ± 3 ^h^ 5.2%	2722 ± 38 ^a^ 16.8%	802 ± 6 ^g^ 5.5%	1115 ± 18 ^e^ 7.6%%	1386 ± 16 ^d^ 10.4%	1047 ± 8 ^f^ 6.8%%	1999 ± 8 ^c^ 11.7	2193 ± 24 ^b^ 17.1%	666 ± 0.1 ^i^ 4.4%	<0.01
Mn		98.64 ± 1.26 (45.73%)	NE	—		155.2 ± 0.9 ^e^ 49%	45.4 ± 0.7 ^h^ 20%	166 ± 0.8 ^d^ 54%	219 ± 3 ^b^ 64%	202 ± 0.9 ^c^ 65%	252 ± 2 ^a^ 68%%	257 ± 6 ^a^ 60%	55 ± 0.4 ^g^ 21%	142 ± 0.3 ^f^ 43%	<0.01
Mo		0.139 ± 0.003 (7.81%)	NE	—		<LOD	<LOD	<LOD	<LOD	<LOD	<LOD	<LOD	<LOD	<LOD	—
Ni		25.20 ± 0.28 (28.21%)	26.6 ± 1.3	95	0.013	9.65 ± 0.14 ^g^ 6.9%	15.35 ± 0.36 ^b^ 7.2%	11.29 ± 0.09 ^f^ 8.0%	12.4 ± 0.1 ^e^ 13.9%	12.4 ± 0.1 ^e^ 15.3%	13 ± 0.06 ^d^ 14.0%	14 ± 0.2 ^c^ 15.1%	18 ± 0.1 ^a^ 15.9%	8.6 ± 0.2 ^h^ 9.8%	<0.01
Pb		121.9 ± 0.80 (93.55%)	126 ± 3	97	0.012	5.2 ± 0.022 ^f^ 39%	9.2 ± 0.1 ^b^ 50%	9.1 ± 0.1 ^b^ 43%	9.1 ± 0.1 ^b^ 68%	15 ± 0.1 ^a^ 83%	8.6 ± 0.04 ^d^ 65%	8.8 ± 0.06 ^c^ 77%	5.8 ± 0.03 ^e^ 74%	3.3 ± 0.05 ^g^ 38%	<0.01
Sb		0.147 ± 0.004 (13.74%)	NE	—		<LOD	<LOD	<LOD	<LOD	<LOD	<LOD	<LOD	<LOD	<LOD	—
Se		0.167 ± 0.01 (32.74%)	NE	—		0.035 ± 0.01 ^bc^ 17%	0.058 ± 0.01 ^a^ 16%	0.051 ± 0.007 ^abc^ 30%	0.047 ± 0008 ^abc^ 26%	0.04 ± 0.001 ^abc^ 21%	0.043 ± 0.002 ^abc^ 22%	0.06 ± 0.02 ^a^ 24%	0.055 ± 0.003 ^ab^ 35%	0.031 ± 0.1 ^c^ 20%	0.056
Ti		7.44 ± 0.43 (1.21%)	NE	—		<LOD	<LOD	<LOD	<LOD	<LOD	<LOD	<LOD	<LOD	<LOD	—
Tl		0.1 ± 0.009 (43.3%)	NE	—		<LOD	<LOD	<LOD	<LOD	<LOD	<LOD	<LOD	<LOD	<LOD	—
V		10.03 ± 0.09 (36.6%)	NE	—		9.06 ± 0.11 ^c^ 18%	11.3 ± 0.1 ^a^ 23%	10.91 ± 0.03 ^b^ 21%	8.3 ± 0.2 ^ef^ 19%	7.9 ± 0.08 ^g^ 20%	8.2 ± 0.09 ^f^ 18%	8.5 ± 0.04 ^de^ 17%	8 ± 0.05 ^g^ 18%	8.6 ± 0.08 ^d^ 17%	<0.01
Zn		100 ± 1.618 (22.37%)	114 ± 5	88	0.004	10.8 ± 0.15 ^g^ 16%	11.50 ± 0.2 ^f^ 16%	37.79 ± 0.43 ^a^ 39%	18 ± 0.3 ^d^ 31%	32 ± 0.4 ^b^ 48%	18 ± 0.1 ^d^ 30%	18.6 ± 0.02 ^c^ 37%	17 ± 0.2 ^e^ 40%	4.4 ± 007 ^h^ 10%	<0.01
Al	Step 3	1178 ± 2.9 (17.32%)	NE	—		891 ± 14 ^f^ 9.3%	1360 ± 14 ^b^ 3.3%	1049 ± 10 ^d^ 10.9%	1079 ± 3 ^d^ 10.5	986 ± 6 ^e^ 10.6%	1160 ± 7 ^c^ 10.9%	1733 ± 52 ^a^ 13.4%	395 ± 4 ^g^ 6.4%	140 ± 4 ^h^ 1.3%	<0.01
Ag		<LOD	NE	—		<LOD	<LOD	<LOD	<LOD	<LOD	<LOD	<LOD	<LOD	<LOD	—
As		0.76 ± 0.037 (3.50%)	NE	—		0.077 ± 0.003 ^d^ 1.4%	0.039 ± 0.002 ^f^ 1.1%	0.048 ± 0.002 ^e^ 3.0%	0.084 ± 0.004 ^c^ 1.6%	0.12 ± 0.003 ^a^ 2.4%	0.104 ± 0.003 ^b^ 1.9%	0.051 ± 0.001 ^e^ 0.9%	0.09 ± 0.01 ^c^ 2.3%	0.006 ± 0.001 ^g^ 0.1%	<0.01
Ba		13.68 ± 0.03 (13.96%)	NE	—		1.7 ± 0.004 ^g^ 2.0%	2.4 ± 0.01 ^c^ 1.9%	3.3 ± 0.001 ^a^ 3.2%	2.1 ± 0.03 ^d^ 2.5%	2.1 ± 0.02 ^d^ 2.9%	2.1 ± 0.02 ^e^ 2.4%	2.5 ± 0.04 ^b^ 2.8%	1.9 ± 0.03 ^f^ 3.3%	0.26 ± 0.01 ^h^ 0.2%	<0.01
Cd		0.242 ± 0.02 (1.88%)	0.27 ± 0.06	90	0.176	0.016 ± 0.001 4%	<LOD	0.012 ± 0.001 2.5%	<LOD	<LOD	<LOD	<LOD	<LOD	<LOD	—
Co		0.876 ± 0.03 (11.4%)	NE	—		0.554 ± 0.006 ^h^ 4.9%	1.6 ± 0.02 ^a^ 11.6%	0.689 ± 0.002 ^f^ 6.0%	0.73 ± 0.01 ^e^ 6.1%	0.65 ± 0.01 ^g^ 6.0%	0.82 ± 0.001 ^c^ 6.6%	1.0 ± 0.02 ^b^ 7.6	0.76 ± 0.01 ^d^ 6.4%	0.076 ± 0.005 ^i^ 0.6%	<0.01
Cr		135 ± 0.06 (59.58%)	143 ± 7	94	<0.01	9.5 ± 0.1 ^c^ 9.0%	15 ± 0.2 ^a^ 9.1%	9.9 ± 0.01 ^b^ 9.3%	7.8 ± 0.1 ^e^ 12%	7.6 ± 0.1 ^f^ 12%	8.5 ± 0.04 ^d^ 12%	7.9 ± 0.2 ^e^ 8.4%	9.6 ± 0.1 ^c^ 9.4%	0.94 ± 0.01 ^g^ 1.3%	<0.01
Cu		49.42 ± 0.2 (19.53%)	55 ± 4	90	<0.01	0.146 ± 0.01 ^fg^ 0.3	1.5 ± 0.04 ^a^ 3.7%	0.12 ± 0.01 ^g^ 0.3%	0.168 ± 0.02 ^ef^ 0.7%	0.24 ± 0.01 ^d^ 0.7%	0.198 ± 0.01 ^e^ 0.8%	0.54 ± 0.02 ^c^ 2.2%	0.75 ± 0.02 ^b^ 3.6%	<LOD	<0.01
Fe		567.2 ± 1.35 (4.70%)	NE	—		159 ± 2 ^g^ 1.1%	596 ± 6 ^a^ 3.7%	196 ± 2 ^f^ 1.4%	200 ± 1 ^ef^ 1.4%	216 ± 2 ^d^ 1.6%	206 ± 2 ^e^ 1.3%	439 ± 12 ^b^ 2.6%	312 ± 4 ^c^ 2.4%	36 ± 1 ^h^ 0.2%	<0.01
Mn		14.21 ± 0.08 (6.59%)	NE	—		14 ± 0.1 ^f^ 4.4%	18 ± 0.2 ^c^ 8.1%	15 ± 0.05 ^e^ 4.8%	17 ± 0.1 ^d^ 5.0%	15 ± 0.2 ^e^ 4.9%	19 ± 0.1 ^b^ 5.1%	20 ± 0.4 ^a^ 4.7%	11 ± 0.1 ^g^ 4.2%	1.6 ± 0.04 ^h^ 0.5%	<0.01
Mo		1.116 ± 0.033 (62.73%)	NE	—	—	0.072 ± 0.003 ^e^ 14%	0.23 ± 0.002 ^a^ 35%	0.126 ± 0.003 ^b^ 24%	0.076 ± 0.002 ^de^ 22%	0.079 ± 0.01 ^cd^ 22%	0.081 ± 0.003 ^cd^ 23%	0.084 ± 0.01 ^c^ 12%	0.13 ± 0.001 ^b^ 23%	0.076 ± 0.001 ^de^ 18%	<0.01
Ni		13.34 ± 0.13 (14.94%)	15.3 ± 0.9	87	0.002	19 ± 0.2 ^c^ 14%	30 ± 0.4 ^a^ 14%	23 ± 0.1 ^b^ 16%	14.7 ± 0.2 ^e^ 17%	12 ± 0.1 ^g^ 15%	15.4 ± 0.1 ^d^ 17%	14 ± 0.3 ^f^ 15	14.8 ± 0.3 ^e^ 13%	1.6 ± 0.02 ^h^ 2%	<0.01
Pb		8.5 ± 0.146 (6.52%)	9.3 ± 2	91	0.011	0.498 ± 0.02 ^c^ 3.7%	0.78 ± 0.03 ^b^ 4.3%	1.5 ± 0.03 ^a^ 7.1%	0.15 ± 0.003 ^e^ 1.1%	0.225 ± 0.01 ^d^ 1.2%	<LOD	0.234 ± 0.01 ^d^ 2.1%	0.112 ± 0.003 ^f^ 1.4%	0.095 ± 0.001 ^f^ 1.1%	<0.01
Sb		<LOD	NE	—		<LOD	<LOD	<LOD	<LOD	<LOD	<LOD	<LOD	<LOD	<LOD	—
Se		0.282 ± 0.01 (55.29%)	NE	—		0.054 ± 0.002 ^de^ 26%	0.173 ± 0.01 ^a^ 49%	0.032 ± 0.003 ^f^ 19%	0.069 ± 0.004 ^bc^ 38%	0.059 ± 0.004 ^cd^ 31%	0.06 ± 0.01 ^cd^ 30%	0.075 ± 0.013 ^b^ 30%	0.074 ± 0.002 ^b^ 47%	0.043 ± 0.01 ^ef^ 27%	<0.01
Ti		15.97 ± 0.3 (2.40%)	NE	—		106 ± 1.0 ^d^ 17%	83 ± 0.4 ^g^ 18%	115 ± 0.4 ^b^ 15%	80 ± 0.4 ^h^ 15%	100 ± 1 ^e^ 19%	96 ± 0.7 ^f^ 18%	137 ± 2.9 ^a^ 20%	109 ± 0.8 ^c^ 14%	13 ± 0.3 ^i^ 2%	<0.01
Tl		1.46 ± 0.19 (0.22%)	NE	—		<LOD	<LOD	<LOD	<LOD	<LOD	<LOD	<LOD	<LOD	<LOD	—
V		2.451 ± 0.02 (8.94%)	NE	—		5.0 ± 0.02 ^d^ 5%	4.1 ± 0.1 ^g^ 4.1%	5.8 ± 0.1 ^a^ 5.8%	4.9 ± 0.01 ^e^ 49%	4.3 ± 0.1 ^f^ 4.3%	5.2 ± 0.03 ^c^ 5.2%	5.8 ± 0.1 ^a^ 5.8%	5.3 ± 0.04 ^b^ 5.3%	0.67 ± 0.01 ^h^ 0.67%	<0.01
Zn		38.5 ± 0.47 (8.61%)	46 ± 4	83	<0.01	0.149 ± 0.02 ^g^ 0.2%	3.7 ± 0.1 ^a^ 5.1%	0.98 ± 0.03 ^c^ 1.0%	0.79 ± 0.05 ^e^ 1.3%	0.91 ± 0.03 ^de^ 1.4%	0.87 ± 0.03 ^cd^ 1.4%	1.2 ± 0.1 ^b^ 2.4%	0.47 ± 0.04 ^f^ 1.1%	<LOD	<0.01

a The values reported as < LOD were excluded from statistical analysis. *P* value < 0.05 is considered significant, *P* value < 0.01 is considered highly significant, and the same superscript letters indicate no significant difference. The values with different letters (a, b, c, and d) denote statistically significant differences between groups based on Duncan's multiple range test (*p* < 0.05).

The levels of Cu, Cd, Zn, and Pb in the core of beetroot observed in this study were lower than those previously reported in the literature, which was in the range of 16–62, 0.12–0.46, 21.48–49.28, and 1.22–3.18 mg kg^−1^, respectively.^34^ In this study, the levels of Cu, Cd, and Pb in the core of beetroot aligned with the reported values, while the Cr level was lower than those reported, which ranged from 0.62 to 1.96 mg kg^−1^.^35,36^ The contents of Mn, Fe, Ni and Pb in the core of beetroot in this study were lower than those previously reported in the literature, which were 257.88, 538.5, 0.7, and 3 mg kg^−1^, respectively.^36,37^

#### 3.4.2 Onion

In the plant-available fraction (Table 4), Mn revealed the highest levels (57 mg kg^−1^), corresponding to 18% of the total aqua regia contents of the soil used to farm onion species. The levels of Ag, Pb, and Tl were below the LOD. Although these metals were measured in the soil samples, this suggests that these metals are strongly associated with the stable fractions of the soil and showed relatively poor mobility. The plant-available content of Ba in the cultivated soil was also relatively high (23 mg kg; 22%), followed by Ni (1.23 mg kg; 0.9%) and then Fe (1.2 mg kg; 0.01%). The plant-available fraction of As was only 0.26 mg kg^−1^ (16.3% of the total aqua regia content), while Cr, Co, Cu, and Cd comprised 0.23 mg kg^−1^ (0.22%), 0.19 mg kg^−1^ (1.7%), 0.12 mg kg^−1^ (0.32%), and 0.08 mg kg^−1^ (17%), respectively. Among the selected metals, only Mn and Fe exhibited similar trends, as did V and Cu, while the others showed different patterns across the onion vegetable (Table 3). The concentrations of Mn and Fe in the onion revealed the corresponding descending order: leaf > stem > root > skin > bulb. It is fascinating that the bulb of an onion, which is consumed by most people, accumulated low contents of the analyzed metals compared with other compartments. The highest concentrations of As, Zn, Se, and Cd were detected in the root of onion. This indicates that the root of onion serves as an important barrier for these heavy metals, especially As and Cd, limiting the translocation of these heavy metals to reach the aerial organs. Ag, Sb, and Tl were present at higher levels in the skin of onion, accounting for 0.036 mg kg^−1^, 0.05 mg kg^−1^, and 0.02 mg kg^−1^, respectively. In addition, the stem of the onion, which acts as a conduit to transfer metals to other organs, accumulated the highest contents of V, Co, Ni, Cu, and Mo. For several metals, including Al, Ba, Ti, Cr, Mn, Fe, Co, and Pb, the highest contents were found in the leaves of onion. The accumulation of these metals in the leaves shows the ability of leaves to pre-concentrate them within the plant and effective translocation from the root to the stems. Although low contents of the plant-available fraction of Ti, Fe, Cu, Zn, and Pb were found in the soil, considerably high levels of these metals were found in certain compartments of onion tissues. This behavior reflects the ability of onion organs, especially the roots, to pre-concentrate from their environment.

The concentrations of Cr, Cu, Cd, and Pb in the onion bulbs in this study were lower than those reported in the literature. However, the Zn level in this study exceeded the reported value of 2.28 mg kg^−1^.^38^ Although the levels of As, Fe, Zn, Cd, and Pb were lower in this study compared to other works, the concentrations of Cr, Ni, and Cu were higher than the reported values in the literature for the same onion compartment, which were 1.2–1.49, 0.38–0.9, and 3.82, respectively.^2,36^ Although the Co and Cu contents in onion leaves in this study were within the range reported in the literature (LOD = 2.913 mg kg^−1^ for Co ^39^ and 1.20–8.73 mg kg^−1^ for Cu), the levels of Cr, Pb, Cd, Mn, and Fe were higher than the previously reported values, which were below the LOD for Cr, Pb, and Cd, ranged from 60.07 to 63.73 mg kg^−1^ for Mn, and ranged from 764.33 to 1836.47 mg kg^−1^ for Fe. The Zn levels in this study were lower than the previously reported range of 31.2 to 92.27 mg kg^−1^.^16^ Although the levels of Cu in the roots of onions aligned with the reported values, which ranged from <LOD to 276, the levels of Mn, Fe, and Zn in the roots of onions in this study were lower than those documented in the literature, which were 582–683, 4500–9576, and 450–1158, respectively.^40^

#### 3.4.3 Rice

The plant-available fraction of Mn demonstrated the highest levels among the analysed metals in this study, measuring 62 mg kg^−1^, which comprises 28% of the total aqua regia content in the soil used to cultivate the rice plant. However, Ag, Cu, Mo, Sb, Tl, and Zn were below the LOD, suggesting a strong affinity for retention in stable soil fractions. Ba has relatively high levels with a plant-available fraction of 26 mg kg^−1^, representing 20% of the total aqua regia content, followed by Al (2.66 mg kg; 0.02%) and then Ni (2.24 mg kg; 1%). As demonstrated a relatively low plant-available concentration (0.047 mg kg^−1^), accounting for only 1.3% of its total aqua regia-extractable content. Likewise, the plant-available fractions of Co, Cr, and Pb represented only 3.1%, 0.04%, and 0.05% of their respective total concentrations, while Cd revealed a higher availability of 12%. These results indicate that only a small fraction of the total metal quantity was readily available for plant absorption. It is interesting to note that despite the low contents of Al, As, V, Ti, Fe, and Zn in the plant-available forms, these metals were found to be relatively substantially high in the root of rice. This suggests that the root acts as a barrier, restricting translocation of these metals to the other compartments.

Rice has more of this compartmentalisation of heavy metals between the sections of the plant (Table 3). The values of Sb and Tl were below the LOD in different regions of the rice plant. The distribution patterns of Al, Ti and Pb in the rice plant were comparable, and the descending order was root > stem > leaf > grain. The distribution of Ba, Co, Ni, Cu and Cd in the rice plant was identical in the order of leaf > root > stem > grain. Ag, Fe and Se had a similar compartmentation in the rice plant: root > leaf > stem > grain. Cr and Mn showed similar distribution patterns in the rice plant with the greatest concentration in leaf (19.8 and 584 mg kg^−1^, respectively) followed by stem (4.4 and 149 mg kg^−1^), root (3.7 and 39 mg kg^−1^) and grain (3 and 31 mg kg^−1^), respectively. Zn and Mo showed a similar decrease.

It is important to note that the grain had the lowest levels of heavy metals, such as Al, Ag, Ba, Ti, Cr, Mn, Fe, Co, Ni, Cu, Se, Cd, and Pb. The leaf, however, had the highest levels of heavy metals, such as Ba, Cr, Mn, Co, Ni, Cu, Zn, Mo, Cd, and Tl. Despite the concentrations of Fe, Cu, and Zn in the rice grain being lower than those documented in the literature, the levels of Cr, Ni, and Pb were higher than those reported previously. Additionally, the amounts of Mn and Cd in the grain from this study aligned with the findings from other studies.^33^ In this study, the content of As was higher than the reported value of 0.031 to 0.075 mg kg^−1^, while the levels of Co and Ba were lower than those found in other studies, which reported ranges of 8.54 to 18.11 mg kg^−1^^17^ and 161.6 to 4535.5 mg kg^−1^,^41^ respectively.

Despite the high level of Cr in the stem of rice (4.4 mg kg^−1^) in this study being higher than the reported values (0.832 mg kg^−1^), the contents of As, Cu, Zn, Cd, and Pb in this study were higher than those reported previously, which were 3.507, 26.277, 63.79, and 1.97 mg kg^−1^, respectively. The levels of As, Cr, and Pb in rice grains in this study surpassed the previously reported values of 0.132, 0.193, and 0.098 mg kg^−1^, respectively. However, the contents of Cu, Zn, and Cd were found to be lower than the reported values of 5.241, 22.792, and 0.064 mg kg^−1^, respectively.^42^

#### 3.4.4 Spinach

The levels of “plant-available” metals varied considerably among metals (Table 4). Mn exhibited the highest content of plant-available metal of 40 g kg^−1^ (corresponding to approximately 12% of the total aqua regia extractable content). Ba also showed a relatively high fraction of available form content of 35 mg kg^−1^ (12%), followed by Al with contents of 2.26 mg kg^−1^ (0.02%), Fe (2; 0.01%), Ni (1; 1.1%), and V (0.69 mg kg; 1.6%). Interestingly, As, which is the most toxic metal in this study, represents almost 5% (0.24 g kg^−1^) of total aqua regia extractable content, while Ti and Cr exhibited 0.18 and 0.11 mg kg^−1^, respectively. In contrast, Cd, Co, Cu, and Se only weakly existed in the mobilized fraction. Ag, Mo, Pb, Sb, Tl, and Zn were below the LOD in the plant-available fraction despite their appreciable total concentrations in soil. These findings suggest that the majority of these metals were linked with stable mineral phases and therefore possessed limited mobility.

The concentrations of metals exhibited considerable differences among the vegetable compartments. For Ag and As, the roots of spinach had the highest contents, suggesting that the root compartment acted as a key shield hindering the transfer of these metals to the aerial parts. However, for metals such as Al, Ba, V, Ti, Mn, Fe, Co, Cu, Zn, Se, Mo, Cd, Sb, and Pb, the leaves of spinach accumulated the highest concentrations of these metals compared to the root and stem systems. These outcomes refer to the effective accumulation of certain metals from the root to the photosynthesized aerial parts of the spinach. In addition, the stem had the highest contents of Cr (5.8 mg kg^−1^), Ni (3.1 mg kg^−1^), and Se (0.26 mg kg^−1^). These findings align with an earlier ICP-MS investigation in spinach, which noted the greatest absorption in stems under high exposure.^43^

The compartmentalization of these heavy metals in the leaves of spinach is concerning because leaves are the edible part of this vegetable and are involved in human exposure to these metals. It is interesting to observe that despite the low plant availability of Al (0.02%), Fe (0.01%), and Ti (0.03%) with respect to the total aqua regia concentrations, spinach accumulated considerably high contents of these metals in its edible parts. The quantities detected within the plant tissues were consequently substantially higher than the soluble fraction found in the soil, suggesting a pre-concentrating impact by the root system. The contents of Cr, Ni, Cd, and Pb in different parts of spinach (root, stem, and leaf) exceeded the values reported in the literature.^43^ Some metals such as Zn, Cu, Ni, and Cd are more mobile and can be easily translocated to aerial tissues than highly held elements like Pb and Cr. This pattern is expected in leafy vegetables due to high transpiration rates, large leaf areas, and atmospheric inputs that lead to higher aerial burdens. Published studies consistently indicate spinach as one of the higher-risk vegetables for Cd, Cr, and Pb, indicating increased translocation compared to many other leafy crops.^44,45^

#### 3.4.5 Dill

Mn exhibited the highest plant-available metal in the soil used to cultivate dill (77 mg kg^−1^), corresponding to 25% of the pseudo-total aqua regia content. However, AL and Ag were below the LOD. Ba exhibited relatively high concentrations in the available fraction (22 mg kg^−1^), corresponding to 30% of the total aqua regia content, followed by Ni (1.6 mg kg^−1^, corresponding to 2%) and then Zn (0.53 mg kg^−1^, corresponding to 0.79%). As had 0.27 mg kg^−1^ of the total concentration (5.4), while Co, Cd, Pb, and Se were 0.17 mg kg^−1^ (1.6%), 0.05 (38%), 0.01 (0.1%), and 0.033 mg kg^−1^ (17%), respectively.

For Al, Ag, As, V, Co, Ni, Zn, Cd, and Sb, the root of dill demonstrated the highest values, suggesting that the roots play a vital role as a barrier limiting the translocation of these heavy metals to the stems and leaves. However, the leaves of dill accumulate high contents of Ti, Mn, Fe, Cu, Mo, Tl, and Pb compared to the stems and roots. In addition, the stem, which acts as a conduit for the transport of metals, contained the highest quantity of Cr (4.5 mg kg^−1^) and Se (0.15 mg kg^−1^). This indicates partial root retention with effective shoot transfer for some mobile micronutrients and possible leaf-surface exposure for Pb.^46^ The published literature agrees that dill, as a leafy vegetable, can show elevated burdens and even exceed Pb limits in market samples, which makes leaf-specific monitoring important .^47^ The contents of Al, As, Cr, Co, Ni, Zn, and Cd in different parts of dill in this study were higher than the values reported in the literature (only the edible part of dill), while the contents of Cu and Pb were lower than the reported values, which were 10.6 mg kg^−1^ and 0.4 mg kg^−1^, respectively.^48^

The aerial parts of dill are less likely to pose significant health concerns to humans because most toxic heavy metals are concentrated in the roots. It is important to note that the plant-available fraction of Al was below the LOD; however, the concentration of heavy metals in various parts of the dill plant was relatively high, ranging from 342 to 424 mg kg^−1^. Additionally, although Ag levels were below the LOD in the plant-available form, a small amount of Ag was detected in the roots of dill. The concentrations found in the plant tissues were significantly higher than those in the “plant–available” soluble soil fraction, indicating that the root system is effective at accumulating and concentrating these elements.

#### 3.4.6 Parsley

In the plant-available fraction of soil, Mn demonstrated the maximum levels of 72 mg kg^−1^, corresponding to 20% of total aqua regia contents. However, Al, Ag, Pb, and Tl fractions were below the LOD of the methods. Mn revealed relatively high levels, counting 22 mg kg^−1^, representing 25% of total aqua regia contents, followed by Ni (1.5 mg kg^−1^, counting 1.6%) and then Fe (1.2 mg kg^−1^, counting 0.01%). As exhibited 0.24 mg kg^−1^ as a plant-available fraction representing 4.4% of total aqua regia concentrations, Cu, Co, Cr, and Cd each exhibited approximately 0.55% (0.14 mg kg^−1^), 1.1% (0.13 mg kg^−1^), 0.12% (0.087 mg kg^−1^), and 33% (0.08 mg kg^−1^), respectively. In the plant system for Al, As, Ba, V, Cr, Fe, Co, Ni, Cu, Zn, Se, Cd, and Sb, the roots of parsley accumulated the highest concentrations of these metals, indicating the capability of this compartment to restrict the transport of these metals to the aerial parts.

This is in line with excluder behavior, in which edible shoots are partially shielded from numerous harmful substances by subterranean tissues.^49^ Although Mn (108 mg kg^−1^) and Mo (1 mg kg^−1^) demonstrated high levels in the stem, Ti (57 mg kg^−1^) and Pb (1.8 mg kg^−1^) were found to be dominant in the stem of parsley. It is interesting to note that the contents of Ag and Tl were below the LOD in the form of the plant-available fraction in the analyzed soil used for the cultivation of parsley even in its different parts. However, Al was below the LOD in the plant-available fraction and found to be at high levels in different organs of parsley, especially the roots. This can be explained by the ability of this vegetable to pre-concentrate this metal in the root and restrict its translocation to the other organs.^50^

The contents of Mn, Ni, Cu, Zn, Cd, and Pb in the roots of parsley in this study were higher than those reported in the literature, which were 3.75, 0.22, 1.35, 3.92, 0.01, and 0.11 mg kg^−1^, respectively. Additionally, the levels of these heavy metals in the leaves of parsley in this study were also higher than the values reported in the literature, which were 6.98, 0.55, 1.27, 9.41, 0.04, and 0.42 mg kg^−1^, respectively. The findings of this study indicate that As concentrations in different parts of parsley were consistently lower than those previously reported in the literature (16.6 mg kg^−1^), while iron levels across all tested parts exceeded the reference value of 564 mg kg^−1^.^51^

#### 3.4.7 Rhubarb

In the plant-available assessment, Mn exhibited the highest contents among the analyzed metals (65 mg kg^−1^), corresponding to 15% of the total aqua regia soil contents used to cultivate rhubarb. However, the contents of Ag, Mo, Pb, Sb, Tl, and Zn fractions were below the LOD. Ba revealed relatively high contents with an available fraction of 11.6 mg kg^−1^, representing 13% of total aqua regia soil content, followed by Al (6.12 mg kg^−1^) and then Ni (0.9 mg kg^−1^). As exhibited a plant-available concentration of 0.104 mg kg^−1^, representing 1.9% of its total aqua regia extraction concentration, while Cu, Cd, Co, and Cr showed relatively low contents. The primary constituents in the rhubarb roots included V, Ti, Cr, Mn, As, and Se, with their levels surpassing those found in other plant tissues. This compartmentalisation indicates that the roots function as a barrier, diminishing the translocation of these metals to the above-ground parts of the plant. Nevertheless, stems exhibited the greatest concentrations of Fe, Co, Ni, and Cu relative to the other organs. The plant leaves contain the highest levels of Zn, Mo, Cd, and Pb. It is interesting that the contents of Ag, Sb, and Tl were below the limit of detection (LOD) in both the plant-available fractions of the cultivated soil and all analyzed organs of rhubarb. In contrast, although the contents of Fe, Mo, Pb, and Zn were either below the LOD or present at low levels in the plant-available soil fraction, they compartmented to detectable concentrations in various parts of rhubarb. This reveals that rhubarb has the ability to pre-concentrate these metals from the soil and engage in long-term accumulation processes, despite their low plant-available fraction in the soil.

The contents of Fe and As in the root and Pb of different organs of rhubarb in this study were higher than the values reported in the literature, which were based on analyzing the whole plant: 25.8752 mg kg^−1^, 0.0203 mg kg^−1^, and 0.0012 mg kg^−1^, respectively. However, the contents of As, Cu, Zn, and Cd in this study were lower than those reported in the literature, which were 0.0203 mg kg^−1^, 2.1874 mg kg^−1^, 21.1148 mg kg^−1^, and 0.0049 mg kg^−1^, respectively.^52,53^

#### 3.4.8 Basil

In the plant-available fraction, Mn exhibited the highest level among the analysed metals (62 mg kg^−1^), corresponding to 24% of the total aqua regia soil content used for the cultivation of basil. However, plant-available metals such as Ag, Cu, Mo, Sb, Se, and Tl were below the LOD of the method. Al also showed a relatively high plant-available fraction (19 mg kg^−1^), representing 0.31% of the total soil aqua regia contents, followed by Ba (8 mg kg^−1^) and then Ni (2.4 mg kg^−1^), representing 2.1% of the total aqua regia content. The plant-available content of As represents 3.3% of the total aqua regia contents (0.13 mg kg^−1^), while the Cd, Co, and Cr contents were 25% (1.2 mg kg^−1^), 10.2% (1.2 mg kg^−1^), 0.15% (0.15 mg kg^−1^), respectively. The contents of Cr, Ni, Cu, and Zn in the root of basil in this study were higher than those values reported in the literature, while the contents of Co, Cd, and Pb were lower for the same compartment. In this research, the levels of Cr, Ni, Cd, and Pb in basil stems were found to be inferior to those reported in the existing literature, although the concentrations of Co, Cu, and Zn were similarly diminished compared to earlier findings. The concentrations of Cr, Ni, and Cu in basil leaves in this study surpassed those found in previous research, whereas the levels of Co, Zn, and Pb were diminished compared to the findings of Dinu *et al.* (2020). In a different investigation, the concentrations of arsenic in the roots and leaves of basil exceeded those recorded in this study, which were quantified at 0.95 mg kg^−1^ and 67.5 mg kg^−1^, respectively. Conversely, the concentrations of Mn reported in their research (67.5 mg kg^−1^ and 48.4 mg kg^−1^) were lower than those seen in this investigation.^54^

Basil demonstrated an exceptional distribution pattern among its compartments, in which the root accumulated the highest contents of all metals in this study compared to other organs. This suggests that the roots act as a barrier, hampering the translocation of these metals to the aerial parts of basil. This indicates that the aerial parts, including the stems and leaves, are safer than the roots for human consumption. It is intriguing to observe that metals such as Cu, Mo, Sb, and Se were below the LOD, and Cr, Pb, T, and Zn existed at low levels as plant-available fractions with respect to total aqua regia; basil accumulated substantial amounts of these metals. The concentrations of these metals in the plant system are substantially higher than the soluble fraction, suggesting a pre-concentration effect in the root compartment.

#### 3.4.9 Radish

Basil had a notable distribution pattern across its compartments, with the root demonstrating the highest concentration of all metals examined in this study relative to other organs. This suggests that the roots function as a hindrance to the translocation of these metals into the aerial portion of basil. This implies that the above-ground components, namely stems and leaves, are more secure for human consumption than the roots. Notably, the contents of metals including Cu, Mo, Sb, and Se were found to be below the limit of detection, whereas Cr, Pb, T, and Zn were detected at minimal concentrations as plant-accessible fractions in relation to total aqua regia. Basil amassed substantial quantities of these metals. The levels of these metals within the plant system significantly exceeded those in the soluble fraction, suggesting a pre-concentration phenomenon in the root compartment. The proportion of metal available in the plant for manganese was the most significant among the metals examined, measuring 63 mg kg^−1^, which represented 19% of the total aqua regia content in the soil utilised for radish cultivation. However, the plant-available fractions of Ag, Pb, and Sb were below the limit of detection. Ba demonstrated a notable plant-available fraction (37 mg kg^−1^), constituting 32% of the overall aqua regia extractable content. Subsequently, Fe was present at 2.4 mg kg^−1^ (0.02%) and Ni at 1.8 mg kg^−1^ (2%). The percentage of arsenic accessible to plants in the analysed soil constituted 5.7% of the overall aqua regia concentration (0.33 mg kg^−1^). In contrast, the plant-available percentages of Cd, Co, Cu, and Cr were 40% (0.1 mg kg^−1^), 1.7% (0.21 mg kg^−1^), 0.85% (0.17 mg kg^−1^), and 0.26% (0.19 mg kg^−1^), respectively. The root of parsley had the greatest levels of Zn and Sb, whereas the peel of parsley contained the highest concentrations of Co and Ni, measuring 0.868 and 6.4 mg kg^−1^, respectively. Conversely, the foliage had the greatest concentrations of only Al, Cu, and Pb. The stems, regarded as conduits for metal transport, had the highest levels of Ba, Ti, Cr, Mn, and Fe. The radish core had the most elevated levels of Ag, As, V, Se, Mo, Cd, and Tl compared to other tissues. The percentages of Ag, Pb, Sb, Tl, and Zn accessible to plants in the examined soil were below the limit of detection, while those for Al, Fe, Ti, and Zn were moderately low. This shows that the various organs of radish can pre-concentrate certain metals in specific compartments. This indicates that radish shows root-sink behaviour and a large amount of internal redistribution across the peel, core, stem and leaf, instead of using one dominant storage organ.^55^ Published vegetable surveys that include radish also reveal that metal loads vary by metal type and environmental conditions, with industrial contamination increasing the risk associated with edible tissues.^45^ In this investigation, the amounts of Al, As, Co, Ni, Cu, Zn and Cd in the radish roots were greater than those reported in the literature (47.8, 0.1, 0.6, 2.6, 3.0 and 17 mg kg^−1^, correspondingly). By comparison, the levels of Cr and Pb in the same compartment were lower at 3.9 mg kg^−1^ and 17 mg kg^−1^, respectively. In addition, the contents of Al, As, Cr, Ni, Cu, Cd and Pb in the radish leaves were greater than the values of 73.3, 0.1, 4.7, 3.0, 4.2, 0.01 and 0.4 mg kg^−1^, respectively, reported in the literature. In contrast, the amounts of Co and Zn were lower than those reported in the literature (0.5 mg kg^−1^ and 20.7 mg kg^−1^, respectively).^48^

### 3.5 “Plant-available” heavy metals

A standard BCR protocol was administered to assess the plant-available heavy metals. Since BCR 701 (European Community Bureau of Reference; the certified reference material used for validating the three-step sequential extraction procedure in soil) does not provide a certified value for all heavy metals, only the values of metals such as Cd, Cr, Cu, Ni, and Zn were measured and compared to ensure the reliability of the applied procedure (Table 4). The recoveries of metals such as Cd, Cr, Cu, Ni, and Zn from step 1 ranged from 93 to 106%. The recoveries of the same heavy metals from step 2, except Cr (86%) and Zn (88%), varied between 95% and 100%. The recoveries from step 3 for all metals except Ni (87%) and Zn (83%) changed from 90% to 94%. The BCR protocol is a useful procedure for estimating the fractionating mobility and availability of heavy metals from soil to plants. This procedure illustrates how metals are bound to soils, for example, “acid-soluble fraction—bound to carbonates,” “reducible fraction—bound to Fe and Mn oxides,” and “oxidizable fraction—bound to organic matter and sulfides,” since total metal determination is not enough for the assessment of the environmental risk and mobility of heavy metals from soils to plants.^60,61^ The maximum amount of heavy metals that could potentially be mobilized in the soil to the plants under study corresponds to the pseudo-total metal content in the soils used for cultivating vegetables in this study (Table 4).

Most metals in plant-available fraction soils used for the cultivation of vegetables were present at low concentrations. Metal plant-available fraction with respect to the total aqua regia contents of soils in the descending order ranged as follows: <LOD-0.31% for Al, <LOD for Ag, 13–42% for As, 12–40% for Ba, 12–40% for Cd, 0.67–10.2% for Co, 0.04–0.26% for Cr, <LOD-0.85% for Cu, 0.002–0.02% for Fe, 12–25% for Mn, <LOD-6.1% for Mo, 0.9–2.1% for Ni, <LOD-0.27% for Pb, <LOD-8.4% for Sb, <LOD-25% for Se, <LOD for Tl, 0.1–1.6% for V, and <LOD-4% for Zn.

In the reducible fraction (bound to Fe and Mn oxides) of all vegetable soils, the contents Ag (except in the soils used to cultivate rice, spinach, dill, parsley, and rhubarb), Mo, Sb, Ti, and Tl were below the LOD. Meanwhile, other metals, including Al, As, Ba, Cd, Co, Cr, Cu, Fe, Mn, Ni, Pb, V, and Zn, were present at high levels, indicating that most of these metals are strongly associated with Fe and Mn oxides in the soils. The proportion of these metals with respect to the pseudo-total aqua regia soil content ranged as follows: 10–20% for Al, 2.7–12.4% for As, 18–38% for Ba, 14–34% for Cd, 18–40% for Co, 2.4–5.7% for Cr, 7.4–20.9% for Cu, 4.4–17.1% for Fe, 20–68% for Mn, 6.9–15.9% for Ni, 38–83% for Pb, 16–35% for Se, 17–23% for V, and 10–48% for Zn.

In the oxidisable fraction – bound to organic matter and sulphides, the contents of Ag, Cd (except for the soil used to cultivate beetroot and onion), Sb, and Tl were below the LOD. In addition, the proportion of other metals with their respect to pseudo-total aqua regia soil content ranged as follows: 1.3–13.4% for Al, 0.1–3% for As, 0.2–3.3% for Ba, 0.6–1.6% for Co, 1.3–12% for Cr, <LOD-3.7% for Cu, 0.2–3.7% for Fe, 0.5–8.1% for Mn, 12–35% for Mo, 2–17% for Ni, <LOD-7.1% for Pb, 19–49% for Se, 2–20% for Ti, 0.67–4.9% for V, and <LOD-5.1% for Zn. The increased metal concentrations in soils can result in their absorption by plants, a process influenced not only by the metal levels in the soil but also by factors like soil pH, organic matter, clay content, cation exchange capacity, and phosphate levels. While these factors do not alter the total metal content in the soil, they greatly impact metal bioavailability. Additionally, botanical parameters including species and developmental stage influence metal absorption and translocation within the plant.^62^

### 3.6 TF

Transfer factors from soil to plant were estimated using eqn (2) and showed considerable heterogeneity across the vegetables and the plant tissues analysed, implying that metal uptake and translocation were highly dependent on the plant species and tissue (Table 5). In general, the majority of TF values were less than 1, indicating that the transport of metals from soil to plant tissues was significantly limited. Increased TF levels in some tissues indicate increased bioavailability and selective accumulation. In general, the root tissues showed higher transfer factors for many metals, due to their direct contact with the polluted soil and because they are the first site of metal uptake. The transference of Se (0.5) and Ag (6.6) was much greater in beetroot roots, but rice roots exhibited substantial accumulation of As (TF = 2.39). Moreover, the roots and stems of onions exhibited a notable transference of diverse elements including Se, Mo, and Co, indicating efficient absorption and, under specific conditions, movement from subterranean to aerial parts. Similar organ-specific patterns were seen in spinach, dill, parsley, rhubarb, basil and radish; however, the transfer magnitude was calculated from the metal-dependent variances in TF values among plant organs, which may be due to variances in root uptake, metal mobility within the plant, and the physiological activities of particular elements.

**Table 5:** Transfer factor of metals from soil to different organs of beetroot, rice, onion, spinach, dill, parsley, rhubarb, basil, and radish based on the mean concentration of metals in soils and vegetables

Vegetable/plant	Al	Ag	As	Ba	V	Ti	Cr	Mn	Fe	Co	Ni	Cu	Zn	Se	Mo	Cd	Sb	Tl	Pb
Beetroot-root	0.07	6.6	0.04	0.05	0.04	0.036	0.002	0.02	0.01	0.01	0.0001	0.02	0.05	0.50	0.31	0.03	a<LOD	0.133	0.02
Beetroot-skin	0.13	2.4	0.06	0.05	0.08	0.056	0.004	0.04	0.01	0.01	0.0003	0.04	0.05	0.16	0.04	0.02	a<LOD	a<LOD	0.02
Beetroot-core	0.05	6.8	0.03	0.08	0.02	0.040	0.002	0.03	0.00	0.01	0.0002	0.04	0.07	0.05	0.04	0.03	a<LOD	a<LOD	0.02
Beetroot-stem	0.06	3.2	0.05	0.05	0.03	0.031	0.002	0.03	0.01	0.01	0.0224	0.00	0.03	0.04	0.03	a<LOD	a<LOD	a<LOD	0.01
Beetroot-leaf	0.12	1.0	0.04	0.09	0.05	0.076	0.003	0.13	0.01	0.01	0.0002	0.04	0.05	0.07	0.08	0.03	a<LOD	a<LOD	0.03
Onion root	0.01	a<LOD	1.29	0.02	0.07	0.064	0.090	0.13	0.05	0.14	0.0663	0.18	0.29	9.20	2.24	0.26	0.24	a<LOD	0.03
Onion skin	0.02	0.9	0.09	0.14	0.01	0.041	0.018	0.09	0.01	0.02	0.0186	0.15	0.22	1.11	0.90	0.10	0.38	0.118	0.02
Onion bulb	0.02	0.6	0.09	0.14	0.01	0.037	0.016	0.09	0.01	0.02	0.0180	0.16	0.22	1.60	0.74	0.09	a<LOD	a<LOD	0.02
Onion stem	0.04	0.7	0.43	0.08	0.13	0.022	0.240	0.24	0.18	0.23	0.4530	0.32	0.27	5.60	4.48	0.12	0.35	a<LOD	0.02
Onion leaf	0.04	a<LOD	0.43	0.29	0.12	0.188	0.806	0.45	0.24	0.13	0.1640	0.19	0.23	0.31	2.19	0.12	a<LOD	a<LOD	0.07
Rice-root	0.14	3.3	2.39	0.03	0.17	0.150	0.023	0.17	0.06	0.02	0.0005	0.02	0.02	0.11	0.18	0.02	a<LOD	a<LOD	0.12
Rice-stem	0.07	0.29	0.10	0.02	0.05	0.081	0.027	0.67	0.01	0.01	0.0004	0.01	0.01	0.05	0.13	a<LOD	a<LOD	a<LOD	0.04
Rice-leaf	0.01	0.70	0.86	0.12	0.01	0.013	0.121	2.62	0.03	0.03	0.0139	0.06	0.25	0.05	0.46	0.02	a<LOD	a<LOD	0.02
Rice-whole grain	0.01	a<LOD	0.29	0.00	0.01	0.011	0.018	0.14	0.00	0.00	0.0002	0.01	0.02	0.04	0.41	a<LOD	a<LOD	a<LOD	0.01
Spinach-root	0.03	0.16	0.05	0.23	0.02	0.033	0.081	0.07	0.03	0.03	0.0312	0.27	0.58	0.73	0.87	0.49	a<LOD	a<LOD	0.02
Spinach-stem	0.03	0.16	0.05	0.24	0.03	0.029	0.086	0.07	0.04	0.04	0.0352	0.31	0.65	1.44	0.89	0.51	0.06	a<LOD	0.02
Spinach-leaf	0.10	0.08	0.03	0.32	0.03	0.056	0.041	0.19	0.07	0.04	0.0324	0.33	0.79	1.25	1.00	0.72	0.13	a<LOD	0.02
Dill root	0.05	0.05	0.07	0.27	0.03	0.044	0.060	0.08	0.05	0.03	0.0642	0.14	0.76	0.62	2.14	0.91	0.09	a<LOD	0.02
Dill stem	0.04	a<LOD	0.06	0.32	0.02	0.042	0.073	0.09	0.04	0.02	0.0531	0.10	0.59	0.78	3.33	0.88	0.04	a<LOD	0.01
Dill leaf	0.04	a<LOD	0.04	0.21	0.03	0.046	0.031	0.20	0.06	0.03	0.0520	0.14	0.68	0.54	4.43	0.84	0.06	0.317	0.02
Parsley root	0.14	a<LOD	0.06	0.41	0.08	0.071	0.149	0.13	0.08	0.05	0.0736	0.55	0.56	0.46	0.56	1.25	0.16	a<LOD	0.04
Parsley stem	0.11	a<LOD	0.04	0.30	0.04	0.105	0.082	0.16	0.07	0.05	0.0570	0.36	0.50	0.39	1.06	0.82	0.14	a<LOD	0.14
Parsley-leaf	0.04	a<LOD	0.02	0.31	0.02	0.044	0.025	0.20	0.04	0.02	0.0484	0.36	0.53	0.45	2.91	0.45	0.09	a<LOD	0.02
Rhubarb root	0.01	a<LOD	0.01	0.11	0.00	0.005	0.004	0.03	0.00	0.00	0.0141	0.08	0.08	0.12	0.02	0.05	a<LOD	a<LOD	0.02
Rhubarb skin	0.01	a<LOD	0.00	0.11	0.00	0.004	0.004	0.03	0.00	0.00	0.0142	0.08	0.08	0.12	0.02	0.05	a<LOD	a<LOD	0.01
Rhubarb stem	0.01	a<LOD	0.00	0.11	0.00	0.005	0.004	0.03	0.00	0.01	0.0150	0.08	0.08	0.06	0.03	0.06	a<LOD	a<LOD	0.01
Rhubarb leaf	0.01	a<LOD	0.00	0.10	0.00	0.004	0.004	0.03	0.00	0.00	0.0133	0.07	0.13	0.05	0.03	0.06	a<LOD	a<LOD	0.02
Basil root	0.27	a<LOD	0.22	1.40	0.22	0.113	0.176	0.30	0.21	0.14	0.1059	0.66	0.98	0.66	1.15	4.44	0.79	a<LOD	0.11
Basil stem	0.1	a<LOD	0.05	1.12	0.06	0.040	0.055	0.14	0.08	0.04	0.0370	0.37	0.57	0.14	0.41	0.09	0.20	a<LOD	0.05
Basil-leaf	0.1	a<LOD	0.06	0.71	0.04	0.043	0.044	0.20	0.09	0.04	0.0399	0.38	0.67	0.31	0.79	0.07	0.20	a<LOD	0.06
Radish root	0.04	a<LOD	0.12	0.30	0.04	0.027	0.039	0.08	0.04	0.06	0.0520	0.18	0.44	1.14	5.18	0.60	0.54	0.184	0.02
Radish skin	0.05	a<LOD	0.07	0.30	0.04	0.030	0.039	0.10	0.05	0.07	0.0732	0.15	0.34	0.90	2.18	0.47	0.42	0.070	0.02
Radish core	0.04	a<LOD	0.13	0.30	0.04	0.026	0.039	0.08	0.04	0.07	0.0510	0.17	0.42	1.21	6.16	0.80	0.30	0.424	0.02
Radish stem	0.06	a<LOD	0.05	0.44	0.03	0.050	0.085	0.16	0.06	0.06	0.0639	0.14	0.36	0.96	1.99	0.49	0.13	a<LOD	0.02
Radish leaf	0.09	a<LOD	0.03	0.13	0.04	0.045	0.073	0.11	0.06	0.04	0.0487	0.23	0.32	0.14	1.36	0.33	0.06	a<LOD	0.09

a TF < LOD/soil concentration because the corresponding plant metal concentration was below the analytical LOD.

Nutrient transport channels absorb the necessary ingredients. Non-essential or potentially harmful materials are prevented from penetrating the roots or are confined within specialist tissues. The information indicates that soil metal levels are inadequate for forecasting metal accumulation in consumable plant sections. Human exposure is significantly influenced by the specific organ consumed and the variety of plant involved. Consequently, the observed variations in transport parameters underscore the importance of accounting for plant- and organ-specific metal transport when assessing potential health hazards associated with the intake of vegetables cultivated in polluted soils.

### 3.7 Health risk assessment

The EDIs of 19 metals for various edible parts of beetroot, onion, rice, spinach, dill, parsley, rhubarb, basil, and radish species (Al, Ag, As, Ba, Cd, Co, Cr, Cu, Fe, Mn, Mo, Ni, Pb, Sb, Se, Ti, Tl, V, and Zn) were calculated using eqn (3), and are presented in SI Table 4. The average EDI values for both adults and children followed the descending order (mg kg^−1^ BW per day): Fe (3.1 × 10^−1^ and 3.5 × 10^−1^) > Al (2.8 × 10^−1^ and 3.1 × 10^−1^) > Mn (5.2 × 10^−2^ and 5.9 × 10^−2^) > Ti (1.4 × 10^−2^ and 1.6 × 10^−2^) >Ba (9.6 × 10^−3^ and 1.1 × 10^−2^) > Zn (9.3 × 10^−3^ and 1.1 × 10^−2^) > Cr (3.9 × 10^−3^ and 4.4 × 10^−3^) > Ni (2.3 × 10^−3^ and 2.6 × 10^−3^) > Cu (2.1 × 10^−3^ and 2.3 × 10^−3^) > V (9.0 × 10^−4^ and 1.0 × 10^−3^) > As (3.8 × 10^−4^ and 4.3 × 10^−4^) > Mo (3.0 × 10^−4^ and 3.4 × 10^−4^) > Pb (2.4 × 10^−4^ and 2.8 × 10^−4^) > Co (2.0 × 10^−4^ and 2.3 × 10^−4^)> Se (4.8 × 10^−5^ and 5.5 × 10^−5^) > Cd (4.0 × 10^−5^ and 4.5 × 10^−5^) > Ag (3.5 × 10^−5^ and 4.3 × 10^−5^) > Sb (1.2 × 10^−5^ and 1.3 × 10^−5^) > Tl (1.1 × 10^−5^ and 1.7 × 10^−5^). The EDI values of heavy metals differed significantly among the studied crops and plant tissues, demonstrating that the accumulation patterns were mostly dependent on the species and tissue type. Among the studied metals, Fe, Mn and Zn had generally the highest EDI values in most of the samples, while the lowest EDI levels were seen for Cd, Tl and Sb. When the EDI measurements were compared with the TDI/PTDI limitations, it was found that most of the EDI values were below the safety limits. However, continuous surveillance is needed because of high concentrations of some harmful metals in specific consumable sections of the plants.

The HQs for non-carcinogenic risks associated with Al, Ag, As, Ba, Cd, Co, Cr, Cu, Mn, Mo, Ni, Pb, Fe, Sb, Se, Ti, Tl, V, and Zn in various parts of beetroot, onion, and rice for adults and children, evaluated using eqn (4), are presented in Table 6. The quantity, frequency, and duration of exposure dominate the potential toxicity. These features show differences among individuals, and the age also influences and manipulates these features. If the HQ is smaller than 1, no apparent potential non-carcinogenic health consequences are anticipated. However, if the HQ values are greater than 1, then there is a higher likelihood of experiencing potential non-carcinogenic risks among the consumers of these products.^63^ For metals that had metal contents lower than the LOD, the HQ values were not estimated in the risk assessment.

**Table 6:** HQ values of Al, Ag, As, Ba, V, Cr, Mn, Fe, Co, Ni, Cu, Zn, Se, Mo, Cd, Sb, Tl, and Pb in adults *via* the ingestion of different parts of beetroot, onion, rice plant, spinach, parsley, dill, rhubarb, basil, and radish

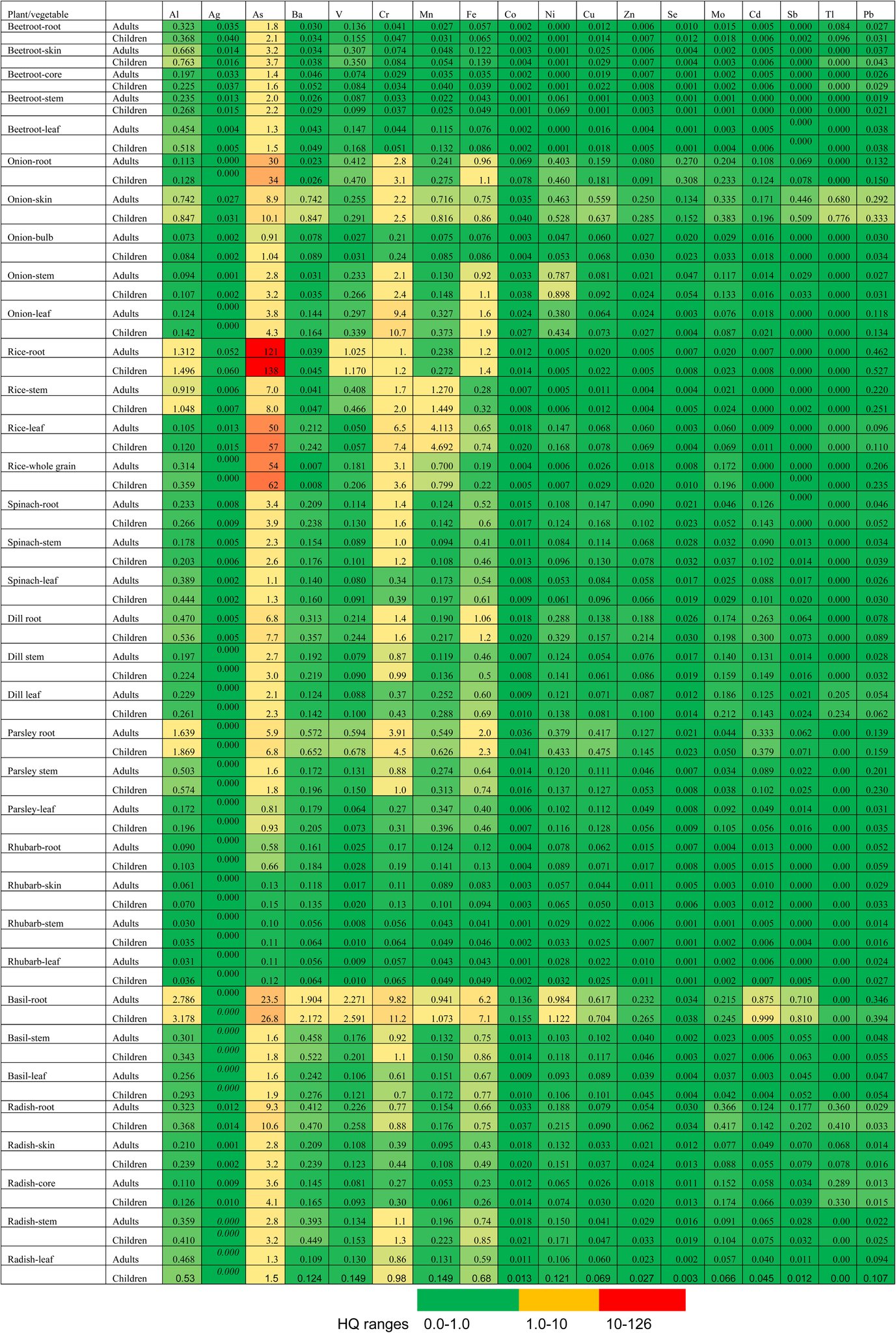

The HQ values of the consumable vegetable compartments revealed distinct variations among the examined plants, metals, and ages. As the predominant contributor to non-carcinogenic risks across all consumable parts, the HQ values exceeded the established USEPA threshold, with the highest values recorded in the edible parts of these samples, including rice grain (54 for adults and 62 for children), onion leaf (3.8 for adults and 4.3 for children), radish core (3.6 for adults and 4.1 for children), beetroot skin (3.2 for adults and 3.7 for children), onion stem, radish skin and stem (2.8 for adults and 3.2 for children), and dill stem (2.7 for adults and 3.0 for children), indicating a marked potential health risk linked with long-term exposure. Cr also contributes remarkably to the overall risk exceeding the established USEPA threshold, especially in onion leaf (9.4 for adults and 10.7 for children), rice grain (3.1 for adults and 3.6 for children), onion stem (2.1 for adults and 2.4 for children), and radish stem (1.1 for adults and 1.3 for children). Moderate HQ values were further obtained for Mn and Fe (except in onion leaves: 1.64 for adults and 1.9 for children) in some edible onion and rice tissues.

However, usually low HQ values were observed in edible parts for Cd, Sb, Tl, Pb, Co and Cu, indicating a relatively low non-carcinogenic impact. In summary, the edible rice grain had the highest HQ values among the materials studied, followed by the onion leaf and then the radish core, while edible beetroot and rhubarb tissues showed significantly lower danger levels. The results imply the possible non-carcinogenic health risk of frequently eating contaminated rice and onion products, largely due to the increased exposure to As and Cr. Moreover, the HQ values in children are higher than those in adults, which makes these vegetables more risky for youngsters. It is recommended to support the eating of vegetable skins, especially for beetroot, onion and rhubarb.

**Fig. 1:**
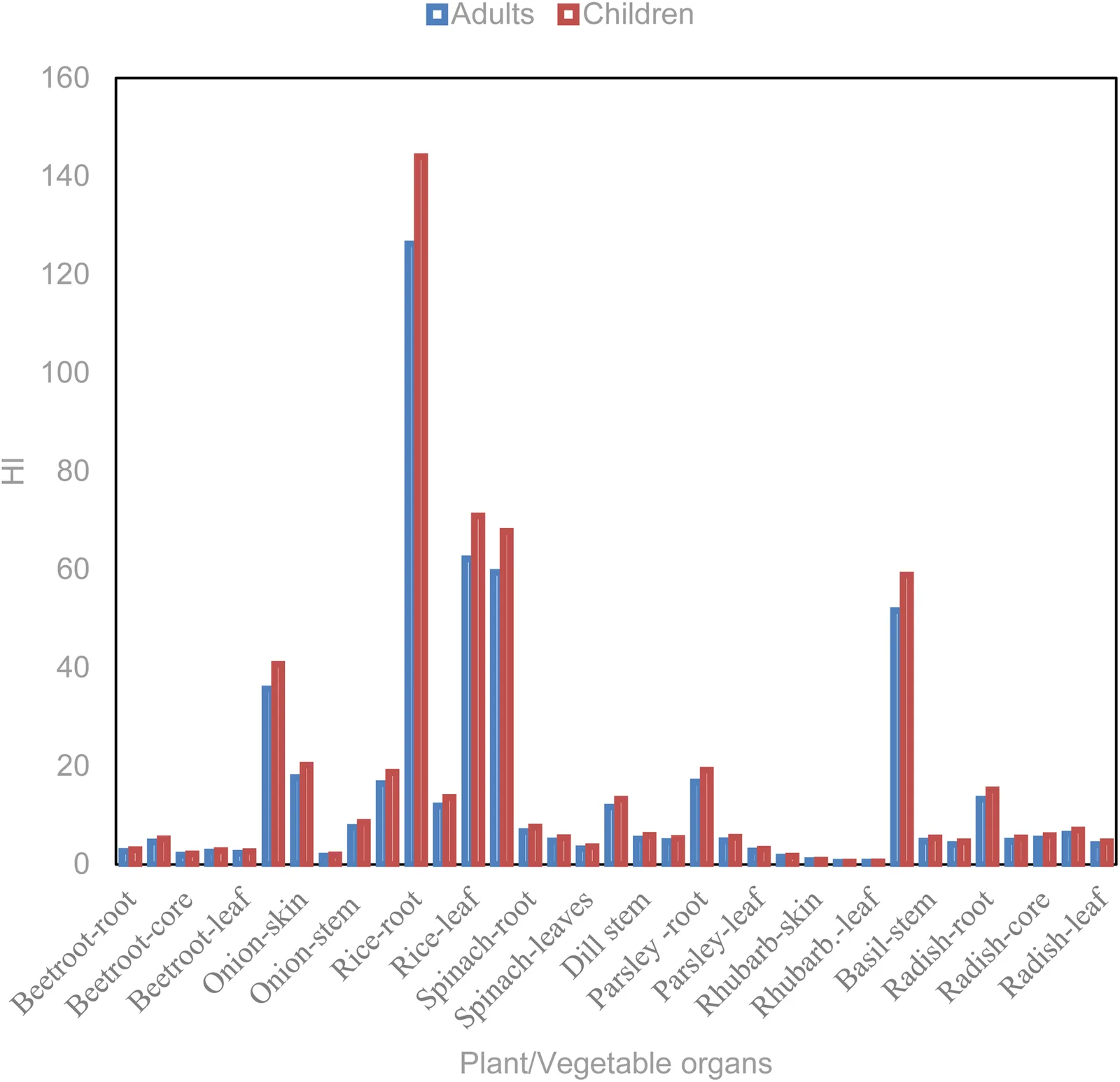
HI values for heavy metals in different parts of vegetables/plants under study.

The HI was calculated to evaluate the combined effects of multiple heavy metals, following eqn (5), as illustrated in Fig. 1. In this study, the HI values for the edible parts of vegetables and plants ranged from 0.41 for adults and 0.47 for children to 59.4 for adults and 67.7 for children. The majority (for the leaves and stems of rhubarb) of the HI values for different parts of edible vegetables/plants in this study exceeded the safety threshold of 1 for adults and children established by the USEPA. This suggests that adults and children consuming these parts of beetroot, onion, rice grain, spinach, parsley, rhubarb, dill, basil, and radish may face potential noncarcinogenic health risks. Therefore, frequent consumption of these edible plants could be detrimental to health and is not advised. The highest contributions of metals to the HI were mainly from As, Cr, and Fe in all parts of the vegetables studied. Overall, the HI values for children exceeded those of adults, indicating that youngsters are more at risk and susceptible to heavy metals. This evidence indicates that those who ingest certain vegetables/plants can be vulnerable to non-carcinogenic health effects. Therefore, we should avoid the habitual consumption of these edible plants and exclude rhubarb from our research, as they may have health hazards.

Because of the lack of cancer slope factor for Al, Ag, Ba, Co, Cu, Mn, Mo, Fe, Sb, Se, Ti, V, Tl, and Zn, the CR for adults and children was only calculated for As, Cd, Cr, Ni and Pb using eqn (6), and the data are shown in Table 7. As is marked as the key contributor to CR across all the examined samples, with all plant tissues except the leaves and stems of rhubarb revealing CR values exceeding the established acceptable USEPA threshold. The highest As-linked CR value in the edible parts of investigated samples was observed in the grain of rice (3.6 × 10^−2^ for adults and 1.0 × 10^−2^ for children), whereas the lowest value was revealed in rhubarb stem (6.4 × 10^−5^ for adults and 1.8 × 10^−5^ for children). This indicates that individuals who consume these edible parts of all vegetables and rice grain except the stem and leaves of rhubarb may be exposed to a potential lifetime risk of cancer due to the long-term As exposure.

**Table 7:** CR values of As Cr, Ni, Cd, and Pb in adults *via* the ingestion of different edible parts of beetroot, rice, onion, spinach, parsley, dill, rhubarb, basil, and radish

Plant/vegetable	As		Cr		Ni		Cd		Pb	
	Adults	Children	Adults	Children	Adults	Children	Adults	Children	Adults	Children
Beetroot-root	1.2 × 10^−3^	3.4 × 10^−4^	2.1 × 10^−5^	6.0 × 10^−6^	2.7 × 10^−6^	7.7 × 10^−7^	9.4 × 10^−7^	2.7 × 10^−7^	3.2 × 10^−7^	9.1 × 10^−8^
Beetroot-skin	2.1 × 10^−3^	6.0 × 10^−4^	3.8 × 10^−5^	1.1 × 10^−5^	6.6 × 10^−6^	1.9 × 10^−6^	9.3 × 10^−7^	2.7 × 10^−7^	4.4 × 10^−7^	1.2 × 10^−7^
Beetroot-core	9.0 × 10^−4^	2.6 × 10^−4^	1.5 × 10^−5^	4.3 × 10^−6^	2.8 × 10^−6^	8.0 × 10^−7^	8.7 × 10^−7^	2.5 × 10^−7^	3.0 × 10^−7^	8.5 × 10^−8^
Beetroot-stem	1.3 × 10^−3^	3.7 × 10^−4^	1.7 × 10^−5^	4.8 × 10^−6^	3.5 × 10^−4^	1.0 × 10^−4^	4.7 × 10^−7^	1.3 × 10^−7^	2.2 × 10^−7^	6.2 × 10^−8^
Beetroot-leaf	8.6 × 10^−4^	2.5 × 10^−4^	2.3 × 10^−5^	6.5 × 10^−6^	2.8 × 10^−6^	8.0 × 10^−7^	8.9 × 10^−7^	2.5 × 10^−7^	4.4 × 10^−7^	1.3 × 10^−7^
Rice-root	7.9 × 10^−2^	2.3 × 10^−2^	5.5 × 10^−4^	1.6 × 10^−4^	2.7 × 10^−5^	7.6 × 10^−6^	1.2 × 10^−6^	3.4 × 10^−7^	5.4 × 10^−6^	1.5 × 10^−6^
Rice-stem	4.6 × 10^−3^	1.3 × 10^−3^	9.0 × 10^−4^	2.6 × 10^−4^	3.2 × 10^−5^	9.0 × 10^−6^	3.4 × 10^−7^	9.6 × 10^−8^	2.6 × 10^−6^	7.3 × 10^−7^
Rice-leaf	3.3 × 10^−2^	9.4 × 10^−3^	3.4 × 10^−3^	9.6 × 10^−4^	8.5 × 10^−4^	2.4 × 10^−4^	1.6 × 10^−6^	4.6 × 10^−7^	1.1 × 10^−6^	3.2 × 10^−7^
Rice-whole grain	3.6 × 10^−2^	1.0 × 10^−2^	1.6 × 10^−3^	4.6 × 10^−4^	3.7 × 10^−5^	1.1 × 10^−5^	3.9 × 10^−8^	1.1 × 10^−8^	2.4 × 10^−6^	6.8 × 10^−7^
Onion-root	2.0 × 10^−2^	5.6 × 10^−3^	1.4 × 10^−3^	4.0 × 10^−4^	2.3 × 10^−3^	6.6 × 10^−4^	1.9 × 10^−5^	5.4 × 10^−6^	1.5 × 10^−6^	4.4 × 10^−7^
Onion-skin	5.9 × 10^−3^	1.7 × 10^−3^	1.1 × 10^−3^	3.2 × 10^−4^	2.7 × 10^−3^	7.6 × 10^−4^	3.0 × 10^−5^	8.5 × 10^−6^	3.4 × 10^−6^	9.7 × 10^−7^
Onion-bulb	6.0 × 10^−4^	1.7 × 10^−4^	1.1 × 10^−4^	3.1 × 10^−5^	2.7 × 10^−4^	7.7 × 10^−5^	2.7 × 10^−6^	7.8 × 10^−7^	3.4 × 10^−7^	9.8 × 10^−8^
Onion-stem	1.9 × 10^−3^	5.3 × 10^−4^	1.1 × 10^−3^	3.1 × 10^−4^	4.5 × 10^−3^	1.3 × 10^−3^	2.4 × 10^−6^	6.8 × 10^−7^	3.2 × 10^−7^	9.1 × 10^−8^
Onion leaf	2.5 × 10^−3^	7.1 × 10^−4^	4.8 × 10^−3^	1.4 × 10^−3^	2.2 × 10^−3^	6.2 × 10^−4^	3.2 × 10^−6^	9.2 × 10^−7^	1.4 × 10^−6^	3.9 × 10^−7^
Spinach-root	2.3 × 10^−3^	6.5 × 10^−4^	7.4 × 10^−4^	2.1 × 10^−4^	6.2 × 10^−4^	1.8 × 10^−4^	2.2 × 10^−5^	6.3 × 10^−6^	5.3 × 10^−7^	1.5 × 10^−7^
Spinach-stem	1.5 × 10^−3^	4.3 × 10^−4^	5.3 × 10^−4^	1.5 × 10^−4^	4.8 × 10^−4^	1.4 × 10^−4^	1.6 × 10^−5^	4.5 × 10^−6^	4.0 × 10^−7^	1.1 × 10^−7^
Spinach-leaf	7.3 × 10^−4^	2.1 × 10^−4^	1.8 × 10^−4^	5.0 × 10^−5^	3.1 × 10^−4^	8.8 × 10^−5^	1.5 × 10^−5^	4.4 × 10^−6^	3.0 × 10^−7^	8.7 × 10^−8^
Dill root	4.5 × 10^−3^	1.3 × 10^−3^	7.1 × 10^−4^	2.0 × 10^−4^	1.7 × 10^−3^	4.7 × 10^−4^	4.6 × 10^−5^	1.3 × 10^−5^	9.1 × 10^−7^	2.6 × 10^−7^
Dill stem	1.8 × 10^−3^	5.0 × 10^−4^	4.5 × 10^−4^	1.3 × 10^−4^	7.1 × 10^−4^	2.0 × 10^−4^	2.3 × 10^−5^	6.5 × 10^−6^	3.3 × 10^−7^	9.3 × 10^−8^
Dill leaves	1.4 × 10^−3^	3.9 × 10^−4^	1.9 × 10^−4^	5.5 × 10^−5^	7.0 × 10^−4^	2.0 × 10^−4^	2.2 × 10^−5^	6.2 × 10^−6^	6.3 × 10^−7^	1.8 × 10^−7^
Parsley root	3.9 × 10^−3^	1.1 × 10^−3^	2.0 × 10^−3^	5.7 × 10^−4^	2.2 × 10^−3^	6.2 × 10^−4^	5.8 × 10^−5^	1.7 × 10^−5^	1.6 × 10^−6^	4.6 × 10^−7^
Parsley-stem	1.0 × 10^−3^	2.9 × 10^−4^	4.5 × 10^−4^	1.3 × 10^−4^	6.9 × 10^−4^	2.0 × 10^−4^	1.6 × 10^−5^	4.5 × 10^−6^	2.3 × 10^−6^	6.7 × 10^−7^
Parsley-leaf	5.4 × 10^−4^	1.5 × 10^−4^	1.4 × 10^−4^	4.0 × 10^−5^	5.9 × 10^−4^	1.7 × 10^−4^	8.5 × 10^−6^	2.4 × 10^−6^	3.6 × 10^−7^	1.0 × 10^−7^
Rhubarb-root	3.8 × 10^−4^	1.1 × 10^−4^	8.5 × 10^−5^	2.4 × 10^−5^	4.5 × 10^−4^	1.3 × 10^−4^	2.3 × 10^−6^	6.6 × 10^−7^	6.0 × 10^−7^	1.7 × 10^−7^
Rhubarb.-skin	8.4 × 10^−5^	2.4 × 10^−5^	5.9 × 10^−5^	1.7 × 10^−5^	3.3 × 10^−4^	9.3 × 10^−5^	1.8 × 10^−6^	5.1 × 10^−7^	3.4 × 10^−7^	9.6 × 10^−8^
Rhubarb-stem	6.4 × 10^−5^	1.8 × 10^−5^	2.9 × 10^−5^	8.2 × 10^−6^	1.6 × 10^−4^	4.7 × 10^−5^	9.5 × 10^−7^	2.7 × 10^−7^	1.6 × 10^−7^	4.6 × 10^−8^
Rhubarb-leaf	7.0 × 10^−5^	2.0 × 10^−5^	2.9 × 10^−5^	8.3 × 10^−6^	1.6 × 10^−4^	4.6 × 10^−5^	1.0 × 10^−6^	3.0 × 10^−7^	2.8 × 10^−7^	7.8 × 10^−8^
Basil-root	1.5 × 10^−2^	4.4 × 10^−3^	5.1 × 10^−3^	1.4 × 10^−3^	5.7 × 10^−3^	1.6 × 10^−3^	1.5 × 10^−4^	4.4 × 10^−5^	4.0 × 10^−6^	1.1 × 10^−6^
Basil-stem	1.0 × 10^−3^	3.0 × 10^−4^	4.7 × 10^−4^	1.3 × 10^−4^	5.9 × 10^−4^	1.7 × 10^−4^	9.3 × 10^−7^	2.7 × 10^−7^	5.6 × 10^−7^	1.6 × 10^−7^
Basil-leaf	1.1 × 10^−3^	3.0 × 10^−4^	3.1 × 10^−4^	9.0 × 10^−5^	5.3 × 10^−4^	1.5 × 10^−4^	5.7 × 10^−7^	1.6 × 10^−7^	5.5 × 10^−7^	1.6 × 10^−7^
Radish-root	6.1 × 10^−3^	1.7 × 10^−3^	4.0 × 10^−4^	1.1 × 10^−4^	1.1 × 10^−3^	3.1 × 10^−4^	2.2 × 10^−5^	6.2 × 10^−6^	3.4 × 10^−7^	9.7 × 10^−8^
Radish core	2.4 × 10^−3^	6.7 × 10^−4^	1.4 × 10^−4^	3.9 × 10^−5^	3.7 × 10^−4^	1.1 × 10^−4^	1.0 × 10^−5^	2.9 × 10^−6^	1.5 × 10^−7^	4.3 × 10^−8^
Radish skin	1.8 × 10^−3^	5.2 × 10^−4^	2.0 × 10^−4^	5.6 × 10^−5^	7.6 × 10^−4^	2.2 × 10^−4^	8.5 × 10^−6^	2.4 × 10^−6^	1.7 × 10^−7^	4.7 × 10^−8^
Radish stem	1.8 × 10^−3^	5.2 × 10^−4^	5.6 × 10^−4^	1.6 × 10^−4^	8.6 × 10^−4^	2.5 × 10^−4^	1.1 × 10^−5^	3.3 × 10^−6^	2.6 × 10^−7^	7.4 × 10^−8^
Radish leaf	8.8 × 10^−4^	2.5 × 10^−4^	4.4 × 10^−4^	1.3 × 10^−4^	6.1 × 10^−4^	1.7 × 10^−4^	7.0 × 10^−6^	2.0 × 10^−6^	1.1 × 10^−6^	3.1 × 10^−7^

Cr showed relatively elevated levels of CR in the edible parts of vegetables and plants in this study, except beetroot and rhubarb. The highest Cr-related CR value was observed in onion leaves (4.8 × 10^−3^ for adults and 1.4 × 10^−3^ for children), whereas the lowest value measured was for the beetroot core (1.5 × 10^−5^ for adults and 4.3 × 10^−6^ for children). Ni exhibited a potential CR in the majority of vegetables in this study, except for different organs of beetroot and grains of rice. The highest value was found in the onion stem (4.5 × 10^−3^ for adults and 1.3 × 10^−3^ for children), and the lowest values were detected in the beetroot leaves (2.8 × 10^−6^ for adults and 8.0 × 10^−7^ for children). In contrast, the CR values for Cd and Pb in various parts of all vegetables and plants in this study for adults and children ranged between the negligible and acceptable levels. This suggests that the presence of these two heavy metals is less likely to cause carcinogenic risks.

A key limitation of this study that must be acknowledged is the risk assessment derived from the aggregate concentrations of heavy metals, disregarding their chemical speciation. This is especially applicable to arsenic and chromium, as the toxicity of these metals is significantly influenced by their chemical forms, with inorganic arsenic and Cr(vi) being considerably more dangerous than organic arsenic and Cr(iii).^9^ As a result, the anticipated CR values might not serve as reliable indications of the actual risk faced by clients. Furthermore, the elevated CR values for As, Cr, and Ni necessitate a more prudent explanation, as they could be influenced by conservative assumptions and the absence of speciation data.

## 4 Conclusions

In this study, the concentrations of 19 trace metals in various compartments of different vegetables and plants, together with irrigation water and cultivated soils, and the related health risks of edible parts of these samples were evaluated. The concentrations of selected heavy metals in the water used to irrigate these vegetables were low, including some metals such as Al (only in rice and onion), Ag, Cd, Pb (only in beetroot and radish), Ti (only in rice, onion, and basil), Tl, and Zn (only in beetroot and radish), which were below the LOD. However, the contents in the corresponding soils used to cultivate these vegetables and plants were exceptionally high. Despite these elevated metal levels in the cultivated soils, not all of the metals were readily plant-available in these samples—the plant-available portion compared with pseudo-total aqua regia ranged from <LOD to 42% for all vegetables in this study. In addition, despite low plant-available fractions for Al, Ag, Cu, Mo, Pb, Ti, and Zn in some soil samples, they were present at relatively high levels in the roots of the analysed vegetables. These elevated ratios indicate potential bioaccumulation, and the roots acted as a barrier restricting translocation of some metals to the aerial parts. However, other metals showed limited uptake or accumulation. Furthermore, the soil-to-plant transfer coefficient indicated that the absorption and movement of metals were significantly affected by the individual element and the specific plant tissue, with most metals demonstrating minimal transfer, whereas certain metals displayed heightened accumulation in particular organs due to the transfer factors of the majority of metals being below 1. The compartmentalization of heavy metals in analysed vegetables did not exhibit a consistent pattern; only in rice was there a common pattern for Al, Ag, Ti, and Pb, which predominantly followed the order root > stem > leaf > rice grain. This phenomenon is affected by both the plant species and the specific metals involved. In general, the predominant heavy metals in all different parts of plants were Al, Ba, V, Ti, Cr, Mn, Fe, Ni, Cu, and Zn.

From the health point of view, the HQ values for the majority of heavy metals in various portions of different vegetables in this study were less than 1, except for As, Cr, Mn and Fe. Significant differences were seen in the HQ assessment among edible vegetable tissues, metals, and ages. As was the primary contributor to non-carcinogenic risk, with the greatest HQ values observed in rice and onion tissues, especially rice grain (54 for adults and 62 for children) and onion leaf (3.8 for adults and 4.3 for children), exceeding the specified USEPA threshold of 1. Cr was also an important contributor to the overall risk especially for rice and onion samples while Mn and Fe showed moderate contributions to selected tissues. On the contrary, low HQ values were frequently obtained for Al, Ag, Ba, V, Zn, Cd, Sb, Tl, Pb, Co and Cu. Rice tissues exhibited the highest non-carcinogenic risk, followed by onion tissues indicating potential health risks related to the long-term exposure of As and Cr through dietary consumption. These vegetables are also a great concern to children compared to adults because of high HQ values in children. All the consumable parts of beetroot, onion, spinach, parsley, dill, basil, radish and rice grain except the stem and leaves of rhubarb, showed the HI values above the USEPA threshold, indicating possible non-carcinogenic hazards for both adults and children from the cumulative impact of these trace metals.

The CR examination showed considerable differences between plant/vegetable species and metals. The CR was mostly from As and all samples except rhubarb were above the allowable USEPA limit. Cr and Ni showed higher CR values in many of the consumable components except the edible parts of beetroot for Cr and edible parts of beetroot such as skin, core and leaf and rice grain for Ni. In contrast, Cd and Pb were mostly within tolerable or minimum risk levels.

## Ethical statement

The author is fully aware of the ethical responsibility of authors. The manuscript is submitted only to the Environmental Monitoring and Assessment journal for consideration. The manuscript is original and has not been published elsewhere in any form or language.

## Author contributions

The author contributed to the study's conception and design, sample collection, experimental work, data analysis, proofreading, writing, and reviewing of the manuscript.

## Conflicts of interest

The author declares no competing financial interests or personal relationships that could have influenced the work presented in this article. No competing interest exists.

## Supplementary Material

RA-OLF-D6RA05726H-s001

## Data Availability

The author confirms that the raw data supporting the findings of this study are available upon request. Supplementary information (SI) is available. See DOI: https://doi.org/10.1039/d6ra05726h. The graphical abstract was generated with the assistance of Gemini (Google) AI image generation tool based on the authors' original scientific concept and was reviewed, edited, and approved by the authors, who take full responsibility for its content.

## References

[cit1] Sadee B. A. (2025). Hum. Exp. Toxicol..

[cit2] Osae R., Nukpezah D., Darko D. A., Koranteng S. S., Mensah A. (2023). Heliyon.

[cit3] Balengayabo J. G., Kassenga G. R., Mgana S. M., Salukele F. (2024). Agric. Water Manag..

[cit4] Sadee B. A. (2022). Cihan Univ. Sci. J..

[cit5] Sadee B. A., Ali R. J. (2023). Baghdad Sci. J..

[cit6] El-Sappah A. H., Zhu Y., Huang Q., Chen B., Soaud S. A., Abd Elhamid M. A., Yan K., Li J., El-Tarabily K. A. (2024). Front. Plant Sci..

[cit7] Sadee B. A., Ali R. J. (2023). Environ. Nanotechnol., Monit. Manage..

[cit8] Shetty B. R., Jagadeesha P. B., Salmataj S. A. (2025). CyTA–J. Food.

[cit9] Sadee B. A., Galali Y., Zebari S. M. S. (2024). RSC Adv..

[cit10] Manegabe B. J., Msagati T. A. M., Ntabugi M.-M. K., Dewar J. B. (2026). Int. J. Environ. Health Res..

[cit11] Street R. A. (2012). S. Afr. J. Bot..

[cit12] Sadee B. A., Galali Y., Zebari S. M. S. (2023). RSC Adv..

[cit13] Sadee B. A., Zebari S. M. S., Galali Y., Saleem M. F. (2025). RSC Adv..

[cit14] Kfle G., Asgedom G., Goje T., Abbebe F., Habtom L., Hanes H. (2020). J. Chem..

[cit15] Bakar M. A., Bhattacherjy S. C. (2012). Hamdard Med..

[cit16] Bedassa M., Abebaw A., Desalegn T. (2017). Sel. Areas Cent. Rift Val. Oromia Reg. Ethiop. J. Hortic..

[cit17] Hasan G. M. M. A., Das A. K., Satter M. A. (2022). J. Environ. Public Health.

[cit18] Sadee B. A., Foulkes M. E., Hill S. J. (2016). Food Addit. Contam. Part A.

[cit19] Foulkes M. E., Sadee B. A., Hill S. J. (2020). J. Anal. At. Spectrom..

[cit20] Sadee B. A., Foulkes M. E., Hill S. J. (2016). Food Chem..

[cit21] Rauret G., López-Sánchez J.-F., Sahuquillo A., Barahona E., Lachica M., Ure A. M., Davidson C. M., Gomez A., Lück D., Bacon J. (2000). J. Environ. Monit..

[cit22] Zhu W., Cai J., Ma W., Li B., Feng W. (2025). Front. Mar. Sci..

[cit23] Chowdhury A. I., Shill L. C., Raihan M. M., Rashid R., Bhuiyan M. N. H., Reza S., Alam M. R. (2024). Sci. Rep..

[cit24] U.S. Environmental Protection Agency , Exposure Factors Handbook: 2011 Edition, National Center for Environmental Assessment, Washington, DC, 2011, EPA/600/R-09/052F

[cit25] Rahim H. A., Hoseini R., Hoseini Z., Abbas E. N., Kareem D. A. (2023). BMC Public Health.

[cit26] U.S. Environmental Protection Agency , Estimated Per Capita Water Ingestion and Body Weight in the United States—An Update: Based on Data Collected by the United States Department of Agriculture's 1994–1996 and 1998 Continuing Survey of Food Intakes by Individuals, Office of Water, Washington, DC, 2004, EPA-822-R-00-001

[cit27] Mohanty B. , Patel Y. and Dhamsaniya M., in ITM Web of Conferences, EDP Sciences, 2024, vol. 65, p. 2004

[cit28] Angali K. A., Farhadi M., Neisi A., Cheraghian B., Ahmadi M., Takdastan A., Dargahi A. (2025). Sci. Rep..

[cit29] Alawadhi N., Abass K., Khaled R., Osaili T. M., Semerjian L. (2024). Environ. Pollut..

[cit30] Saah S. A., Boadi N. O., Sakyi P. O., Smith E. Q. (2024). Heliyon.

[cit31] Duncan E. G., Maher W. A., Foster S. D., Mikac K. M., Krikowa F. (2014). J. Appl. Phycol..

[cit32] Ayers R. S. and Westcot D. W., Water Quality for Agriculture, Food and agriculture organization of the United Nations Rome, 1985, vol. 29

[cit33] Desalew A., Mehari B. (2023). PLoS One.

[cit34] Boluspayeva L., Jakubus M., Spychalski W., Abzhalelov A., Bitmanov Y. (2022). Int. J. Environ. Res. Public Health.

[cit35] Arsenov D., Pajevic S., Borisev M. (2014). Food Feed Res..

[cit36] Pajević S., Arsenov D., Nikolić N., Borišev M., Orčić D., Župunski M., Mimica-Dukić N. (2018). Environ. Monit. Assess..

[cit37] Habte G., Mekonen N., Desse G., Kassa G. (2023). Toxicol. Reports.

[cit38] Bawa U., AbdulHameed A. (2025). Explor. Foods Foodomics.

[cit39] Bibi N., Shah M. H., Khan N., Mahmood Q., Aldosari A. A., Abbasi A. M. (2021). Arab. J. Chem..

[cit40] Chiroma T. M., Ebewele R. O., Hymore F. K. (2014). I. Ref. J. Eng. Sci..

[cit41] Fu J., Zhou Q., Liu J., Liu W., Wang T., Zhang Q., Jiang G. (2008). Chemosphere.

[cit42] Mao C., Song Y., Chen L., Ji J., Li J., Yuan X., Yang Z., Ayoko G. A., Frost R. L., Theiss F. (2019). Catena.

[cit43] Verma S., Singh S. (2023). Int. J. Agric. Anim. Prod..

[cit44] Saleem M., Wang Y., Pierce D., Sens D. A., Somji S., Garrett S. H. (2025). Foods.

[cit45] Mithu M. M. U., Shormela S. A., Abdullah A. T. M., Mubarak M., Rahman M. M., Khan T. A., Moniruzzaman M., Islam M. S. (2026). Biol. Trace Elem. Res..

[cit46] Shahid M., Dumat C., Khalid S., Schreck E., Xiong T., Niazi N. K. (2017). J. Hazard. Mater..

[cit47] Tajdar-Oranj B., Javanmardi F., Parastouei K., Taghdir M., Fathi M., Abbaszadeh S. (2024). Biol. Trace Elem. Res..

[cit48] Maleki A., Amini H., Nazmara S., Zandi S., Mahvi A. H. (2014). J. Environ. Heal. Sci. Eng..

[cit49] Jungová M., Asare M. O., Jurasová V., Hejcman M. (2022). Plant Soil.

[cit50] Bordean D.-M. , Caba I., Iancu T., Vladut V., Cardei P., Moigradean D., Moldovan C., Alda L. and Pirvulescu L., 24th International Symposium on Analytical and Environmental Problems, 2018, pp. 83–86

[cit51] Alsafran M., Usman K., Rizwan M., Ahmed T., Al Jabri H. (2021). Front. Environ. Sci..

[cit52] Senchenko M., Pozdnyakova V. P., Stepanova M., Olenchuk E. (2021). J. Microbiol. Biotechnol. Food Sci..

[cit53] Dinu C., Vasile G.-G., Buleandra M., Popa D. E., Gheorghe S., Ungureanu E.-M. (2020). J. Soils Sediments.

[cit54] Souri M. K., Hatamian M., Tesfamariam T. (2019). Chem. Biol. Technol. Agric..

[cit55] Huang W., Zhang C., Zhu B., Liu X., Xiao H., Liu S., Shao H. (2025). Front. Plant Sci..

[cit56] THE EUROPEAN COMMISSION , COMMISSION REGULATION (EU) 2023/915 of 25 April 2023 on Maximum Levels for Certain Contaminants in Food and Repealing Regulation (EC) No 1881/2006, 2023

[cit57] WHO (2011). WHO Chron..

[cit58] WHO , Evaluations of the Joint FAO/WHO Expert Committee on Food Additives (JECFA), ALUMINIUM-Containing Food Additives. TRS 966 JECFA 74, 2011

[cit59] Codex Alimentarius Commission , Joint FAO/WHO Food Standards ProgrammeForty-eighth Session of the Codex Alimentarius Commission in November 2025. General Standard for Contaminants and Toxins in Food and Feed CXS 193-1995, Adopted in 1995, Last Amended in 2025, 2025

[cit60] Tong L., He J., Wang F., Wang Y., Wang L., Tsang D. C. W., Hu Q., Hu B., Tang Y. (2020). Chemosphere.

[cit61] Angelova L., Burduhos-Nergis D. D., Surleva A., Sandu A. V., Ilieva D., Chernev G., Vizureanu P. (2025). JOM.

[cit62] Li J., Lu Y., Shim H., Deng X., Lian J., Jia Z., Li J. (2010). J. Environ. Monit..

[cit63] Zeng F., Wei W., Li M., Huang R., Yang F., Duan Y. (2015). Int. J. Environ. Res. Public Health.

