# Supplementary material for: Trace metal contamination from water and soil to vegetables and associated human health risks
Source: RSC Adv. 2026 Sep 8;16(43):53454–76. doi: 10.1039/d6ra05726h (PMC13551557; doi:10.1039/d6ra05726h)
Supplement: Supplementary file 1 [file RA-016-D6RA05726H-s001.pdf]

Supplementary Information (SI) for RSC Advances.
This journal is © The Royal Society of Chemistry 2026

Trace Metal Contamination from Water, Soil to Vegetables and Associated Human Health Risks

Bashdar Abuzed Sadee[1,2*]

[1]Department of Food Science and Technology, College of Agricultural Engineering Sciences, Salahaddin University-Erbil, KRG-Iraq.

[2]Department of Nutrition and Dietetics, Cihan University-Erbil, Erbil, Iraq.

*Corresponding author E-mail: bashdar.sadee@su.edu.krd ; bashdarabuzed@gmail.com

ORCID:0000-0001-7588-7388

Supplementary Table 1: ICP-MS conditions used for the measurement of Al, Ag, As, B, Ba, Cd, Co, Cr, Cu, Mn, Mo, Ni, Pb, Fe, Sb, Se, Ti, Tl, V, and Zn.

ICP-MS	Agilent 7500	
	Peristaltic pump speed mL /min	1.1
	Nebulizer type	V-groove
	Spray chamber	Modified Lichte
	Radio frequency power (W)	1280
Gas flows (L min^{-1})	Coolant	11.6
	Auxiliary	0.8
	Nebulizer	0.6
H_2 addition (m/z)	Gas flow (ml/min)7 %H_2 in He	3.5
Dwell time (ms)	ICP-MS	10

Supplementary Table 2: RfD and SF values for selected heavy metals of this study.

Metal	RFD Values (mg kg^{-1} body weight/day)	Slop factor (SF) $(mg/kg/day)^{-1}$)[1]
Al	1 [2]	-
Ag	0.0047 [3]	-

As	0.00006[1]	32[1]
Ba	0.07 [2]	-
Cd	0.001 [4]	0.51[5]
Co	0.02 [4]	-
Cr	0.003 [4]	0.5 [1]
Cu	0.0371[4]	-
Fe	0.7 [2]	-
Mn	0.14 [2]	-
Ni	0.02 [6]	0.84[5]
Pb	0.004 [7]	0.0085 [5]
Se	0.005 [6]	-
Mo	0.005[8]	
V	0.007 [9]	-
Tl	0.00008[10]	-
Sb	0.0004[11]	-
Zn	0.3 [6]	-

Supplementary Table 3: Investigation of selected heavy metals (mg/kg ± standard deviation, N=3) in BW10015, NIES, and loam soil samples, with p-values determined through independent t-test analysis. P. value < 0.05 is considered significant, P. value < 0.01 is considered highly significant.

	**GBW10015**					**NIES High Cd level**					**Loam soil**				**Analytical parameters**		
Metals	Experimental value	Certified value	Recovery (%)	P. Value	RSD %	Experimental value	Certified value	Recovery (%)	P. Value	RSD %	Experimental value	Certified value	Recovery (%)	P. Value	RSD %	R^2	Procedural blank mg/kg
Al	617.7±9.7	610±6	101	0.377	1.5	1.48±0.2	(1.5)	99	0.894	13.5	39220±230	NE	-	-	0.6	0.989	0.45±0.002
Ag	0.051±0.002	NE	-	-	4.7	3.62±0.08	NE	-	-	2.4	0.095±0.001	NE	-	-	0.7	0.999	<LOD
As	0.237±0.02	0.230±0.03	103	0.578	7.4	0.149±0.003	(0.15)	99	0.958	2.6	7.84±2	7.5±1.4	105	0.107	3	0.996	<LOD
Ba	8.81±0.22	9±0.8	98	0.268	2.5	0.276±0.013	NE	-	-	4.7	57.2±0.15	NE	-	-	0.3	0.999	0.1±0.001
Cd	0.159±0.01	0.150±0.025	106	0.511	6.3	1.816±0.01	1.82±0.06	100	0.615	0.7	0.237±0.09	0.25±0.04	95	0.153	4	0.997	<LOD
Co	0.229±0.02	0.22±0.03	104	0.495	7.9	0.007±0.001	(0.007)	100	0.742	14	7.304±0.11	7.9±0.9	93	0.012	2	0.999	<LOD
Cr	1.46±0.04	1.4±0.2	104	0.161	2.8	0.0804±0.001	(0.08)	101	0.586	1.2	31.9±0.6	31±4	103	0.120	2	0.999	<LOD
Cu	8.77±0.3	8.9±0.4	99	0.542	3.5	4.25±0.11	4.1±0.3	104	0.147	2.6	11.23±0.6	12.4±0.9	91	0.101	5	0.999	0.11±0.004
Fe	546±4.21	540±20	101	0.132	0.7	11.84±0.05	11.4±0.8	104	0.05	0.5	14070±129	NE	-	-	0.9	0.992	3±0.1
Mn	43±0.7	41±3	105	0.032	1.5	35.6±0.02	40.1±2	89	<0.01	0.06	381±6	387±17	99	0.208	2	0.993	0.2±0.01
Mo	0.506±0.03	0.47±0.04	108	0.173	5.9	1.629±0.04	NE	-		0.3	0.47±0.03	NE	-	-	7	0.999	0.09±0.003
Ni	0.892±0.01	0.92±0.12	97	0.073	1.5	0.314±0.013	0.3±0.03	105	0.197	4.1	22.9±0.38	21.9±1.6	105	0.05	2	0.999	<LOD
Pb	12.1 ± 0.17	11.1±0.9	109	0.009	1.4	0.313±0.002	NE	-	-	0.6	34.9±0.07	32.2±1.4	108	0.03	3	0.986	<LOD
Sb	0.042±0.01	0.043±0.014	102	0.969	23	0.005±0.001	NE	-	-	20	0.35±0.007	NE	-	-	2	0.999	<LOD
Se	0.089±0.004	0.092±0.024	97	0.267	4.4	0.081±0.002	(0.07)	116	0.018	3.3	0.45±0.03	NE	-	-	7	0.994	<LOD
Ti	26.0± 0.317	(28)	93	0.385	1.2	0.517±0.05	NE	-	-	9.6	463.7±10.6	NE	-	-	2	0.999	0.5±0.007
Tl	0.05±0.001	(0.049)	102	0.423	2.1	<LOD	NE	-	-	-	0.091±0.002	NE	-	-	3	0.985	<LOD
V	0.795±0.04	0.87±0.23	91	0.064	4.3	0.008±0.001	NE	-	-	8.9	37.74±0.31	NE	-	-	1	0.999	<LOD
Zn	33.2±0.44	35.3±1.5	94	0.015	1.3	20.78±0.09	23.1±0.8	90	<0.01	0.4	45.9±0.9	50±4	92	0.017	2	0.994	1±0.001
In * (%)	100±1.36	-	-	-	1.4	98.7±3.6	-	-	-	3.6	100±1.36	-	-	-	1.4		
Ir * (%)	100±1.38	-	-	-	1.4	98.9±0.4	-	-	-	0.4	105±0.9	-	-	-	0.8	-	-

Note: **NE:** Not established. (): Proposed values. * In and Ir were used to show the instrument performance which is based on recovery of adding 10 μg/L for both In and Ir.

Supplementary Table 4: EDI (mg/kg body weight/day) of Al, Ag, As, Ba, Cd, Co, Cr, Cu, Fe, Mn, Mo, Ni, Pb, Sb, Se, Ti, Tl, V, and Zn in adults via the ingestion of different parts of beetroot, onion, rice, spinach, dill, parsley, rhubarb, basil, and radish.

Vegetable		Al	Ag	As	Ba	V	Ti	Cr	Mn	Fe	Co	Ni	Cu	Zn	Se	Mo	Cd	Sb	Tl	Pb
Beetroot-root	Adults	3.2E-01	1.6E-04	1.1E-04	2.1E-03	9.5E-04	1.1E-02	1.2E-04	3.8E-03	4.0E-02	3.4E-05	9.4E-06	4.4E-04	1.7E-03	5.1E-05	7.7E-05	5.4E-06	<LOD	6.7E-06	1.1E-04
	Children	3.7E-01	1.9E-04	1.2E-04	2.4E-03	1.1E-03	1.3E-02	1.4E-04	4.4E-03	4.5E-02	3.9E-05	1.1E-05	5.1E-04	2.0E-03	5.9E-05	8.8E-05	6.1E-06	<LOD	7.7E-06	1.2E-04
Beetroot-skin	Adults	6.7E-01	6.7E-05	1.9E-04	2.4E-03	2.1E-03	1.9E-02	2.2E-04	6.7E-03	8.5E-02	6.5E-05	2.3E-05	9.3E-04	1.9E-03	1.9E-05	1.2E-05	5.3E-06	<LOD	<LOD	1.5E-04
	Children	7.6E-01	7.6E-05	2.2E-04	2.7E-03	2.5E-03	2.2E-02	2.5E-04	7.6E-03	9.7E-02	7.4E-05	2.6E-05	1.1E-03	2.1E-03	2.1E-05	1.3E-05	6.1E-06	<LOD	<LOD	1.7E-04
Beetroot-core	Adults	2.0E-01	1.5E-04	8.2E-05	3.2E-03	5.2E-04	1.1E-02	8.8E-05	4.9E-03	2.4E-02	3.6E-05	9.8E-06	7.2E-04	2.2E-03	4.7E-06	9.0E-06	5.0E-06	<LOD	<LOD	1.0E-04
	Children	2.2E-01	1.8E-04	9.4E-05	3.6E-03	5.9E-04	1.3E-02	1.0E-04	5.6E-03	2.8E-02	4.1E-05	1.1E-05	8.2E-04	2.5E-03	5.4E-06	1.0E-05	5.7E-06	<LOD	<LOD	1.2E-04
Beetroot-stem	Adults	2.3E-01	6.3E-05	1.2E-04	1.8E-03	6.1E-04	7.5E-03	9.8E-05	3.1E-03	3.0E-02	2.6E-05	1.2E-03	2.2E-05	8.7E-04	2.9E-06	5.9E-06	<LOD	<LOD	<LOD	7.5E-05
	Children	2.7E-01	7.2E-05	1.3E-04	2.0E-03	7.0E-04	8.6E-03	1.1E-04	3.6E-03	3.4E-02	3.0E-05	1.4E-03	2.5E-05	1.0E-03	3.3E-06	6.8E-06	<LOD	<LOD	<LOD	8.5E-05
Beetroot-leaf	Adults	4.5E-01	1.9E-05	7.9E-05	3.0E-03	1.0E-03	1.8E-02	1.3E-04	1.6E-02	5.3E-02	3.4E-05	9.7E-06	5.9E-04	1.3E-03	5.7E-06	1.6E-05	5.1E-06	<LOD	<LOD	1.5E-04
	Children	5.2E-01	2.1E-05	9.0E-05	3.4E-03	1.2E-03	2.1E-02	1.5E-04	1.8E-02	6.0E-02	3.9E-05	1.1E-05	6.8E-04	1.4E-03	6.5E-06	1.8E-05	5.8E-06	<LOD	<LOD	1.5E-04
Onion -root	Adults	1.1E-01	<LOD	1.8E-03	1.6E-03	2.9E-03	4.2E-02	8.3E-03	3.4E-02	6.7E-01	1.4E-03	8.1E-03	5.9E-03	2.4E-02	1.3E-03	1.0E-03	1.1E-04	2.7E-05	<LOD	5.3E-04
	Children	1.3E-01	<LOD	2.0E-03	1.9E-03	3.3E-03	4.8E-02	9.4E-03	3.8E-02	7.7E-01	1.6E-03	9.2E-03	6.7E-03	2.7E-02	1.5E-03	1.2E-03	1.2E-04	3.1E-05	<LOD	6.0E-04
Onion-skin	Adults	1.1E-01	1.3E-04	1.8E-03	1.6E-03	2.9E-03	4.2E-02	8.3E-03	3.4E-02	6.7E-01	1.4E-03	8.1E-03	5.9E-03	2.4E-02	1.3E-03	1.0E-03	1.1E-04	2.7E-05	5.4E-05	5.3E-04
	Children	8.5E-01	1.5E-04	6.1E-04	5.9E-02	2.0E-03	1.2E-01	7.6E-03	1.1E-01	6.0E-01	8.0E-04	1.1E-02	2.4E-02	8.5E-02	7.6E-04	1.9E-03	2.0E-04	2.0E-04	6.2E-05	1.3E-03
Onion-bulb	Adults	7.4E-01	8.5E-06	5.3E-04	5.2E-02	1.8E-03	1.1E-01	6.6E-03	1.0E-01	5.3E-01	7.0E-04	9.3E-03	2.1E-02	7.5E-02	6.7E-04	1.7E-03	1.7E-04	<LOD	<LOD	1.2E-03
	Children	8.4E-02	9.7E-06	6.2E-05	6.3E-03	2.2E-04	1.2E-02	7.3E-04	1.2E-02	6.0E-02	7.6E-05	1.1E-03	2.5E-03	9.1E-03	1.2E-04	1.6E-04	1.8E-05	<LOD	<LOD	1.3E-04
Onion-stem	Adults	7.3E-02	6.5E-06	5.5E-05	5.5E-03	1.9E-04	1.0E-02	6.4E-04	1.0E-02	5.3E-02	6.6E-05	9.4E-04	2.2E-03	8.0E-03	1.0E-04	1.4E-04	1.6E-05	6.2E-07	<LOD	1.2E-04
	Children	1.1E-01	7.5E-06	1.9E-04	2.5E-03	1.9E-03	4.7E-03	7.2E-03	2.1E-02	7.3E-01	7.6E-04	1.8E-02	3.4E-03	7.3E-03	2.7E-04	6.6E-04	1.6E-05	1.3E-05	<LOD	1.2E-04
Onion -leaf	Adults	9.4E-02	<LOD	1.7E-04	2.2E-03	1.6E-03	4.1E-03	6.3E-03	1.8E-02	6.4E-01	6.6E-04	1.6E-02	3.0E-03	6.4E-03	2.3E-04	5.8E-04	1.4E-05	<LOD	<LOD	1.1E-04
	Children	1.4E-01	<LOD	2.6E-04	1.2E-02	2.4E-03	5.3E-02	3.2E-02	5.2E-02	1.3E+00	5.4E-04	8.7E-03	2.7E-03	8.2E-03	2.0E-05	4.3E-04	2.1E-05	<LOD	<LOD	5.4E-04
Rice-root	Adults	1.2E-01	3.1E-07	2.3E-04	1.0E-02	2.1E-03	4.7E-02	2.8E-02	4.6E-02	1.2E+00	4.8E-04	7.6E-03	2.4E-03	7.2E-03	1.7E-05	3.8E-04	1.8E-05	6.2E-07	<LOD	4.7E-04
	Children	1.0E+00	3.5E-05	4.8E-04	3.3E-03	3.3E-03	5.0E-02	6.0E-03	2.0E-01	2.2E-01	1.5E-04	1.3E-04	4.6E-04	1.3E-03	2.4E-05	1.2E-04	1.6E-07	7.0E-07	<LOD	1.0E-03
Rice-stem	Adults	9.2E-01	3.0E-05	4.2E-04	2.9E-03	2.9E-03	4.4E-02	5.2E-03	1.8E-01	2.0E-01	1.3E-04	1.1E-04	4.0E-04	1.1E-03	2.1E-05	1.0E-04	<LOD	6.2E-07	<LOD	8.8E-04
	Children	1.0E+00	3.5E-05	4.8E-04	3.3E-03	3.3E-03	5.0E-02	6.0E-03	2.0E-01	2.2E-01	1.5E-04	1.3E-04	4.6E-04	1.3E-03	2.4E-05	1.2E-04	1.6E-07	7.0E-07	<LOD	1.0E-03
Rice-leaf	Adults	1.3E+00	2.5E-04	7.2E-03	2.8E-03	7.2E-03	5.9E-02	3.2E-03	3.3E-02	8.5E-01	2.5E-04	9.2E-05	7.3E-04	1.4E-03	3.4E-05	1.0E-04	6.9E-06	6.2E-07	1.3E-05	1.8E-03
	Children	1.2E-01	6.9E-05	3.4E-03	1.7E-02	4.0E-04	6.7E-03	2.2E-02	6.6E-01	5.2E-01	4.1E-04	3.4E-03	2.9E-03	2.1E-02	2.0E-05	3.4E-04	1.1E-05	7.0E-07	1.5E-05	4.4E-04
Rice-grain	Adults	9.2E-01	<LOD	4.2E-04	2.9E-03	2.9E-03	4.4E-02	5.2E-03	1.8E-01	2.0E-01	1.3E-04	1.1E-04	4.0E-04	1.1E-03	2.1E-05	1.0E-04	<LOD	6.2E-07	<LOD	8.8E-04
	Children	3.6E-01	<LOD	3.7E-03	5.8E-04	1.4E-03	1.8E-02	1.1E-02	1.1E-01	1.6E-01	9.1E-05	1.5E-04	1.1E-03	6.1E-03	4.8E-05	9.8E-04	<LOD	7.0E-07	<LOD	9.4E-04
Spinach-root	Adults	2.3E-01	3.6E-05	2.1E-04	1.5E-02	8.0E-04	1.4E-02	4.3E-03	1.7E-02	3.7E-01	3.0E-04	2.2E-03	5.5E-03	2.7E-02	1.0E-04	2.3E-04	1.3E-04	1.4E-05	<LOD	1.8E-04
	Children	2.7E-01	4.1E-05	2.4E-04	1.7E-02	9.1E-04	1.6E-02	4.9E-03	2.0E-02	4.2E-01	3.4E-04	2.5E-03	6.2E-03	3.1E-02	1.2E-04	2.6E-04	1.4E-04	1.6E-05	<LOD	2.1E-04
Spinach-stem	Adults	1.8E-01	2.5E-05	1.4E-04	1.1E-02	6.2E-04	8.3E-03	3.1E-03	1.3E-02	2.8E-01	2.3E-04	1.7E-03	4.2E-03	2.0E-02	1.4E-04	1.6E-04	9.0E-05	5.1E-06	<LOD	1.4E-04
	Children	2.0E-01	2.9E-05	1.6E-04	1.2E-02	7.1E-04	9.4E-03	3.6E-03	1.5E-02	3.2E-01	2.6E-04	1.9E-03	4.8E-03	2.3E-02	1.6E-04	1.8E-04	1.0E-04	5.8E-06	<LOD	1.6E-04
Spinach-leaf	Adults	3.9E-01	8.8E-06	6.7E-05	9.8E-03	5.6E-04	1.1E-02	1.0E-03	2.4E-02	3.7E-01	1.6E-04	1.1E-03	3.1E-03	1.7E-02	8.3E-05	1.3E-04	8.8E-05	7.0E-06	<LOD	1.0E-04
	Children	4.4E-01	1.0E-05	7.6E-05	1.1E-02	6.4E-04	1.3E-02	1.2E-03	2.8E-02	4.3E-01	1.8E-04	1.2E-03	3.6E-03	2.0E-02	9.5E-05	1.4E-04	1.0E-04	8.0E-06	<LOD	1.2E-04
Dill root	Adults	4.7E-01	2.2E-05	4.1E-04	2.2E-02	1.5E-03	2.6E-02	4.1E-03	2.7E-02	7.4E-01	3.5E-04	5.8E-03	5.1E-03	5.6E-02	1.3E-04	8.7E-04	2.6E-04	2.6E-05	<LOD	3.1E-04
	Children	5.4E-01	2.5E-05	4.6E-04	2.5E-02	1.7E-03	3.0E-02	4.7E-03	3.0E-02	8.5E-01	4.0E-04	6.6E-03	5.8E-03	6.4E-02	1.5E-04	9.9E-04	3.0E-04	2.9E-05	<LOD	3.6E-04
Dill stem	Adults	2.0E-01	<LOD	1.6E-04	1.3E-02	5.5E-04	1.3E-02	2.6E-03	1.7E-02	3.2E-01	1.4E-04	2.5E-03	2.0E-03	2.3E-02	8.5E-05	7.0E-04	1.3E-04	5.8E-06	<LOD	1.1E-04
	Children	2.2E-01	<LOD	1.8E-04	1.5E-02	6.3E-04	1.5E-02	3.0E-03	1.9E-02	3.7E-01	1.6E-04	2.8E-03	2.3E-03	2.6E-02	9.7E-05	8.0E-04	1.5E-04	6.6E-06	<LOD	1.3E-04
Dill -leaf	Adults	2.2E-01	<LOD	1.2E-04	8.7E-03	6.1E-04	1.4E-02	1.1E-03	3.5E-02	4.2E-01	1.8E-04	2.4E-03	2.6E-03	2.6E-02	6.0E-05	9.3E-04	1.3E-04	8.3E-06	<LOD	2.2E-04
	Children	2.6E-01	<LOD	1.4E-04	9.9E-03	7.0E-04	1.6E-02	1.3E-03	4.0E-02	4.8E-01	2.1E-04	2.8E-03	3.0E-03	2.9E-02	6.8E-05	1.1E-03	1.4E-04	9.5E-06	<LOD	2.5E-04
Parsley -root	Adults	1.6E+00	<LOD	3.6E-04	4.0E-02	4.2E-03	4.2E-02	1.2E-02	7.7E-02	1.4E+00	7.2E-04	7.6E-03	1.5E-02	3.8E-02	1.0E-04	2.2E-04	3.3E-04	2.5E-05	<LOD	5.6E-04
	Children	1.9E+00	<LOD	4.1E-04	4.6E-02	4.7E-03	4.8E-02	1.3E-02	8.8E-02	1.6E+00	8.2E-04	8.7E-03	1.8E-02	4.4E-02	1.2E-04	2.5E-04	3.8E-04	2.8E-05	<LOD	6.4E-04
Parsley stem	Adults	5.0E-01	<LOD	9.3E-05	1.2E-02	9.2E-04	2.6E-02	2.6E-03	3.8E-02	4.5E-01	2.9E-04	2.4E-03	4.1E-03	1.4E-02	3.5E-05	1.7E-04	8.9E-05	8.8E-06	<LOD	8.1E-04
	Children	5.7E-01	<LOD	1.1E-04	1.4E-02	1.0E-03	2.9E-02	3.0E-03	4.4E-02	5.1E-01	3.3E-04	2.7E-03	4.7E-03	1.6E-02	4.0E-05	1.9E-04	1.0E-04	1.0E-05	<LOD	9.2E-04
Parsley-leaf	Adults	1.7E-01	<LOD	4.9E-05	1.3E-02	4.5E-04	1.1E-02	8.2E-04	4.9E-02	2.8E-01	1.2E-04	2.0E-03	4.2E-03	1.5E-02	4.0E-05	4.6E-04	4.9E-05	5.6E-06	<LOD	1.2E-04
	Children	2.0E-01	<LOD	5.6E-05	1.4E-02	5.1E-04	1.2E-02	9.3E-04	5.5E-04	3.2E-01	1.4E-04	2.3E-03	4.7E-03	1.7E-02	4.6E-05	5.2E-04	5.6E-05	6.4E-06	<LOD	1.4E-04

Rhubarb-root	Adults	9.0E-02	<LOD	3.5E-05	1.1E-02	1.7E-04	3.9E-03	5.0E-04	1.7E-02	8.1E-02	7.8E-05	1.6E-03	2.3E-03	4.5E-03	3.5E-05	2.0E-05	1.3E-05	2.6E-06	<LOD	2.1E-04
	Children	1.0E-01	<LOD	3.9E-05	1.3E-02	2.0E-04	4.5E-03	5.7E-04	2.0E-02	9.2E-02	8.9E-05	1.8E-03	2.6E-03	5.2E-03	3.9E-05	2.3E-05	1.5E-05	3.0E-06	<LOD	2.4E-04
Rhubarb-skin	Adults	6.1E-02	<LOD	7.7E-06	8.3E-03	1.2E-04	2.6E-03	3.4E-04	1.2E-02	5.8E-02	5.6E-05	1.1E-03	1.6E-03	3.3E-03	2.5E-05	1.5E-05	1.0E-05	1.0E-06	<LOD	1.2E-04
	Children	7.0E-02	<LOD	8.8E-06	9.4E-03	1.4E-04	2.9E-03	3.9E-04	1.4E-02	6.6E-02	6.4E-05	1.3E-03	1.9E-03	3.8E-03	2.9E-05	1.7E-05	1.2E-05	1.2E-06	<LOD	1.3E-04
Rhubarb-Stem	Adults	3.0E-02	<LOD	5.8E-06	3.9E-03	5.9E-05	1.3E-03	1.7E-04	6.1E-03	2.8E-02	2.7E-05	5.7E-04	8.2E-04	1.7E-03	5.8E-06	7.4E-06	5.4E-06	1.3E-06	<LOD	5.6E-05
	Children	3.5E-02	<LOD	6.7E-06	4.5E-03	6.7E-05	1.5E-03	1.9E-04	6.9E-03	3.2E-02	3.1E-05	6.5E-04	9.3E-04	2.0E-03	6.7E-06	8.5E-06	6.2E-06	1.5E-06	<LOD	6.3E-05
Rhubarb-leaf	Adults	3.1E-02	<LOD	6.4E-06	3.9E-03	6.1E-05	1.3E-03	1.7E-04	6.0E-03	3.0E-02	2.7E-05	5.6E-04	8.2E-04	3.0E-03	5.1E-06	9.1E-06	6.0E-06	1.9E-06	<LOD	9.4E-05
	Children	3.6E-02	<LOD	7.3E-06	4.5E-03	7.0E-05	1.5E-03	1.9E-04	6.8E-03	3.4E-02	3.1E-05	6.4E-04	9.4E-04	3.4E-03	5.8E-06	1.0E-05	6.8E-06	2.2E-06	<LOD	1.1E-04
Basil-root	Adults	2.8E+00	<LOD	1.4E-03	1.3E-01	1.6E-02	1.5E-01	2.9E-02	1.3E-01	4.4E+00	2.7E-03	2.0E-02	2.3E-02	7.0E-02	1.7E-04	1.1E-03	8.8E-04	2.8E-04	<LOD	1.4E-03
	Children	3.2E+00	<LOD	1.6E-03	1.5E-01	1.8E-02	1.7E-01	3.4E-02	1.5E-01	5.0E+00	3.1E-03	2.2E-02	2.6E-02	7.9E-02	1.9E-04	1.2E-03	1.0E-03	3.2E-04	<LOD	1.6E-03
Basil-stem	Adults	3.0E-01	<LOD	9.5E-05	3.2E-02	1.2E-03	1.6E-02	2.8E-03	1.8E-02	5.3E-01	2.5E-04	2.1E-03	3.8E-03	1.2E-02	1.1E-05	1.2E-04	5.3E-06	2.2E-05	<LOD	1.9E-04
	Children	3.4E-01	<LOD	1.1E-04	3.7E-02	1.4E-03	1.8E-02	3.1E-03	2.1E-02	6.0E-01	2.9E-04	2.4E-03	4.3E-03	1.4E-02	1.3E-05	1.3E-04	6.1E-06	2.5E-05	<LOD	2.2E-04
Basil-leaf	Adults	2.6E-01	<LOD	9.7E-05	1.7E-02	7.4E-04	1.4E-02	1.8E-03	2.1E-02	4.7E-01	1.8E-04	1.9E-03	3.3E-03	1.2E-02	2.0E-05	1.9E-04	3.3E-06	1.8E-05	<LOD	1.9E-04
	Children	2.9E-01	<LOD	1.1E-04	1.9E-02	8.4E-04	1.6E-02	2.1E-03	2.4E-02	5.4E-01	2.0E-04	2.1E-03	3.7E-03	1.3E-02	2.2E-05	2.1E-04	3.8E-06	2.1E-05	<LOD	2.2E-04
Radish- root	Adults	3.2E-01	5.9E-05	5.6E-04	2.9E-02	1.6E-03	1.2E-02	2.3E-03	2.2E-02	4.6E-01	6.5E-04	3.8E-03	2.9E-03	1.6E-02	1.5E-04	1.8E-03	1.2E-04	7.1E-05	2.9E-05	1.2E-04
	Children	3.7E-01	6.7E-05	6.4E-04	3.3E-02	1.8E-03	1.4E-02	2.6E-03	2.5E-02	5.3E-01	7.4E-04	4.3E-03	3.4E-03	1.9E-02	1.7E-04	2.1E-03	1.4E-04	8.1E-05	3.3E-05	1.3E-04
Radish-skin	Adults	2.1E-01	6.5E-06	1.7E-04	1.5E-02	7.6E-04	6.8E-03	1.2E-03	1.3E-02	3.0E-01	3.6E-04	2.6E-03	1.2E-03	6.3E-03	5.8E-05	3.8E-04	4.9E-05	2.8E-05	5.5E-06	5.7E-05
	Children	2.4E-01	7.4E-06	1.9E-04	1.7E-02	8.6E-04	7.7E-03	1.3E-03	1.5E-02	3.5E-01	4.1E-04	3.0E-03	1.4E-03	7.2E-03	6.6E-05	4.4E-04	5.5E-05	3.2E-05	6.2E-06	6.5E-05
Radish-core	Adults	1.1E-01	4.2E-05	2.1E-04	1.0E-02	5.7E-04	4.2E-03	8.1E-04	7.5E-03	1.6E-01	2.4E-04	1.3E-03	9.8E-04	5.4E-03	5.5E-05	7.6E-04	5.8E-05	1.4E-05	2.3E-05	5.1E-05
	Children	1.3E-01	4.8E-05	2.4E-04	1.2E-02	6.5E-04	4.8E-03	9.2E-04	8.5E-03	1.8E-01	2.8E-04	1.5E-03	1.1E-03	6.1E-03	6.3E-05	8.7E-04	6.6E-05	1.6E-05	2.6E-05	5.9E-05
Radish-stem	Adults	3.6E-01	<LOD	1.7E-04	2.8E-02	9.4E-04	1.5E-02	3.3E-03	2.7E-02	5.2E-01	3.7E-04	3.0E-03	1.5E-03	8.7E-03	8.1E-05	4.6E-04	6.5E-05	1.1E-05	<LOD	8.9E-05
	Children	4.1E-01	<LOD	1.9E-04	3.1E-02	1.1E-03	1.7E-02	3.7E-03	3.1E-02	5.9E-01	4.2E-04	3.4E-03	1.7E-03	1.0E-02	9.3E-05	5.2E-04	7.5E-05	1.3E-05	<LOD	1.0E-04
Radish-leaf	Adults	4.7E-01	<LOD	8.0E-05	7.7E-03	9.1E-04	1.2E-02	2.6E-03	1.8E-02	4.2E-01	2.3E-04	2.1E-03	2.2E-03	7.0E-03	1.1E-05	2.9E-04	4.0E-05	4.3E-06	<LOD	3.8E-04
	Children	5.3E-01	<LOD	9.1E-05	8.7E-03	1.0E-03	1.4E-02	2.9E-03	2.1E-02	4.7E-01	2.6E-04	2.4E-03	2.5E-03	8.0E-03	1.2E-05	3.3E-04	4.6E-05	5.0E-06	<LOD	4.3E-04
TDI/PTDI (mg/kg bw/day)		0.286 [1]	NE	0.003[2]	0.2	NE	NE	0.3 [3]	0.11 [4]	0.8 [5]	NE	0.013	0.07 [6]	0.36 [7]	0.004 [8]	0.01	0.0004 [9]	0.01	NE	NE [10]
Reference		[12]		[13]	[14]			[15]	[16]	[17]		[18]	[19]	[20]	[21]	[22]	[23]	[24]		

[1] based on tolerable weekly intake (TWI) of 2 mg/kg bw/week for Al, [2]a benchmark dose lower confidence limit ($BMDL_{0.5}$), [3] This value is based on TDI for Cr(III), [4] This value was derived from tolerable upper intake of 8 mg/day for Mn established by EFSA, [5] the value is based on provisional maximum tolerable daily intake (PMTDI) established by WHO, [6] acceptable daily intake (ADI) established by EFSA, [7] The value is established as the tolerable upper intake level (UL) for Zn at 25 mg/day in food for a 70 Kg adults, [8] The value is established as the tolerable upper intake level (UL) for Se at 255 µg/day in food for a 70 Kg adults, [9] calculated based on TWI of 2.5 µg/kg bw/week for Cd, [10] EFSA concluded that no TWI could be established and the FAO/WHO withdrew the PTDI for Pb from the JECFA PTWI of 25 µg/kg body weight/week. TWI: tolerable weekly intake, NE: Not established, TDI: tolerable daily intake, UL: upper level intake.

References

1 United State Environmental Protection Agency, IRIS Toxicological Review of Inorganic Arsenic; CASRN 7440-38-2, https://iris.epa.gov/ChemicalLanding/&substance_nmbr=278.

2 B. A. Sadee and R. J. Ali, *Baghdad Sci. J.*, 2023, **20**, 715.

3 J. E. Bob Heitzman, Human Health Noncarcinogen Fact Sheet for Silver: (Human Health Noncarcinogen - fish ingestion only), Ohio Environmental Protection Agency, https://www.epa.gov/gliclearinghouse/human-health-noncarcinogen-fact-sheet-silver-human-health-noncarcinogen-fish-0.

4 F. M. Kusin, N. N. M. Azani, S. N. M. S. Hasan and N. A. Sulong, *Catena*, 2018, **165**, 454–464.

5 S. Kumar and Anshumali, *Discov. Soil*, 2025, **2**, 33.

6 I. D. de Souza, E. S. P. Melo, V. A. Nascimento, H. S. Pereira, K. R. N. Silva, P. R. Espindola, P. F. S. Tschinkel, E. M. Ramos, F. J. M. Reis and I. B. Ramos, *Biomed Res. Int.*, 2021, **2021**, 6678931.

7 A. S. Likuku and G. Obuseng, International Conference on Plant, Marine and Environmental Sciences, 2015.

8 U.S. Environmental Protection Agency, ntegrated Risk Information System (IRIS) Chemical Assessment Summary, National Center for Environmental Assessment, chrome-extension://efaidnbmnnnibpcajpcglclefindmkaj/https://iris.epa.gov/static/pdfs/0425_summary.pdf.

9 J. E. Bob Heitzman, Human Health Noncarcinogen Fact Sheet for Strontium: (Human Health Noncarcinogen - fish ingestion only), Ohio Environmental Protection Agency, https://www.epa.gov/gliclearinghouse/human-health-noncarcinogen-fact-sheet-strontium-human-health-noncarcinogen-fish.

10 A. A. JohnPaul, L. T. Ayodeji, X. Tangfu, Z. Ning and Y. Liu, *Int. J. Environ. Anal. Chem.*, 2022, **102**, 5391–5412.

11 United States Agency for Toxic Substances and Disease Registry (ATSDR), in *TitleToxicological Profile for Antimony and Compounds*, United States Department of Health and Human Services (HHS), Atlanta, 2019, pp. 158–161.

12 EFSA, *EFSA J.*, 2011, **9**, 2157.

13 WHO, *Overview on Aresnic, Evaluations of the Joint FAO/WHO Expert Committee on Food Additives (JECFA)*, 2025.

14 European Comission, 2012.

15 EFSA, *EFSA J.*, 2014, **12**, 3595.

16 EFSA, *EFSA J.*, 2023, **21**, e8413.

17 WHO, *Evaluation of certain food additives: Twenty-seventh report of the Joint FAO/WHO Expert Committee on Food Additives (WHO Technical Report Series No. 696). World Health Organization.*, 1983.

18 CONTAM, *Efsa J.*, 2020, **18**, e06268.

19 EFSA, *Total copper intakes below new safe level. Combined exposure to copper from all sources does not pose health concerns for the EU population, say EFSA's scientific experts.*, 2023.

20 EFSA, *Overview on Tolerable Upper Intake Levels as derived by the Scientific Committee on Food (SCF) and the EFSA Panel on Dietetic Products, Nutrition and Allergies (NDA)*, 2025.

21 EFSA, *EFSA J.*, 2023, **21**, e07704.
22 N. EFSA Panel on Dietetic Products and Allergies (NDA), *EFSA J.*, 2013, **11**, 3333.
23 EFSA, *EFSA sets lower tolerable intake level for cadmium in food*, 2009.
24 WHO, *WHO Chron.*, 2011, **38**, 104–108.
